# Employing CRISPR-Cas9 to Generate CD133 Synthetic Lethal Melanoma Stem Cells

**DOI:** 10.3390/ijms23042333

**Published:** 2022-02-20

**Authors:** Cynthia M. Simbulan-Rosenthal, Yogameenakshi Haribabu, Sahar Vakili, Li-Wei Kuo, Havens Clark, Ryan Dougherty, Ryyan Alobaidi, Bonnie Carney, Peter Sykora, Dean S. Rosenthal

**Affiliations:** 1 Department of Biochemistry and Molecular & Cellular Biology, Georgetown University School of Medicine, Washington, DC 20057, USA; simbulac@georgetown.edu (C.M.S.-R.); yh577@georgetown.edu (Y.H.); sv457@georgetown.edu (S.V.); lk702@georgetown.edu (L.-W.K.); hhc28@georgetown.edu (H.C.); rdd40@georgetown.edu (R.D.); raa125@georgetown.edu (R.A.); bonnie.c.carney@medstar.net (B.C.); peters@ameliatechnologies.com (P.S.); 2 Firefighters’ Burn and Surgical Laboratory, MedStar Health Research Institute, Washington, DC 20010, USA; 3 Amelia Technologies, LLC, 1121 5th St. NW, Washington, DC 20001, USA

**Keywords:** melanoma stem cells, CD133, AKT, CRISPR/cas9 knockdown, trametinib

## Abstract

Malignant melanoma is a lethal skin cancer containing melanoma-initiating cells (MIC) implicated in tumorigenesis, invasion, and drug resistance, and is characterized by the elevated expression of stem cell markers, including CD133. The siRNA knockdown of CD133 enhances apoptosis induced by the MEK inhibitor trametinib in melanoma cells. This study investigates the underlying mechanisms of CD133’s anti-apoptotic activity in patient-derived BAKP and POT cells, harboring difficult-to-treat NRAS^Q61K^ and NRAS^Q61R^ drivers, after CRISPR-Cas9 CD133 knockout or Dox-inducible expression of CD133. MACS-sorted CD133(+) BAKP cells were conditionally reprogrammed to derive BAKR cells with sustained CD133 expression and MIC features. Compared to BAKP, CD133(+) BAKR exhibit increased cell survival and reduced apoptosis in response to trametinib or the chemotherapeutic dacarbazine (DTIC). CRISPR-Cas9-mediated CD133 knockout in BAKR cells (BAKR-KO) re-sensitized cells to trametinib. CD133 knockout in BAKP and POT cells increased trametinib-induced apoptosis by reducing anti-apoptotic BCL-xL, p-AKT, and p-BAD and increasing pro-apoptotic BAX. Conversely, Dox-induced CD133 expression diminished apoptosis in both trametinib-treated cell lines, coincident with elevated p-AKT, p-BAD, BCL-2, and BCL-xL and decreased activation of BAX and caspases-3 and -9. AKT1/2 siRNA knockdown or inhibition of BCL-2 family members with navitoclax (ABT-263) in BAKP-KO cells further enhanced caspase-mediated apoptotic PARP cleavage. CD133 may therefore activate a survival pathway where (1) increased AKT phosphorylation and activation induces (2) BAD phosphorylation and inactivation, (3) decreases BAX activation, and (4) reduces caspases-3 and -9 activity and caspase-mediated PARP cleavage, leading to apoptosis suppression and drug resistance in melanoma. Targeting nodes of the CD133, AKT, or BCL-2 survival pathways with trametinib highlights the potential for combination therapies for NRAS-mutant melanoma stem cells for the development of more effective treatments for patients with high-risk melanoma.

## 1. Introduction

Malignant melanoma remains a formidable health challenge, with an estimated 106,110 new cases and 7180 deaths in the United States in 2021 and a 27% five-year survival rate for distant metastasis [1]. Drug and immune resistance, together with invasion and metastasis, determine tumor progression and ultimately, patient survival. With the highest mortality rate among skin cancers, cutaneous melanoma is associated with driver mutations in the MAPK and other signaling pathways including RAS/PI3K/AKT, p16INK4a/CDK4/RB, WNT, and/or p53 [2]. Melanomagenesis is correlated with activating driver mutations primarily in codons V600 or K601 of BRAF (*v-raf*; murine sarcoma viral oncogene homolog B1; 50% of melanomas) or Q61 of NRAS (neuroblastoma RAS viral oncogene homolog; >20%), or LOF mutations in the tumor suppressor NF1 (neurofibromin 1; 10%). 

The use of targeted kinase inhibitors, including BRAF and MEK inhibitors, combined with immune checkpoint inhibitors, has significantly improved the progression-free and overall survival of melanoma patients [3,4]. Multiple FDA-approved kinase inhibitors currently in use for melanoma include a combination of the MEK inhibitor (MEKi) trametinib (GSK1120212) and BRAF inhibitor dabrafenib for BRAFV600 mutations. Unfortunately, many patients develop resistance, significantly reducing efficacy in response to these therapeutics [5,6], a process accelerated by high mutation rates of cutaneous melanomas compared to other solid tumors [7,8]. Although *BRAF*-mutant melanoma is currently treatable with BRAF and MEK inhibitors in combination therapies, acquired resistance in ~75% of melanomas can occur by reactivation of MAPK signaling after *BRAF* copy number gains and alternative splicing, or MEK1/2 and NRAS gain-of-function mutations; an additional 20% of BRAF inhibitor-resistant melanomas acquire mutations or upregulate compensatory PI3K/AKT survival pathways [9]. Although treatment for recalcitrant *NRAS*-mutant metastatic tumors has recently advanced for PDL1-expressing patients, with immunotherapies anti–PD-1, anti-PD-L1, and anti-CTLA4 proving efficacious in first-line treatments, a majority of patients remain unresponsive, as chemotherapy with dacarbazine (DTIC), temozolomide, or carboplatin added as supplementary treatments show limited success [10]. 

While trametinib looks promising for improving patient survival from *BRAF* mutant melanoma [11], a majority of patients with the *NRAS* mutation do not respond to MEKi-based therapy alone [12]; only 28% of patients with *NRAS*-mutated melanoma achieve a stable disease with trametinib treatment [13]. Trametinib (and other second and third generation MEKi’s including binimetinib, selumetinib, pimasertib, and cobimetinib) are FDA-approved and are currently being explored for *NRAS* mutant melanoma [14] in combination with other targeted agents and immunotherapies. Clinical trials using MAPKi’s, including trametinib, plus inhibitors of the PI3K-AKT-mTOR pathway have been tested, but toxicity made it difficult to achieve therapeutic concentrations [15,16], requiring intermittent PI3Ki therapy [17]. Since *NRAS* induces cyclin D1, CDK4/6 inhibitors are being used with MEKi (palbociclib + trametinib; {NCT02065063}). Other preclinical models for combination MEKi therapy include deletion of F-Box protein 42 [18], metformin (mouse xenograft), as well as BCL-2 inhibitor ABT-263/navitoclax + trametinib (NCT02079740) [19], the same combination included in our current study. Strong preclinical anti-tumor activity with *NRAS*-mutant melanoma has also been shown after TANK-binding kinase 1 knockdown (TBK1-KD) plus MEKi (AZD6244/Selumetinib) [20]; ROCK inhibition plus trametinib [21]; ERβ activation plus trametinib [22]; as well as inhibition of Polo-like-kinase (PLK1) with MEKi (trametinib + volasertib) [23]. MEKi also upregulates melanoma antigen expression, even in BRAF-WT melanoma, allowing them to work with immune checkpoint inhibitors in mouse models [24]. There is therefore a compelling rationale to examine whether trametinib may be used in combination with inhibition of other pathways, especially those that have not been well exploited, such as CD133, or its downstream effectors.

Resistance to therapeutic combinations and melanoma recurrence have been attributed to subpopulations of “melanoma initiating cells” (MIC). These highly tumorigenic cancer stem cells are characterized by their ability to form melanospheres [25] and express receptors, cell adhesion molecules, or other stem cell markers, including ABCB5 [26], CD133 [27], CD20 [28], CD44 [29], CD144 [30], activated leukocyte cell adhesion molecule (ALCAM) [31], low affinity nerve growth factor receptor (LNGFR) [32], aldehyde dehydrogenase 1 (ALDH1) [33], Nestin [34], Tie1 [35], and JARID1B H3K4 demethylase [36]. Melanoma cells expressing CD133, ABCB5, and/or CD144 form stem cell niches facilitating tumor blood supply [7]. We have also shown elevated expression of CD133 and ABCB5 in lymph nodes and distant metastasis in various stages of human cutaneous melanoma [27]. 

The transmembrane glycoprotein CD133, known as prominin1 (PROM1), is expressed in stem cells of normal neuronal and glial cells, hematopoietic cells, endothelial cells, and cells from the adult kidney, mammary gland, salivary gland, placenta, trachea, testes, uterus, epidermis, and intestine [37,38,39,40,41,42]. CD133 is likewise overexpressed in cancer stem cells from tumors of the brain, ovary, liver, prostate, pancreas, and colon, in addition to melanoma [43,44,45,46,47,48,49,50,51]. Displaying stem cell properties of self-renewal and potency, cancer stem cells are assayed by serial propagation as tumors in immunocompromised mice [39,46,52]. However, the existence of melanoma stem cells can be model-specific [53] and melanomas possess microenvironment-regulated phenotypic plasticity [54,55,56], thus, the less controversial term “melanoma-initiating cells” (MIC) is now used instead. Nonetheless, we have shown that CD133(+) MIC are associated with drug resistance [57] as well as invasion and metastasis [58]. CD133-overexpressing glioma [59], as well as melanoma cells, may be drug-resistant in part due to the induction of ABCB1 [57]. CD133 may therefore contribute to worse outcomes and reduced patient survival and is thus an attractive therapeutic target [27]. 

Resistance of CD133(+) and CD133(−) melanoma cells to MAPKi was examined by exposing separated CD133(+) and CD133(−) subpopulations to trametinib and/or dabrafenib. CD133(+) cells exhibited significantly higher IC50s for mono and dual MAPKi treatments. We showed a causal relationship between CD133 and drug resistance by siRNA knockdown in different melanoma cell lines, indicating that CD133 confers drug resistance in melanoma [58]. However, the mechanistic pathway leading from CD133 expression to increased drug resistance has not been clarified, although it was suggested that alterations in levels or modification of apoptotic proteins (e.g., BCL-2 family members) by CD133-mediated pathways may represent a mechanism of resistance. Better delineation of the molecular mechanisms that control apoptosis is key to developing novel targets for therapeutic intervention in melanoma.

In this study, we used CRISPR-cas9 technology to analyze the potential molecular mechanisms by which CD133 is involved in the development of increased cell survival and resistance against the MEKi trametinib. CD133-overexpressing conditionally reprogrammed MIC cells showed increased cell viability and reduced apoptosis in response to trametinib. CRISPR-Cas9 knockout of CD133 reverted this phenotype in different patient-derived melanoma cell lines, sensitizing cells to trametinib. In contrast, Dox-inducible expression of CD133 attenuated trametinib-induced apoptosis and elevated levels of anti-apoptotic pAKT and BCL-2 family proteins. CD133 may therefore play a crucial role in drug resistance against current targeted therapies, through activation of an AKT-BCL-2-mediated pathway, and is thus a compelling target for intervention.

## 2. Results

### 2.1. CD133 Overexpression in Conditionally Reprogrammed BAKP Melanoma Cells (BAKR) Increases Cell Viability and Inhibits Apoptosis in Response to Trametinib or DTIC

When patient-derived melanoma cells harboring a BRAF^WT^/NRAS^Q61K^ driver mutation (BAK parental cells; BAKP) were magnetically sorted into CD133(+) and CD133(−) subpopulations, CD133 expression in the CD133(+) cells diminished after two weeks of subculture. ROCKi Y27632 + J2 feeder-mediated conditional reprogramming of CD133(+) cells allowed sustained CD133 expression (~85% CD133-positivity) together with increased expression of the stem cell markers OCT4, Nanog, and vimentin, markers of epithelial-mesenchymal transition (EMT), as well as hallmarks of melanoma initiating cells (MICs), such as melanosphere formation and resistance to MAPK inhibitors [58]. Immunoblot analysis with anti-CD133 and qPCR analysis verified CD133 overexpression in the BAKR compared to BAKP cells [58]. 

**Figure 1:**
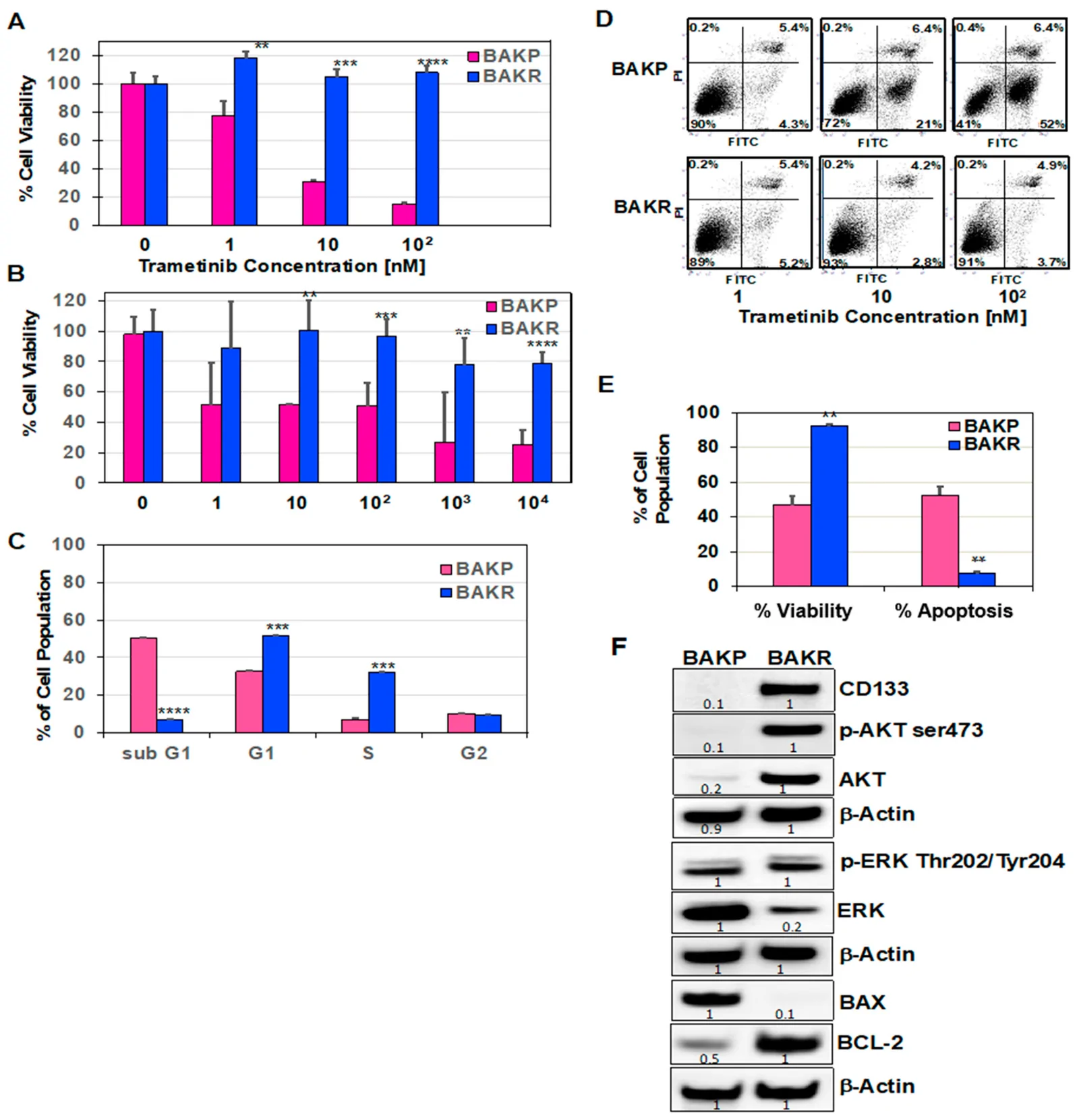
Sustained CD133 overexpression in BAKR cells increases cell viability and resistance to trametinib- and DTIC-induced apoptosis. CD133(+) BAKR melanoma cells and parental BAKP cells were exposed to increasing doses of trametinib (**A**) or DTIC (**B**) for 72 h, followed by XTT cell viability assays to assess drug sensitivity. (**C**) Cell cycle analysis of BAKR and BAKP cells after treatment with 100 nM trametinib for 72 h. (**D**) Representative dot plots of cells treated with indicated doses of trametinib and subjected to Annexin-FITC/PI staining and flow cytometry; and (**E**) % cell viability and apoptosis of cells in D. (**F**) Basal expression levels of pro-survival or pro-apoptotic proteins were compared in BAKP and BAKR cells by immunoblot analysis with antibodies to CD133, activated p-AKT (ser473), AKT, p-ERK (Thr202/Tyr204), ERK, BCL-2, BAX, and β-actin as loading control. Densitometric analysis comparing intensities of protein bands relative to bands with the highest intensity is shown in immunoblots. Scans of whole gel immunoblots for all Figures are shown in “Supplementary Materials” (Figure S1). Results shown are the means ± SD of three replicates of a representative experiment; essentially the same results were obtained in three independent experiments. **, ***, **** represent *p* < 0.01, *p* < 0.001, and *p* < 0.0001, respectively.

The role(s) of CD133 in drug resistance was examined by treating BAKP and BAKR cells with increasing doses of trametinib or the chemotherapeutic DTIC for 72 h. XTT cell viability assays to assess drug sensitivity revealed that, while trametinib induced a dose-dependent decline in cell viability in BAKP comprised primarily of CD133(−) cells, CD133-overexpressing BAKR cells were resistant to the drugs, exhibiting significantly higher cell viability after exposure to trametinib (Figure 1A) or DTIC (Figure 1B). Effects of CD133 overexpression on trametinib-induced apoptosis were next assessed by cell cycle analysis following exposure to 100 nM trametinib for 72 h. Whereas 50% of BAKP cells displayed sub-G1 DNA content, indicative of apoptotic fragmentation after trametinib treatment, the sub-G1 population decreased to 5% in CD133-overexpressing BAKR cells, along with a five-fold increase in the proliferative S-phase population (Figure 1C). After exposure to increasing doses of trametinib for 72 h, cells were also subjected to Annexin-FITC/PI staining followed by flow cytometry to detect phosphatidylserine on the surface of early apoptotic cells and loss of membrane integrity in late apoptotic cells. Consistent with the XTT cell viability and cell cycle assays, flow cytometry revealed significantly higher cell viability (lower left quadrant) and suppression of trametinib-induced apoptosis (right quadrants) in BAKR compared to BAKP cells (Figure 1D,E). 

To elucidate possible mechanisms for the differential responses, AKT and ERK survival pathways, as well as basal expression of anti-apoptotic BCL-2 and pro-apoptotic BCL-2-associated X protein (BAX) was assessed by immunoblot analysis. BAX is a key pro-apoptotic member of the BCL-2 family essential for activation of the mitochondrial pathway of apoptosis. Interestingly, both p-AKT and total AKT, but not p-ERK or total ERK, were dramatically upregulated in BAKR cells, correlating with high levels of CD133 expression in these cells (Figure 1F) and indicating activation of an AKT survival pathway associated with increased cell survival and drug resistance in CD133-overexpressing BAKR cells. CD133 overexpression in BAKR cells also correlates with the dramatic upregulation of basal levels of anti-apoptotic BCL-2 and down-regulation of the pro-apoptotic BAX protein which may tilt the balance towards increased cell survival upon treatment with targeted melanoma therapeutics.

### 2.2. CRISPR-Cas9 Knockout of CD133 in Conditionally Reprogrammed BAKR Cells Increases Apoptotic Caspase-3 Activation in Response to Trametinib via Bcl2 Downregulation

To determine whether CD133 expression in BAKR is merely correlated with or essential for increased cell survival after trametinib, CD133 knockout was achieved by targeting three CD133 coding regions in exon 1 using CRISPR-Cas9 expressed in a lentiviral vector pLenti-U6-sgRNA-SFFV-Cas9-2A-Puro (Addgene) as previously described [58]. Different single guide RNAs (sgRNAs; Sc, T1, T2, and T3) were used to target each exon 1 site by transduction with lentivirus expressing Cas9 together with sgRNA for the 3 targets (T1, T2, and T3) or with control SC sgRNA, followed by selection with puromycin. Due to more frameshift mutations in T3 compared to T1 and T2 cell lines, T3 cells (BAKR-KO) showed effective knockout of the CD133 protein and RNA [58] and were therefore used in subsequent experiments in this study. 

**Figure 2:**
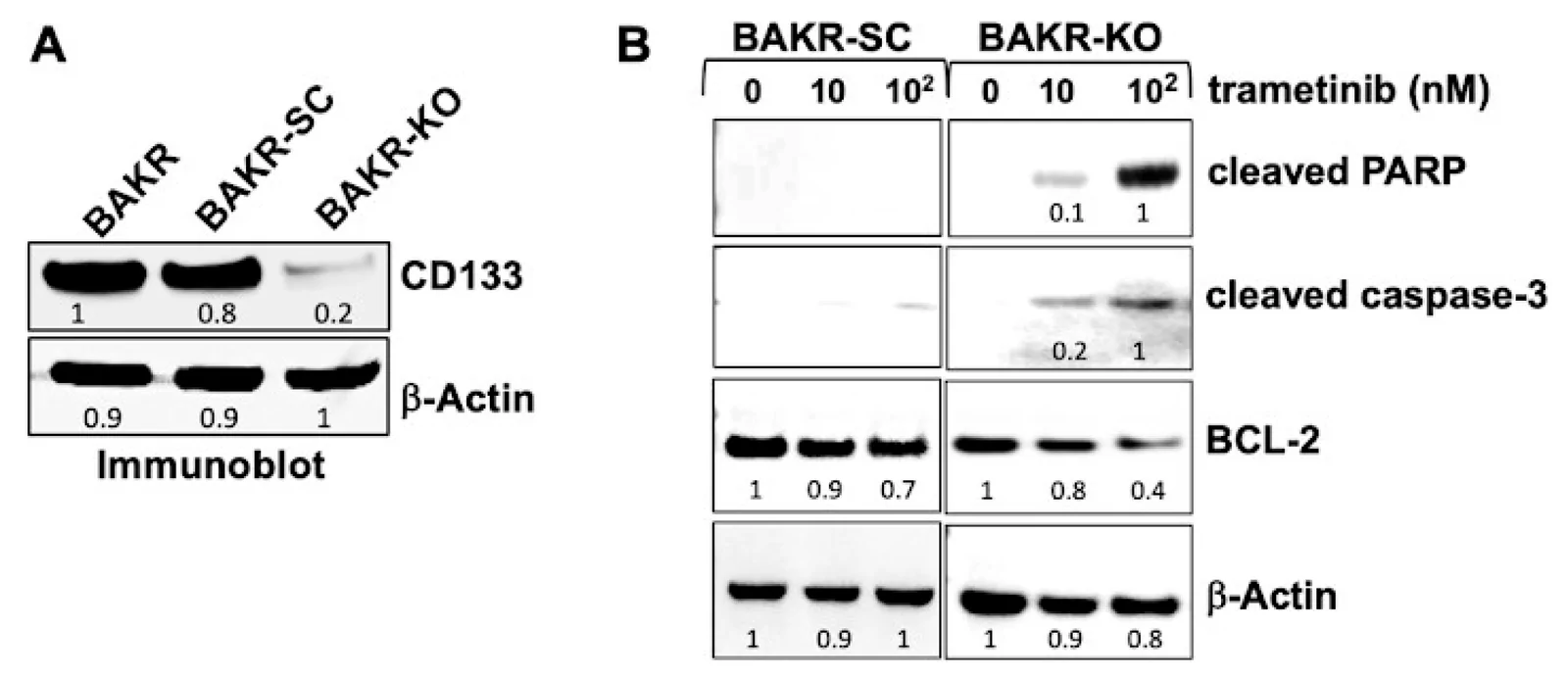
CRISPR-Cas9 CD133-knockout sensitizes BAKR-KO cells to trametinib-induced apoptosis via reduced BCL-2 levels. CD133 expression was compared between BAKR, CD133-knockout BAKR-KO, and sgRNA control BAKR-SC cells. (**A**) Immunoblot analysis confirms depletion of CD133 protein in BAKR-KO, compared to BAKR-SC and BAKR cells. (**B**) BAKR-SC and BAKR-KO cells were exposed to increasing trametinib concentrations for 72 h and subjected to immunoblot analysis with antibodies to the active cleaved form of caspase-3 (p17), cleaved PARP, BCL-2, and β-Actin for loading control. Densitometric analysis comparing intensities of protein bands relative to bands with the highest intensities is shown in immunoblots.

RT-PCR with primers flanking the sgRNA target [58] and immunoblot analysis (Figure 2A) verified depletion of CD133 protein in BAKR-KO cells, compared to BAKR or scrambled sgRNA controls (BAKR-SC), after CRISPR-Cas9-associated sgRNAs targeting of exon 1 of the *CD133* gene. CD133 knockout in BAKR cells effectively reduced CD133 protein levels by 80% (Figure 2A). To compare the drug sensitivities of BAKR cells expressing the *CD133* gene (BAKR-SC) and CD133-depleted BAKR-KO, cells were exposed to increasing doses of trametinib and subjected to immunoblot analysis for apoptosis markers (caspase-3 proteolytic activation and PARP cleavage; Figure 2B).

### 2.3. CRISPR-Cas9 Knockout of CD133 Expression in BAKP Melanoma Cells Increases Caspase 3-Mediated Apoptosis in Response to Trametinib

CD133-associated drug resistance was next investigated in BAKP cells, the parental cells from which the CD133-overexpressing conditionally reprogrammed BAKR cells were derived. Similar to BAKR, lentiviral delivery of CRISPR-Cas9 associated sgRNAs targeting three loci (T1, T2, and T3) in exon 1 of the *PROM1* gene effectively knocked out CD133 expression in the T3 cells (BAKP-KO) because mutations generated in these cells were mostly frameshift mutations, whereas most mutations in T1 and T2 cells were in-frame [58]. 

**Figure 3:**
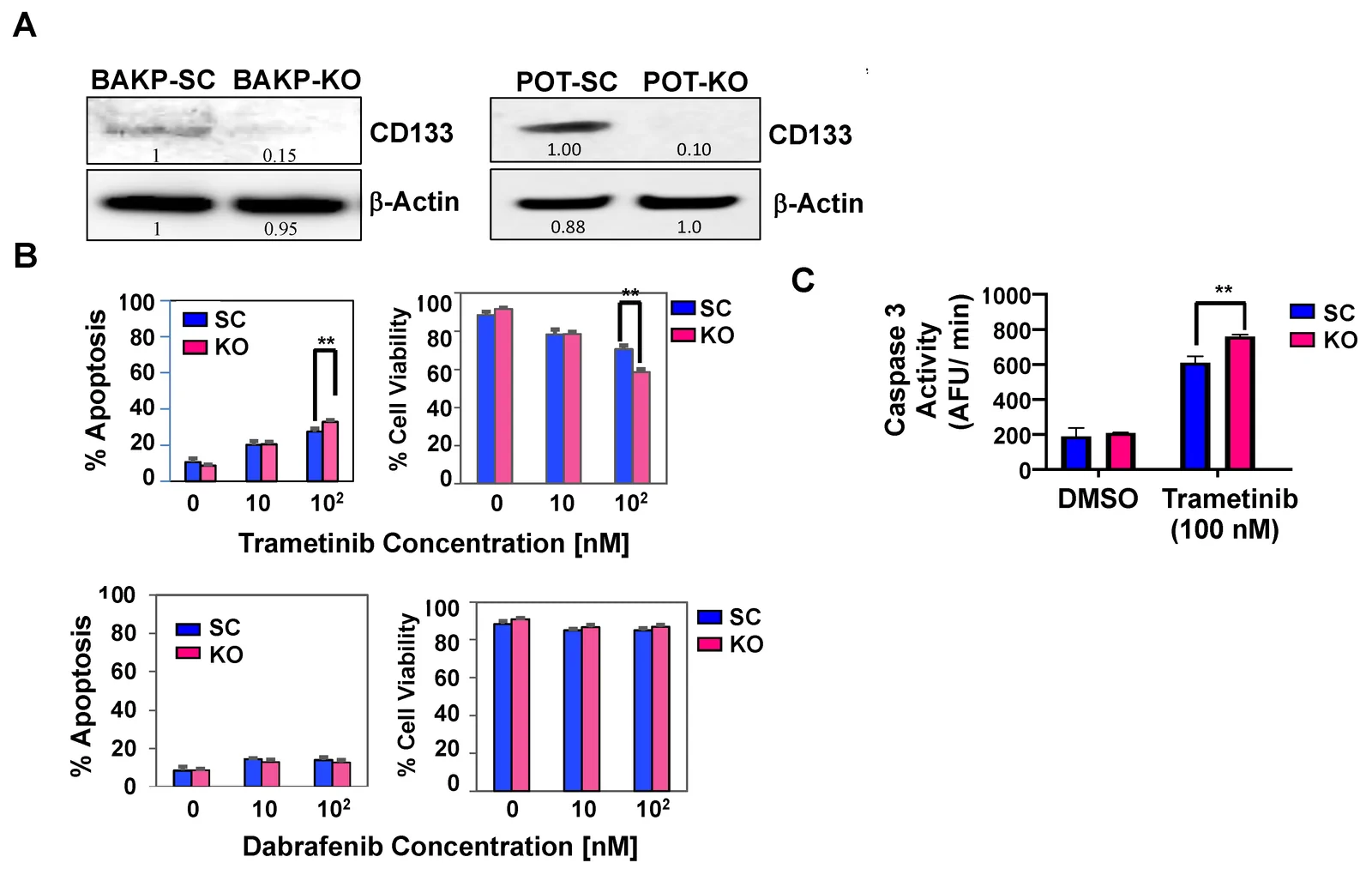
CRISPR-Cas9 knockout of CD133 expression in parental BAKP melanoma cells (**A**) increases caspase-3 mediated apoptosis (**B**,**C**) after exposure to trametinib. (**A**) Immunoblot analysis with anti-CD133 in BAKP-SC vs. BAKP-KO cells (left panel) and POT SC vs. POT-KO cells (right panel). (**B**) BAKP-SC and BAKP-KO cells were treated with indicated concentrations of trametinib (upper panels) or dabrafenib (lower panels) and subjected to Annexin FITC/PI apoptosis assays. Trametinib-treated CD133-depleted BAKP-KO cells display enhanced apoptosis relative to control BAKP-SC cells, an effect that was not seen with dabrafenib. (**C**) Fluorometric caspase-3 activity assays were performed 72 h after exposure of BAKP-SC and BAKP-KO cells to 100 nM trametinib. Results shown are the means ± SD of three biological replicates of a representative experiment; essentially the same results were obtained in three independent experiments. ** represents *p* < 0.01.

As with BAKR-KO, immunoblot analysis verified an 85% reduction in immunodetectable CD133 protein after CRISPR-Cas9 knockout of CD133 expression in BAKP-KO, compared to control BAKP-SC cells (Figure 3A, left). These cells were therefore used for subsequent experiments. POT, another drug-resistant patient-derived primary melanoma cell line that harbors an NRAS^Q61R^ driver mutation, was also used to validate the effects of CD133 knockout by CRISPR-Cas9. As with BAKP-KO cells, POT-KO cells were derived, exhibiting a 90% reduction in CD133 expression compared to POT-SC control cells (Figure 3A, right). Annexin-FITC/PI flow cytometry analysis (Figure 3B, upper panels) and fluorometric caspase-3 activity assays (Figure 3C) after trametinib treatment reveal significantly elevated caspase 3- mediated apoptosis and decreased cell viability in BAKP-KO cells, indicating that CD133 depletion sensitizes cells to trametinib. As expected, BAKP cells were resistant to the BRAFi dabrafenib since these cells lack the BRAFV600E mutation targeted by this drug (Figure 3B, lower panels). 

### 2.4. CRISPR-Cas9 Knockout of CD133 in BAKP and POT Cells Enhances Trametinib-Induced Apoptosis via Downregulation of Pro-Survival BCL-2 Family Members and AKT

**Figure 4:**
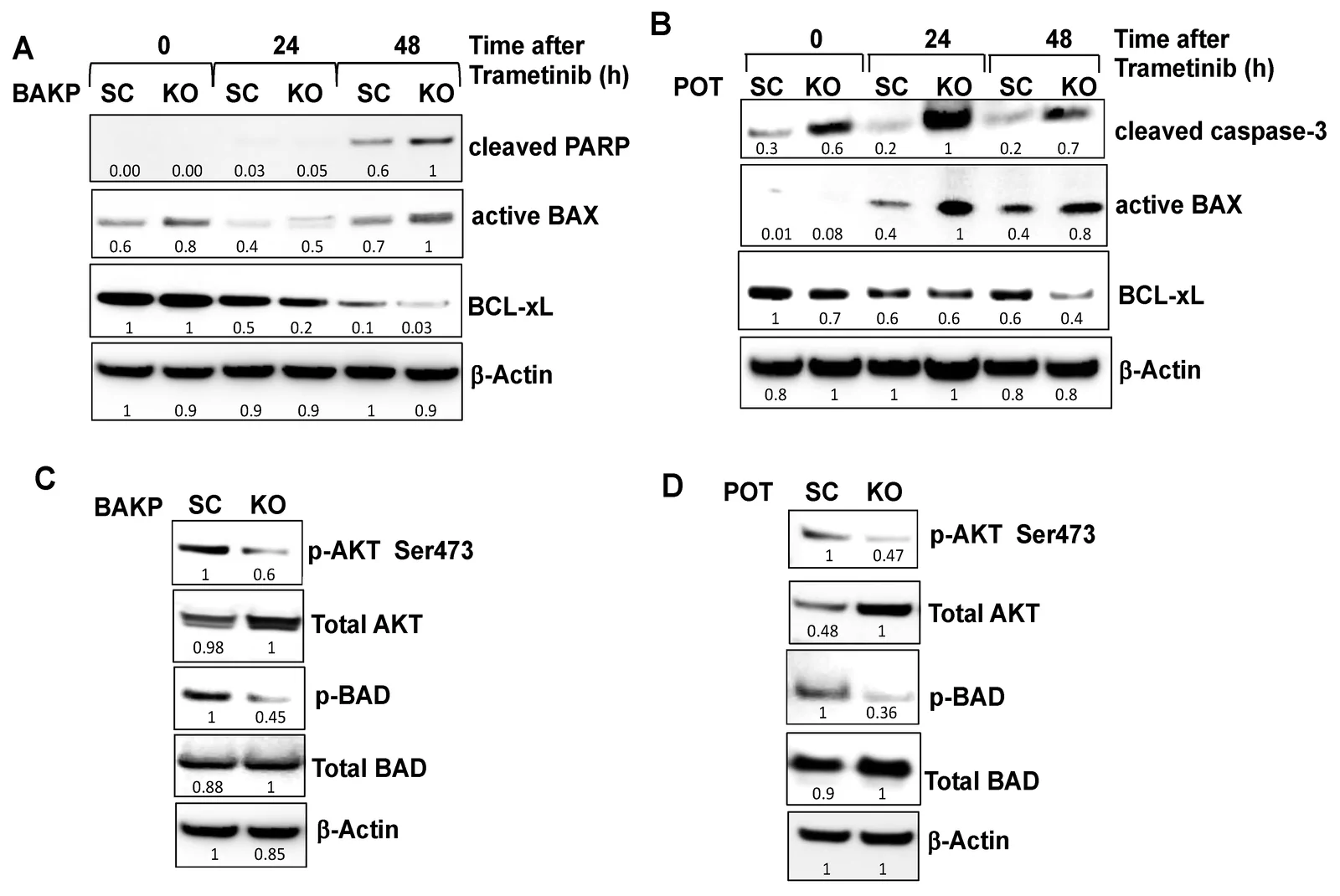
CRISPR-Cas9-mediated knockout of CD133 expression in BAKP (**A**) and POT (**B**) cells increases BAX activation, and PARP cleavage following trametinib treatment, sensitized by reduced basal levels of pro-survival BCL-xL, pAKT, and pBAD. (**A**) BAKP-KO, BAKP-SC cells, as well as (**B**) POT-SC and POT-KO cells were treated with 100 nM trametinib for 24 and 48 h, followed by immunoblot analysis with antibodies to cleaved PARP, cleaved active form of caspase-3, active BAX, BCL-xL, and β-actin as loading control. CD133 knockout in trametinib-exposed both BAKP-KO (**A**) and POT-KO (**B**) cells increases pro-apoptotic active BAX and decreases anti-apoptotic BCL-xL levels (**A**,**B**), sensitizing cells to apoptosis. Immunoblot analysis of pAKT, AKT, p-BAD, Bad, and β-actin as loading control in BAKP-KO (**C**) and POT-KO cells (**D**), compared to the SC controls. Densitometric analysis comparing intensities of protein bands relative to bands with the highest intensity is shown in immunoblots.

To examine the potential mechanism(s) by which CD133 modulates the apoptotic machinery to promote cell survival and suppress trametinib-induced apoptosis, CD133-depleted CRISPR-Cas9 knockout BAKP-KO and control BAKP-SC cells were treated with 100 nM trametinib for 24 and 48 h, followed by immunoblot analysis with antibodies to apoptotic proteins. Consistent with the dose-response experiments, BAKP-KO exposed to 100 nM trametinib exhibit a 40% increase in PARP cleavage at 48 h compared to control BAKP-SC cells (Figure 4A). Likewise, a robust increase in the proteolytic activation of caspase 3 after exposure to 100 nM trametinib was noted in CD133-depleted POT-KO but not in POT-SC control cells (Figure 4B). Compared with control POT-SC cells, POT-KO cells showed a three- to five-fold higher proteolytic activation of caspase-3 after trametinib treatment (Figure 4B). Sensitization to trametinib apoptosis by CD133 knockout was therefore verified in both BAKP and POT melanoma cells. 

Further, CD133-depleted BAKP-KO and POT-KO cells exhibit a ~2-fold increase in the active form of pro-apoptotic BAX (Figure 4A,B). Upon apoptosis induction, BAX undergoes a conformational change to its active form and translocates from the cytosol to the mitochondria where it dimerizes with other BAX and/or BAK molecules to form oligomeric pores in the mitochondrial outer membrane, resulting in the release of cytochrome c and other pro-apoptotic factors, leading to caspase activation. Whereas levels of the active form of BAX increased, CD133 knockout in both BAKP-KO and POT-KO cells caused a 50% reduction in expression of anti-apoptotic members of the BCL-2 family such as BCL-xL (B-cell lymphoma-extra-large) compared to control cells after trametinib treatment. BCL-xL heterodimerizes with and inhibits the activity of BAX, thus, the increase in BAX-triggered apoptosis can at least in part be attributable to lower BCL-xL protein levels resulting from CD133 knockout.

A decreased apoptotic response to trametinib in CD133-expressing cells may be due to higher levels of activated Protein kinase B (AKT) which phosphorylates BCL-2 associated agonist of cell death (BAD), converting it to its pro-survival form (p-BAD); pBAD is unable to bind and antagonize anti-apoptotic BCL-2 and BCL-xL, leaving them free to bind BAX, thereby inhibiting BAX-mediated apoptosis. We next examined whether increased cell survival and resistance to trametinib of CD133(+) melanoma stem cells are mediated by AKT activation via upregulation of AKT expression or phosphorylation at serine 473 (S473). Immunoblot analysis with antibodies to AKT and p-AKT (Ser473) revealed that AKT activation, as indicated by phospho-AKT (p-AKT), in CD133-depleted BAKP-KO cells was 40% lower than in control BAKP-SC cells (Figure 4C). Likewise, CD133-depleted POT-KO cells exhibited a marked reduction in p-AKT levels compared with control cells, sensitizing them to trametinib (Figure 4D). Consistent with reduced levels of pAKT, pBAD was also 50% lower in both CD133-depleted BAKP-KO (Figure 4C) and POT-KO cells (Figure 4D), compared to their respective SC controls. Thus, CD133 may suppress trametinib-induced apoptosis by enhancing AKT-mediated BAD phosphorylation and elevating BCL-2 and BCL-xL. Conversely, reduced AKT activation (pAKT) in CD133 knockout cells, increases dephosphorylated BAD, inactivating BCL-2 and BCL-xL, preventing them from binding and inhibiting BAX, and promoting BAX-initiated apoptosis.

### 2.5. Dox-Inducible Expression of CD133 Has Opposite Effects of CRISPR-cas9 CD133 Knockout, Suppressing Trametinib-Induced Apoptosis via Modulation of BCL-2 Family Members Mediated by AKT in BAKP and POT Cells 

**Figure 5:**
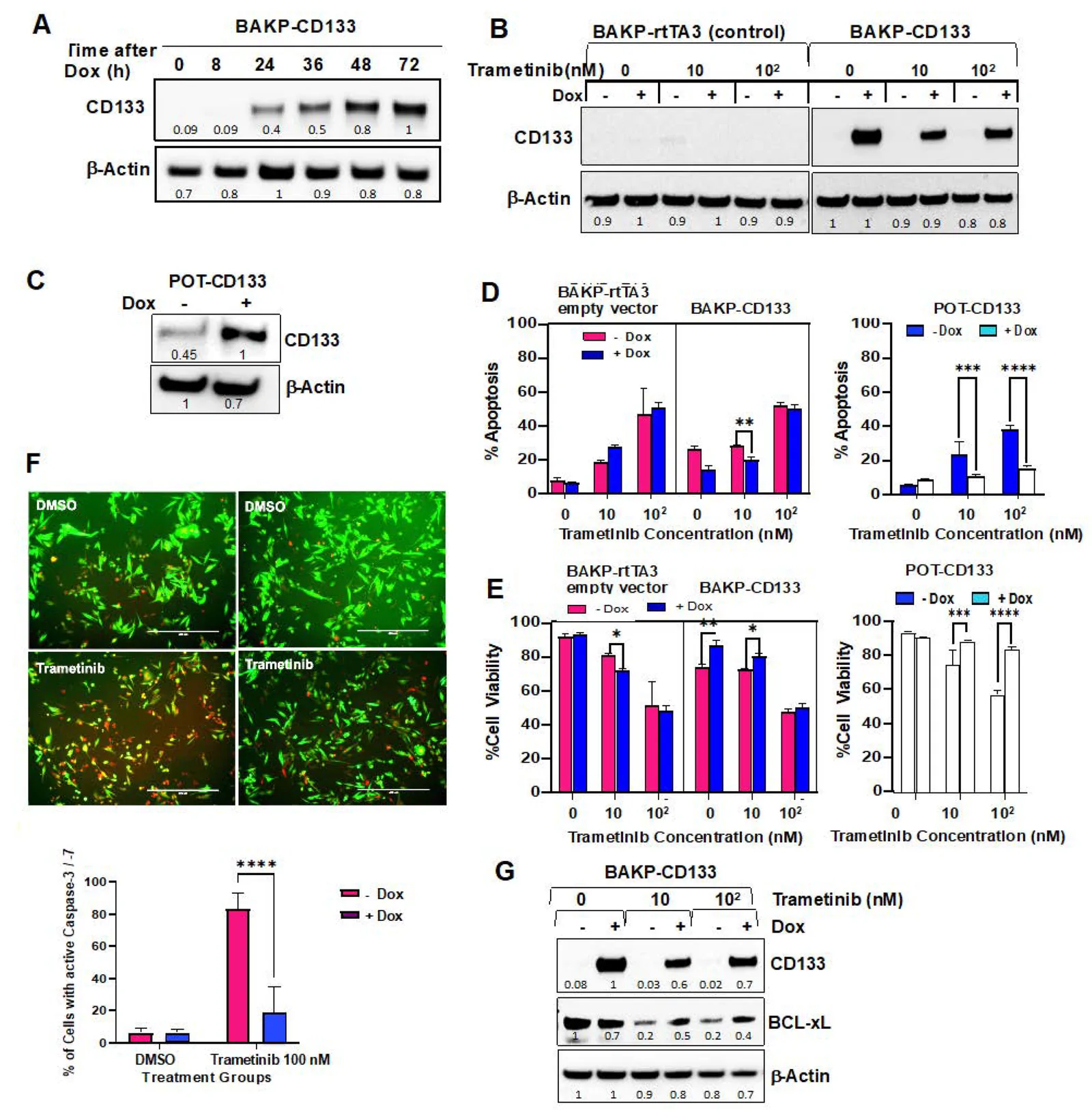
Dox-induced CD133 expression in BAKP (**A**,**B**) and POT (**C**) cells, as verified by immunoblot analysis, suppress caspase-3 mediated apoptotic cell death (**D**,**F**) and increases cell viability (**E**) after trametinib treatment (dose-response experiments). (**A**) Cells were incubated with Dox for the indicated times and subjected to immunoblot analysis with antibodies to CD133 and β-actin for normalization. (**B**) Immunoblot analyses with anti-CD133 of BAKP-CD133 and vector control BAKP-rtTA3 cells treated with increasing trametinib doses +/− Dox, as well as (**C**) POT-CD133 cells incubated +/− Dox for 72 h. (**D**) BAKP-CD133, BAKP-rtTA3 empty vector control, and POT-CD133 cells were exposed to increasing doses of trametinib for 72 h, and subjected to Annexin-FITC/PI apoptosis assays; apoptosis (%; **D**) and cell viability (%; **E**) of cells are shown. (**F**) Fluorescent FLICA Caspase 3/7 activity assays reveal a significant decline in caspase 3 activity in trametinib-treated CD133-expressing BAKP cells (+Dox), compared to uninduced cells (−Dox); representative merged images of red fluorescent FLICA- and GFP-positive cells (10×) (left panel); quantification of FLICA-positive cells (with active caspase-3, right panel). (**G**) Immunoblot analyses with antibodies to CD133, BCL-xL, and βActin of BAKP-CD133 treated with increasing trametinib doses +/− Dox. BAKP-CD133 cells were constitutively expressing GFP. Results shown are the means ± SD of three biological replicates of a representative experiment of three independent experiments. *, **, ***, **** represent *p* < 0.05, *p* < 0.01, *p* < 0.001, and *p* < 0.0001, respectively. Densitometric analysis comparing intensities of protein bands relative to bands with the highest intensity is shown in immunoblots.

To further confirm the mechanistic pathway between CD133, AKT activation, and the pro- and anti-apoptotic BCL-2 family proteins, BAKP, and POT melanoma cells expressing low barely detectable CD133 levels, were co-transduced with a Tet activator (rtTA3) plus a Tet-on vector expressing CD133 (TRE3G-CD133), or with rtTA3 alone, and selected with blasticidin and gentamicin. Stable pooled clones of BAKP-rTTA3-TRE3G-CD133 (BAKP-CD133) were then incubated with or without 1 µg/mL doxycycline (Dox) for the indicated times. Immunoblot analysis with anti-CD133 showed a marked time-dependent increase in CD133 expression by 24 h in the presence of Dox, with CD133 levels remaining elevated for at least 72 h (Figure 5A). BAKP-CD133 and BAKP- rtTA3 empty vector control cells were then exposed to increasing doses of trametinib +/− Dox for 72 h. As expected, BAKP-CD133, but not control BAKP- rtTA3, exhibited a robust induction of CD133 expression with Dox, retaining expression even at the highest dose of trametinib tested (Figure 5B). Likewise, POT- rTTA3-TRE3G-CD133 (POT-CD133) cells, a different melanoma cell line that expresses higher basal CD133 levels than BAKP cells, showed a two-fold increase in CD133 expression after Dox induction (Figure 5C). 

In dose-response experiments, Dox-inducible BAKP and POT cell lines, incubated with increasing concentrations of trametinib for 72 h with or without Dox, were subjected to Annexin-FITC/PI apoptosis assays. While trametinib induced a dose-dependent increase in apoptosis in all cell lines, apoptosis was significantly attenuated in the presence of Dox (Figure 5D), coincident with increased cell viability (Figure 5E) in BAKP-CD133 cells after treatment with 10 nM, but not with 100 nM trametinib. Dox-treated POT-CD133 exhibited reduced apoptosis and increased cell survival after exposure to 10 or 100 nM trametinib. The differential response of BAKP-CD133 compared to POT-CD133 cells may be attributable to higher basal expression levels of CD133 in POT compared to BAKP cells, consistent with diminished apoptosis/increased cell viability in POT cells. In contrast, there was no difference in apoptosis and cell viability in control empty vector BAK-P-rtTA3 cells in the presence or absence of Dox, confirming that the decreased apoptosis/ increased cell viability was not due to Dox itself, but due to induced expression of CD133. Consistently, FLICA caspase activity assays, followed by fluorescent imaging and quantification, revealed a significant decline in caspase 3 activity in Dox+ CD133-expressing BAKP cells exposed to 100 nM trametinib, compared to uninduced cells (Figure 5F). Additionally, anti-apoptotic BCL-xL decreased with trametinib treatment, a response that was reversed by Dox-induced CD133 expression in BAKP cells. (Figure 5G). 

**Figure 6:**
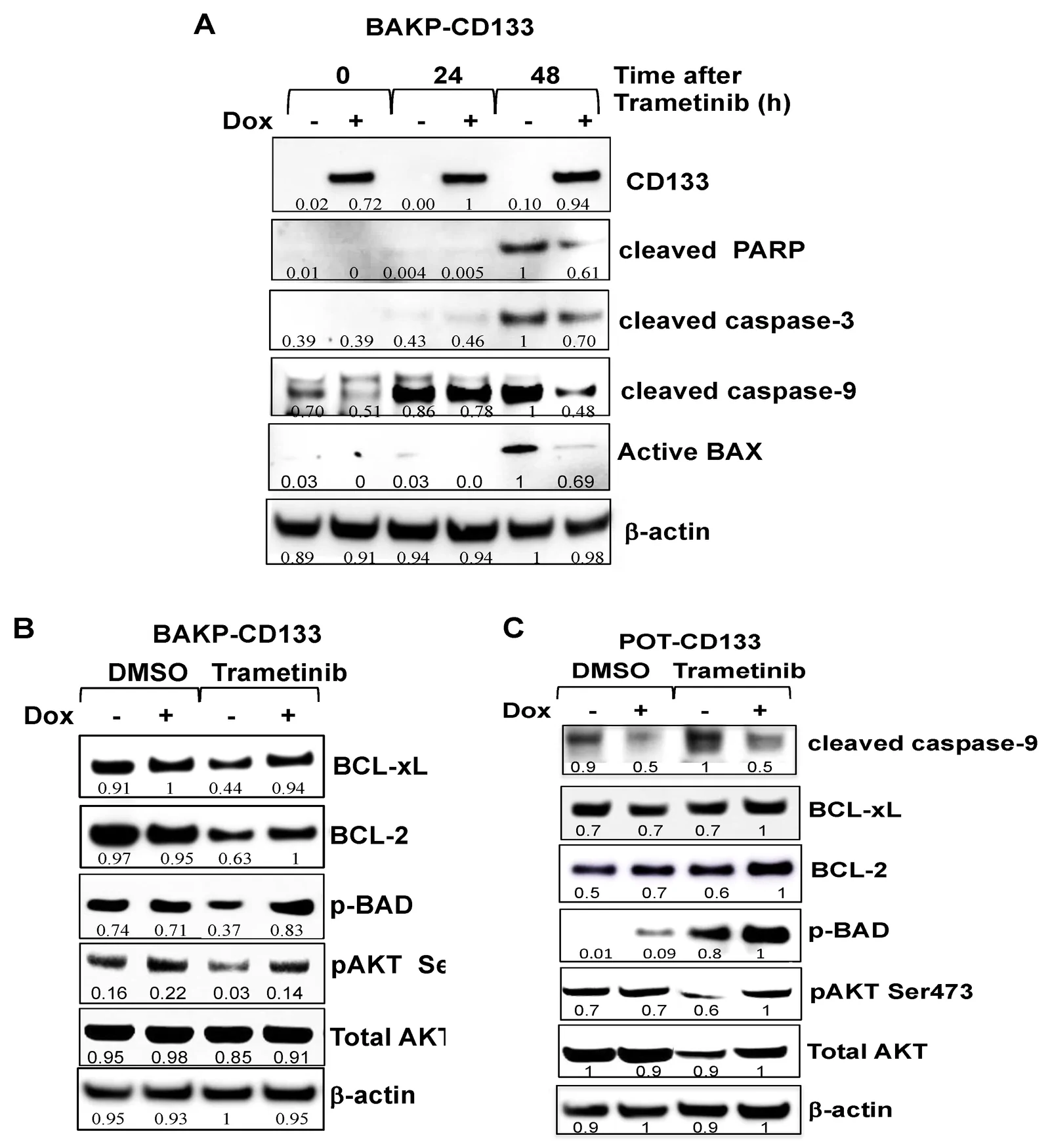
Dox-inducible CD133 expression attenuates trametinib-induced apoptosis in BAKP-CD133 (**A**,**B**) and POT-CD133 cells (**C**), as evidenced by reduced PARP cleavage, proteolytic activation of caspases-3 and -9, and activation of BAX (**A**), and upregulation of anti-apoptotic proteins BCL-xL, BCL-2, p-BAD, and p-AKT (**B**), in response to trametinib treatment. (**A**) BAKP-CD133 cells were exposed to 100 nM trametinib for 24 or 48 h +/− Dox, followed by immunoblot analysis with antibodies to apoptotic markers cleaved PARP, cleaved caspase-3, cleaved caspase-9, and active Bax. BAKP-CD133 (**B**) and POT-CD133 (**C**) treated with 100 nM trametinib +/− Dox were subjected to immunoblot analysis with anti-apoptotic proteins BCL-xL, BCL-2, p-BAD, p-AKT, AKT, and β-actin for normalization. Densitometric analysis comparing intensities of protein bands relative to bands with the highest intensity is shown below each band in immunoblots.

Dox-inducible cell lines were next used in time-course experiments. Cells were treated with or without Dox to induce CD133 expression and exposed to 100 nM trametinib for 24 or 48 h to induce apoptosis. Immunoblot analysis at indicated time points after drug treatment assessed differences in expression levels of the apoptotic markers cleaved PARP and cleaved caspases- 3 and 9, pro-apoptotic or anti-apoptotic members of the BCL-2 family proteins BAX, BAD and p-BAD, BCL-2, and BCL-xL, as well as AKT and its activated form (p-AKT) (Figure 6A,B). Caspase 3-mediated cleavage of PARP to its 89 kDa cleaved form was detected by 48 h in trametinib-treated BAKP-CD133 cells, concomitant with robust proteolytic activation of caspase-3, as shown by an increase in its cleaved active form (Figure 6A). Caspase-9, the initiator caspase that cleaves and activates caspase-3 in the intrinsic pathway of apoptosis, is itself cleaved and activated in response to trametinib, showing an increase in the cleaved form by 24 h. Consistent with the dose-response experiments, proteolytically-cleaved active forms of caspases-3 and -9, as well as cleaved PARP, were clearly diminished in Dox-treated CD133-expressing cells, exhibiting 30–50% reduction in levels, compared to cells without Dox, further verifying suppression of trametinib-induced caspase-3 mediated apoptosis by CD133 (Figure 6A). 

Activation of caspase-9 requires BAX activation which allows the release of cytochrome c and other pro-apoptotic molecules (e.g., SMAC/DIABLO) from the mitochondria that facilitate the formation of active apoptosomes needed for caspase-9 activation. Not surprisingly, therefore, trametinib induces a 95% increase in the levels of active BAX by 48 h, which is inhibited by Dox-induction of CD133 expression, as a 40% reduction of the active form of BAX is noted in cells incubated with Dox, compared to those without Dox (Figure 6A). Consistently, levels of anti-apoptotic/pro-survival BCL-2 family members such as BCL-2 and BCL-xL markedly increased after trametinib treatment (Figure 6B). Similarly, phosphorylation of BAD, which converts the pro-apoptotic BAD to a pro-survival p-BAD protein, as well as AKT, a pro-survival kinase that is activated by phosphorylation, were also substantially enhanced in BAKP-CD133 cells after Dox induction of CD133 expression. These results together suggest that CD133 plays a key role in apoptosis suppression and resistance to trametinib by activating an AKT/phospho-BAD survival pathway in human melanoma cells.

To further validate the effects of increased CD133 expression on trametinib-induced apoptosis, AKT activation, and levels or phosphorylation of pro- and anti-apoptotic BCL-2 family proteins, we compared the consequences of induced CD133 expression in another Dox-inducible melanoma cell line, POT-CD133. Similar to BAKP cells, Dox-inducible CD133 expression in POT cells suppressed trametinib-induced apoptosis, as indicated by decreased proteolytic activation of caspase-9, increasing cell survival through upregulation of anti-apoptotic proteins BCL-2 and BCL-xL, and increased phosphorylation of BAD to its pro-survival form p-BAD (Figure 6C). Moreover, increased phosphorylation and activation of AKT after trametinib treatment was also verified in POT-CD33 cells in the presence of Dox after trametinib treatment. Attenuation of trametinib-induced apoptosis in both Dox-treated CD133-expressing BAKP-CD3133 as well as POT-CD133 cells compared to cells without Dox, and the reproducible effects of CD133 expression on BCL-2 family members and AKT activation in different melanoma cell lines, indicate that these responses are not cell line-specific and support a role for CD133 in apoptosis suppression and increased cell survival after trametinib in different melanoma lines.

### 2.6. Targeting Nodes of the CD133, AKT, and BCL-2 Family Survival Pathway, and/or Trametini, Reveals the Potential for Combination Therapies for NRAS Mutant Melanoma

**Figure 7:**
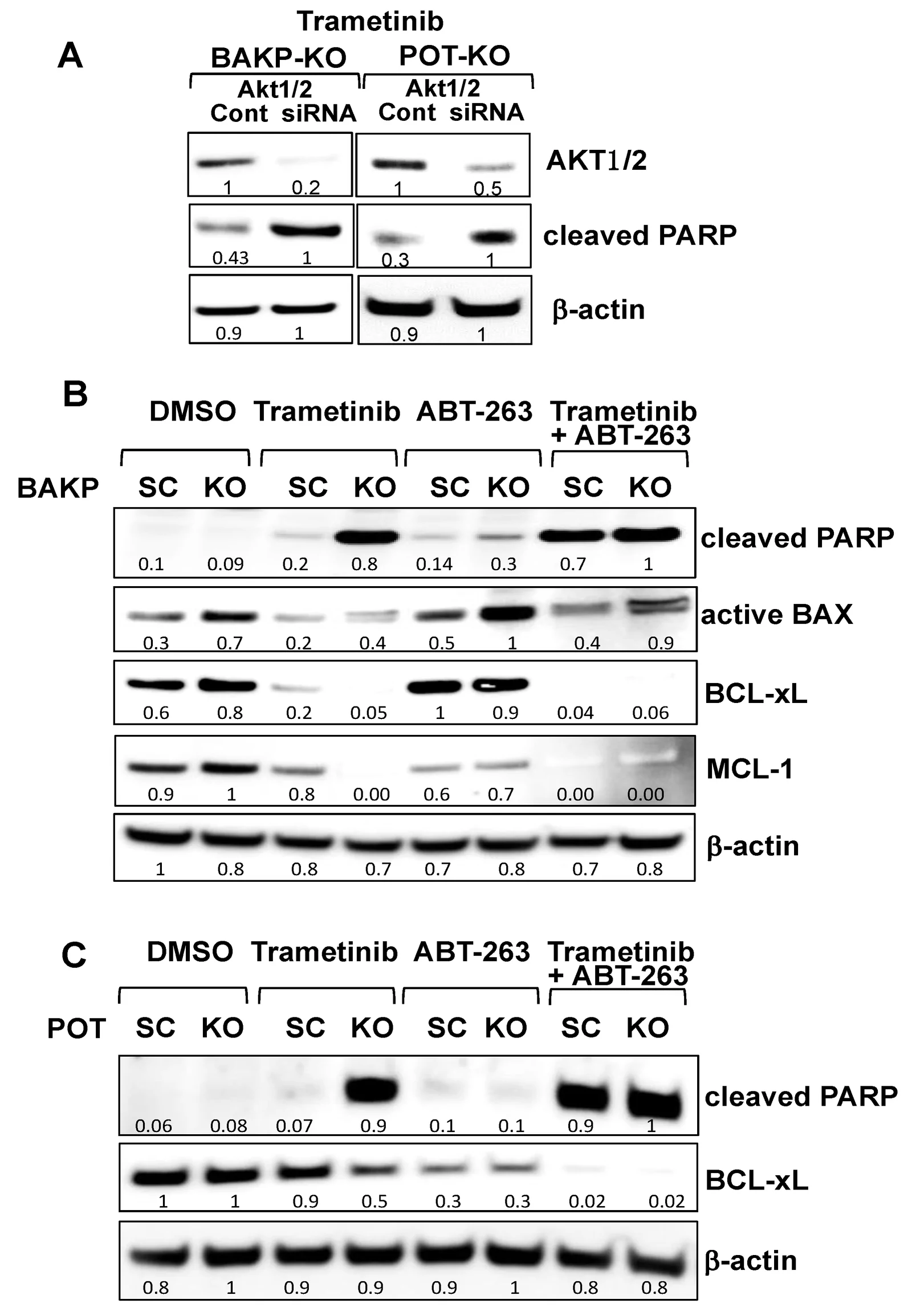
siRNA knockdown of AKT1/2 in BAKP (**A**, left panel) and POT cells (**A**, right panel), or BCL-2 family inhibitor ABT-263 and/or CD133 knockdown reveals that AKT1/2 siRNA knockdown or BCL-2 family inhibition with ABT-263 plus CD133 knockdown both block the AKT pathway, inducing maximum apoptosis in the presence of trametinib in BAKP (**B**) and POT cells (**C**). (**A**) BAKP-KO and POT-KO cells were transfected with AKT1/2 siRNA pool or the scrambled control siRNA (“Cont”). Then, 48 h after transfection, cells were treated with 100 mM trametinib for 24 h, and subjected to immunoblot analysis with antibodies to AKT1, cleaved PARP, or β-actin. (**B**) BAKP-KO and (**C**) POT-KO cells or their respective controls BAKP-SC and POT-SC cells were treated for 48 h with trametinib (100 nM) or the BCL-2 family inhibitor ABT-263 (100 nM) alone or in combination, and then subjected to immunoblot analysis with antibodies to cleaved PARP, active BAX, BCL-xL, MCL-1, or β-actin. Densitometric analysis comparing intensities of protein bands relative to bands with the highest intensity is shown in immunoblots.

We further delineated the molecular mechanism(s) of CD133-associated inhibition of apoptosis using CRISPR-cas9 knockout BAKP-KO and POT-KO cells or their respective controls, BAKP-SC and POT-SC cells. CD133 binds to and activates phosphoinositide 3-kinase (PI3K) [60], resulting in preferential activation of AKT, which can then phosphorylate and inactivate pro-apoptotic BAD. To determine if the suppressive effects of CD133 on apoptosis are mediated through AKT activation, followed by BAD phosphorylation, or by upregulation of anti-apoptotic BCL-2 family members, we modulated AKT1/2 expression and activity by AKT siRNA knockdown (Figure 7A) or inhibited anti-apoptotic BCL-2 family members with ABT-263 (Figure 7B). 

Immunoblot analysis confirmed successful partial AKT1/2 knockdown by siRNA in BAKP-KO and POT-KO cells compared to their control SC control cells (Figure 7A). CD133 knockout in combination with AKT siRNA knockdown further enhanced trametinib-induced apoptosis, as indicated by increased PARP cleavage in both BAKP-KO and POT-KO cells. 

To further investigate the interaction between CD133 and anti-apoptotic BCL-2 family members, BAKP-KO and BAKP-SC cells were treated with 100 nM trametinib or the BCL-xL, BCL-2, and BCL-w inhibitor ABT-263/navitoclax alone, or in combination (Figure 7B,C). ABT-263 mirrored the effects of CD133 knockout in both BAKP and POT cells since near-maximal apoptosis, as assessed by PARP cleavage, was only induced by either a combination of CD133 knockout plus trametinib or BCL-2 family inhibition by ABT-263 plus trametinib. BAKP-KO cells also increased levels of the active form of BAX after BCL-2 family inhibition with ABT-263. In the presence of ABT-263, levels of cleaved-PARP and active BAX in BAKP-SC cells increased almost to the same extent as that exhibited by CD133-knockout in BAKP-KO cells. Interestingly, combinations that repressed BCL-xL and MCL-1 coincided with PARP cleavage. Thus, trametinib in combination with either AKT1/2 knockdown or BCL-2 family inhibition mimics CD133 knockout. These combined results indicate that CD133-positive MIC may be treated with combinations of MEKi and CD133 pathway inhibitors.

## 3. Discussion

Previous studies highlighted a role for CD133 in AKT activation in liver and gastric cancer, as well as gliomas [60,61,62], where phosphorylation of CD133 Y828 facilitates its binding to the p85 subunit of phosphoinositide 3-kinase (PI3K), thereby phosphorylating and activating AKT [60]. Our current study is consistent with these previous studies in that we also see a CD133-AKT activation pathway. The uniqueness of our study is that we have (1) extended these findings to melanoma, (2) utilized CRISPR-Cas9 vs. CD133 siRNAs employed in previous studies, (3) used an inducible CD133 expression system, (4) examined downstream apoptotic events (caspases and their substrates), (5) utilized pharmacological inhibitors (BCL-2) and siRNAs (AKT), along with altered CD133 expression to determine the feasibility of combinatorial therapies for melanoma, and (6) examined the response to both chemotherapeutic agents (DTIC) and kinase inhibitors (trametinib). In the current study, we utilized different patient-derived melanoma cell lines (BAKP, BAKR, and POT) with CRISPR/Cas9-mediated CD133 KO, as well as Dox-inducible CD133 expression in two different cell lines (BAKP and POT) and showed that CD133 is part of a CD133/AKT/BAD-dependent pathway, working in combination with a previously characterized RAS/RAF/MEK/ERK pathway [63]. Together, these pathways inhibit BAX/caspase-9-mediated apoptosis, including increased activation of caspase-3, PARP cleavage, and DNA fragmentation (sub-G1). Importantly, inhibition of both CD133 and MAPK pathways dramatically enhances apoptosis. These two pathways may be working in concert with CD133-upregulated ABCBG2 to confer drug resistance [57]. 

Five percent of BAKP cells are CD133+, increasing to 85% after MACS sorting, but most cells lose expression of CD133 within 2 weeks [57,58]. Conditional reprogramming stabilizes CD133 levels and inhibits apoptosis, although the relationship was previously only correlative [58]. We, therefore, knocked out or expressed CD133 in the present study. CD133 knockout in CD133-overexpressing BAKR cells increased multiple markers of apoptosis, including cleavage of caspase-3 and PARP, DNA fragmentation (sub G1), and translocation of phosphatidylserine to the outer leaflet of the cell membrane, as measured by the binding of Annexin V-FITC. To confirm that the role of CD133 in increasing cell survival in response to trametinib is not merely attributable to other changes associated with reprogramming in BAKR cells, BAKP and POT parental cells were also depleted of CD133 by CRISPR-Cas9 which consistently reduced phosphorylation/ activation of AKT and BAD, diminished BCL-XL, and increased activated BAX and caspase-3, PARP cleavage, as well as % apoptosis in cells. 

Although single-cell clones of CRISPR-Cas9 CD133 KO melanoma cells were derived after transduction with lentivirus expressing Cas9 along with sgRNA for each of the three targets (T1, T2, and T3), followed by selection with puromycin, isolated clones of CD133+ cells lost CD133 expression over time, presumably due to the number of population doublings required to establish a clonal population; thus, the loss of CD133 expression could not be ascribed to specific disruption of the CD133 gene. The experiment was therefore modified to isolate pools of CRISPR-Cas9 CD133 KO cells for each of the sgRNAs and these pooled clones were then sequenced by NGS to determine the sequences at each sgRNA target site containing substitutions and indels. The pooled clones of CD133 KO cells used in the current study represent a heterogenous pool of edited cells with different targeting events and varying indel populations, likely including cells with complete KO of CD133, as well as cells with limited or no CD133 knockout.

Conversely, Dox-induction of CD133 expression in both cell types rendered them resistant to the same markers of apoptosis. To clarify the roles of AKT and BCL-2, we used AKT siRNA or navitoclax, respectively, the latter of which is a pro-apoptotic BH3-mimetic [64] used in clinical trials for melanoma and other cancers, along with BRAF/MEK inhibition [65]. Our combined results suggest that two pathways are activated in drug resistance of NRAS mutant melanoma and that CD133 contributes to at least one of these pathways via AKT and the BCL-2 family, while the other pathway involves MAPK. These two pathways can be blocked by a combination of CD133/AKT/BCL-2 family inhibition along with suppression of the MAPK pathway. Thus, inhibition of the MAPK pathway with trametinib, in combination with inhibition of the AKT/BCL-2 pathway with either (1) AKT siRNA, (2) navitoclax, or (3) CD133 knockout can generate synthetic lethality and induce maximal apoptotic cell death. 

We showed that targeting CD133 by genetic manipulation helps to target MIC derived from different individuals. Several CSC CD133-targeted therapeutics in development include antibodies conjugated to a cytolethal distending toxin (CDT) capable of inducing DNA damage via its nuclease activity [66], several conjugated to pseudomonas exotoxin A, one linked to nanoparticles, and several bi- and tri-specific constructs. Anti-human mAb to CD133 conjugated to pseudomonas endotoxin [67] inhibited head and neck squamous cell carcinomas without interfering with hematopoietic lineages. Chimeric Antigen Receptor (CAR)-T cells specific for CD133 had initial success [62], as have NK cells targeting CD133 [66]. CD133-targeted aptamers can deliver nanoparticles to colorectal cancer cells [68] with significant growth inhibition. Our findings suggest different combinations of inhibitors that would be effective. For instance, given that BAKP-KO and POT-KO are sensitive to trametinib, MAPK inhibitors would be promising adjuvant to these CD133-based therapies; for example, mebendazole in combination with trametinib strongly suppresses MAPK of NRAS-mutant xenografts [63]. Other downstream suppressors of CD133/AKT/BCL-2 include AKT and BCL-2 family members, inhibited by siRNA and navitoclax, respectively, in the current study. Additional apoptotic gene pathways will be examined using our Dox-inducible BAKP-CD133 cells, including those regulated by TGFβ, which we have found to be important upon microarray and RNA-seq analysis of BAKR vs. BAKP cells, as well as DOX- vs. + BAKP cells (data not shown). We have also previously shown the importance of the TGFβ/ inhibitor of differentiation (ID) pathway in melanomagenesis [69,70]. Given that these gene products are regulated by miRs [71,72], the differential miR expression profiles in CD133(+) versus CD133(−) melanoma cells are also being further examined. 

## 4. Materials and Methods

### 4.1. Cells

Melanoma-initiating cells (MIC) were obtained from patient-derived human melanoma cell lines harboring different kinase mutations including BAKP (BRAFWT, NRASQ61K) and POT (BRAFWT, NRASQ61R). Cell suspensions, derived from fresh lymph node metastases from patients with short overall survival (<10 months), were mechanically dissociated by mincing in Iscove’s Modified Dulbecco’s Medium (IMDM) supplemented with FBS and antibiotics. Cells were assayed for MART1 and S100 by flow cytometry and maintained in IMDM/FBS/penicillin-streptomycin. BRAF or NRAS mutations were determined by Sanger sequencing as previously described [58]. 

### 4.2. Magnetic Sorting, Pre- and Post-Staining for CD133-Positivity and Conditional Reprogramming of CD133(+) Cells

CD133(+) and CD133(−) subpopulations were sorted on a MACS® Column and stained for CD133 (epitope 2; REA816; Miltenyi Biotech, Gaithersburg, MD, USA). CD133(+) cells were reprogrammed using the “Georgetown Method” [58]. Melanospheres were then moved to attached 2D conditions with DMEM-F12/FY stem cell media and cells were allowed to recover and expand at 37 °C in 5% CO2 for a month before CD133 immunofluorescence staining.

### 4.3. AKT Knockdown by siRNA

Knockdown experiments were performed according to standard protocols using small interfering RNA pools (siRNAs) specific for AKT1/2 or scrambled siRNA controls (Santa Cruz Biotech; sc-43609). Cells were transfected with siRNAs at 10 nM concentrations using Lipofectamine RNAiMAX (Invitrogen) and incubated for 48 h. Successful AKT1/2 knockdown was verified by immunoblot analysis.

### 4.4. CRISPR-Cas9 Deletion of CD133

Three different sgRNA sequences specific for CD133 exon 1 in lentiviral vector pLenti-U6-sgRNA-SFFV-Cas9-2A-Puro (Addgene, Watertown, MA, USA) were packaged and transduced into melanoma cells as previously described [58]. For lentiviral transduction and CRISPR-Cas9 KO, HEK 293 cells were transfected with a pLenti-U6-sgRNA-SFFV-Cas9-2A-Puro plasmid (4 µg) containing individual signal-guide sequences (sgRNA) beginning at either 8, 69, or 205 bp downstream of the beginning of the CD133 coding sequence, all within the first exon coding sequence of CD133, as shown below:

sgRNA1: CAACAGGGAGCCGAGTACGA (complement of Target 1 underlined below)
sgRNA2: TTCATCCACAGATGCTCCTA (complement of Target 2 underlined below)
sgRNA3: TTACCTTCTGGGAAATCACGC (complement of Target 3 underlined below)

The CD133 coding sequence of the first exon with targets T1, T2, and T3 highlighted:

**ATG** GCC CTC GTA CTC GGC TCC CTG TTG CTG CTG GGG CTG TGC GGG AAC **T1**
TCC TTT TCA GGA GGG CAG CCT TCA TCC ACA GAT GCT CCT AAG GCT TGG **T2**
AAT TAT GAA TTG CCT GCA ACA AAT TAT GAG ACC CAA GAC TCC CAT AAA GCT GGA CCC ATT GGC ATT CTC TTT GAA CTA GTG CAT ATC TTT CTT ATG TGG TAC AGC CGC GTG ATT TCC CAG AAG GTA A **T3**

Lentivirus packaging plasmids were transfected into HEK 293 cells using Lipofectamine LTX (ThermoFisher Sci, Waltham, MA, USA). Lentivirus was collected in media after 48 h and applied to patient-derived melanoma cells. After selection for 5 days with puromycin (4 µg/mL), pooled and individual clones were isolated and subjected to PCR analysis of genomic sequences. Alterations of exon 1 were assessed by PCR amplification followed by NGS sequencing to detect allelic or frameshift mutations (%) at CRISPR target sites T1, T2, and T3. 

To directly assess CRISPR-Cas9 deletions in pooled cells, the following primers flanking human genomic Targets 1, 2, or 3 (T1, T2, T3) were amplified as small PCR products where small NHEJ deletions could be visualized on 15% urea polyacrylamide gels.

Target 1-forward (F1a) TTCCCCAAGGCTTCCAGAAG
Target 1-reverse (R1a) GCCCTCCTGAAAAGGAGTTC
Target 2-forward (F2a) GAACTCCTTTTCAGGAGGGC
Target 2-reverse (R2a) GAGAATGCCAATGGGTCCAG
Target 3-forward (F3a) CTGGACCCATTGGCATTCTC
Target 3-reverse (R3a) CATTCT1TCCCTGCCATCAGC

### 4.5. Quantitative Reverse-Transcription PCR (qRT-PCR)

Total RNA was purified from cell pellets with Trizol Reagent (Gibco BRL, Grand Island, NY) and subjected to qRT–PCR by standard protocols using two-step reverse transcription–PCR (Invitrogen), RNA (0.75 μg), and specific primers listed below:

CD133 forward-5ʹ-CCC GGG GCT GCT GTT TAT A
CD133 reverse-5ʹ-ATC ACC AAC AGG GAG ATT G

### 4.6. Generation of Dox-Inducible Cells 

Dox-inducible lentivirus that can induce CD133 expression was generated by co-transfecting pLenti-CMV-rtTA3 Blast (Addgene, Watertown, MA, USA), psPAX2, and pMD2.G (5 µg each; Addgene, Watertown, MA, USA) into HEK293FT packaging cells in the presence or absence of pLV-EGFP/Neo- TRE3G-CD133 (10 µg; VectorBuilder Inc., Chicago, IL, USA) using Lipofectamine LTX (ThermoFisher Sci, Waltham, MA, USA) according to the manufacturer’s specifications. The medium was replaced after 16 h and cells were incubated for another 48 h to generate the lentivirus. Viral supernatant was then collected, concentrated by centrifugation, and filter-sterilized prior to use. For the virus transduction, viral supernatants were added to the media (MOI = 1) in 6-well plates seeded with cells. Media was replaced with cell culture media after 24 h, and transduced cells were then selected for 10 days with blasticidin (40 µg/mL) and gentamicin (1000 µg/mL) for 10 days as previously described [58]. Dox-inducible BAKP-CD133 cells were then fluorescently tagged by stable transfection with pCMS-GFP. 

### 4.7. Drug Treatment and Cell Viability Assays

Cells were plated at 5000 cells/well in 96-well plates and triplicate wells were exposed to indicated concentrations of kinase inhibitors trametinib or dabrafenib, or DTIC for up to 72 h. Drugs were diluted and dissolved in the same final volume of DMSO with a final concentration of 0.2% DMSO in a culture medium. As a negative control, cells were exposed to 0.2% DMSO alone in the medium. To determine the effects of drugs on cell viability, a colorimetric XTT assay was used (Biotium, Inc., Freemont, CA, USA) that measures the metabolic reduction of XTT by viable cells. Absorbance readings were taken every hour for up to 3 h after XTT reagent addition using a Victor plate reader set at 450 nm to obtain a slope of A_450_/min. Each plate included wells with the drug-treated cells in triplicate, together with six replicates each of increasing number of cells in IMDM medium/0.2% DMSO to generate a standard curve of A_450_/min vs. cell number. The standard curve was then used to generate the drug dose-response curves. Data presented in each figure represent the mean ± SD of each set of triplicate drug-treated cells from a representative experiment.

### 4.8. Annexin V/PI Staining and Flow Cytometry

Cytosolic extracts were derived from pooled attached and floating cells and subjected to fluorometric caspase-3 activity assays with a fluorescent tetrapeptide substrate specific for caspases-3 (Ac-DEVD-aminomethylcoumarin (AMC, Enzo Life Sciences, Farmingdale, NY, USA)) as previously described [73], followed by flow cytometric analysis in a Becton-Dickinson FACStar Plus flow cytometer.

### 4.9. Fluorometric Caspase-3 Activity Assays 

Cytosolic extracts, derived from pooled attached and floating cells, were subjected to fluorometric caspase-3 activity assays with a fluorescent tetrapeptide substrate specific for caspases-3 (Ac-DEVD-aminomethylcoumarin (AMC, Enzo Life Sciences, Farmingdale, NY, USA)) as previously described [63]. Free AMC from cleavage of the aspartate-AMC bond was measured in a kinetic assay over 30 min in a Wallac Victor3 fluorometer (Perkin-Elmer, Waltham, MA, USA) with emission and excitation and at 460 and 360 nm, respectively. Caspase activity was determined after linear regression analysis of the initial velocity (slope) for each curve showing emission from each sample plotted against time.

### 4.10. Live FLICA Caspase-3/7 Activity Assays

Cells (50,000 cells per well) were plated in 6-well plates, and triplicate wells were incubated for 24 h with or without Dox in the presence or absence of trametinib (100 nM), together with a FLICA caspase 3/7 detection reagent with a sulforhodamine group reporter (Image-iT Live Red Caspase 3/7 Detection kit; ThermoFisher, Waltham, MA, USA), in an EVOS FL Auto Imaging System (ThermoFisher, Waltham, MA, USA) with an onstage incubator. Cells were fixed after 24 h, and the % FLICA-positive cells, indicative of cells with active caspase 3, were imaged and quantified using the EVOS FL Auto Imaging System. 

### 4.11. Immunoblot Analysis

SDS-Page electrophoresis and transfer of proteins to nitrocellulose membranes were performed according to standard procedures. Ponceau S (0.1%) staining of membranes verified transfer of proteins and equal loading. Membranes were incubated with antibodies to CD133 (Miltenyi Biotec, Auburn, CA, USA), cleaved and intact PARP, BAD, p-BAD, BCL-XL, MCL-1 (BioLegend, San Diego, CA, USA) cleaved active caspase-3, cleaved active caspase-9, BCL-2, active BAX (Novus Biologicals, Centennial, CO), AKT and p-AKT (Santa Cruz Biotech, Dallas, TX, USA), ERK, p-ERK, or to β-Actin (ProteinTech, Rosemont, IL, USA) as a loading control. Immunoblots were sequentially reprobed with other antibodies after stripping them of antibodies. Immune complexes were detected by incubation with horseradish peroxidase-conjugated antibodies to mouse or rabbit IgG (1:3000), followed by enhanced chemiluminescence (ECL; Pierce, Rockford, IL, USA) and imaging in a GE Healthcare Amersham Imager 600. 

### 4.12. Statistical Analysis

All experiments were performed in triplicate. Error bars show SD; a standardized t-test determined *p*-values. *p* < 0.05, *p* < 0.01, *p* < 0.001, or *p* < 0.0001 are denoted with one to four asterisks, and *p* < 0.05 was considered significant. 

## 5. Conclusions

**Figure 8:**
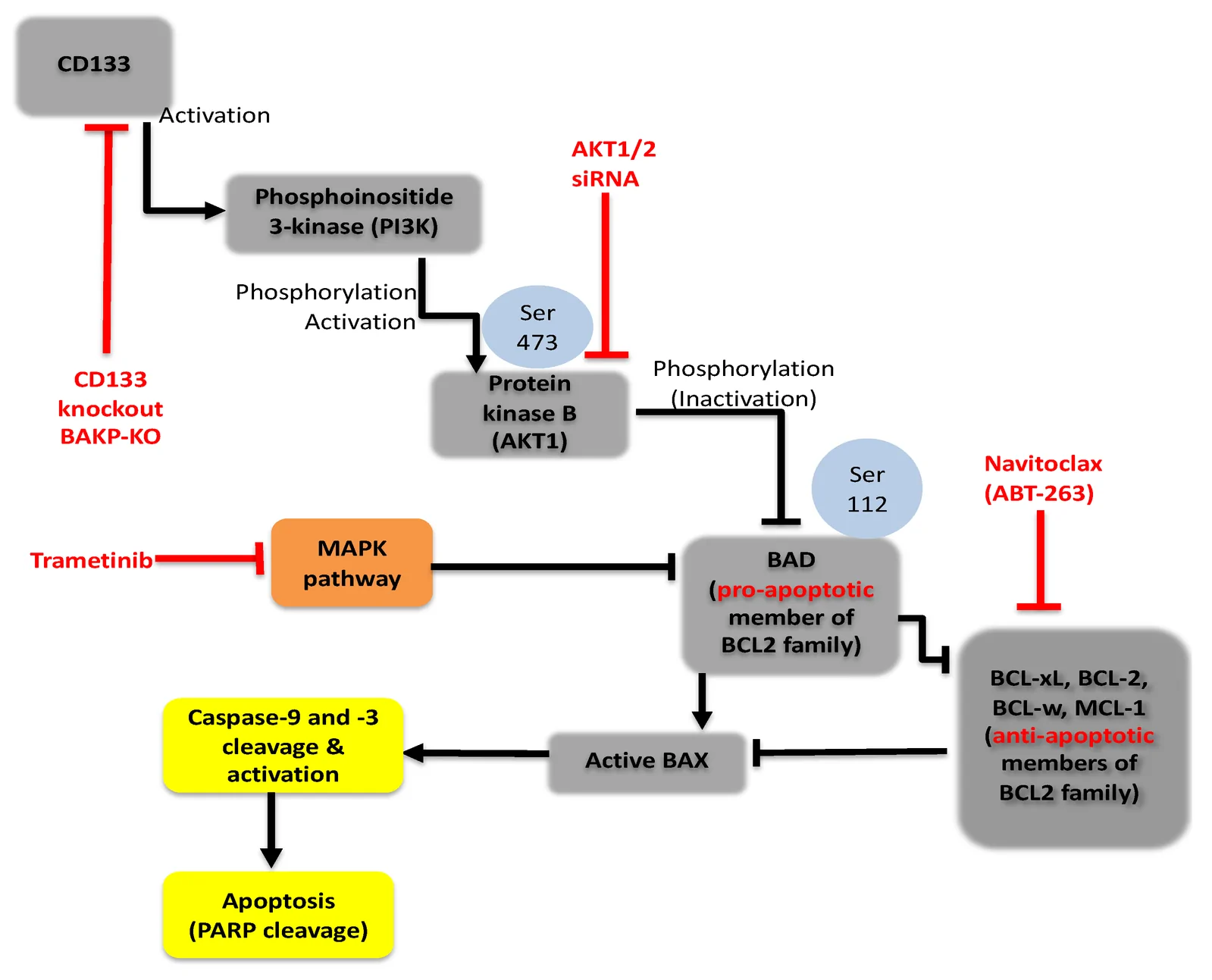
PI3K/Akt/ Bcl2 family pro-survival signaling pathway in CD133-positive melanoma initiating stem cells (MICs). CD133 may activate a survival pathway where (1) increased phosphorylation of AKT induces (2) phosphorylation and inactivation of BAD, (3) decreases the active form of BAX, and (4) reduces caspase activation and caspase-mediated PARP cleavage, indicating apoptosis suppression leading to drug resistance in melanomas.

CRISPR-cas9 technology was used in the current study to delineate the mechanistic molecular pathway(s) by which CD133 promotes cell survival and resistance to targeted kinase inhibitors such as trametinib in melanoma. CD133-overexpressing cancer stem cells promote cell survival by suppressing trametinib-induced caspase-3 mediated apoptosis which is reversed by the CRISPR-Cas9 knockdown of CD133 expression. These results are reproduced in different patient-derived melanoma cell lines. Apoptosis suppression is regained in these cell lines by the Dox-inducible expression of CD133. Together, the data shows that CD133 activates a pro-survival CD133/AKT/BCL2 family-dependent pathway that works in combination with the MAPK pathway to potently inhibit active BAX/caspase-9-mediated apoptosis (Figure 8). These combined data suggest a mechanism for trametinib resistance in CD133(+) melanoma stem cells and lend strong support for the use of CD133 as a target for combinatorial therapeutic interventions.

## Supplementary Materials

The following supporting information can be downloaded at: https://www.mdpi.com/article/10.3390/ijms23042333/s1.
