# Correction: Simbulan-Rosenthal et al. Employing CRISPR-Cas9 to Generate CD133 Synthetic Lethal Melanoma Stem Cells. *Int. J. Mol. Sci.* 2022, *23*, 2333

**DOI:** 10.3390/ijms27177643

**Published:** 2026-08-26

**Authors:** Cynthia M. Simbulan-Rosenthal, Yogameenakshi Haribabu, Sahar Vakili, Li-Wei Kuo, Havens Clark, Ryan Dougherty, Ryyan Alobaidi, Bonnie Carney, Peter Sykora, Dean S. Rosenthal

**Affiliations:** 1 Department of Biochemistry and Molecular & Cellular Biology, Georgetown University School of Medicine, Washington, DC 20057, USA; simbulac@georgetown.edu (C.M.S.-R.);; 2 Firefighters’ Burn and Surgical Laboratory, MedStar Health Research Institute, Washington, DC 20010, USA; 3 Amelia Technologies, LLC, 1121 5th St. NW, Washington, DC 20001, USA

## Figure Correction

In the original publication [1], there was a mistake in Figure 2 as published. The same RT-PCR data was used in Figure 2A of the original publication [1] as that in *Cancers* 2019 Figure 4B [2], although we referenced the earlier *Cancers* paper [2] in the paragraph before Figure 2A of the *IJMS* paper page 6: “Due to more frameshift mutations in T3 compared to T1 and T2 cell lines, T3 cells (BAKR-KO) showed effective knockout of the CD133 protein and RNA [58] and were therefore used in subsequent experiments in this study.” Reference 58 is the *Cancers* 2019 paper [2]. Figure 2A was inverted to emphasize the smear in the T3 KO lane. 

Figure 2 has now been corrected by deleting Figure 2A, which was determined to be unnecessary, since the paper already referenced the *Cancers* 2019 paper [2] in describing the RT-PCR results in Results section above Figure 2. With the deletion of the original Figure 2A in Figure 2 described above, the figure legend for Figure 2 has also been corrected. The corrected Figure 2 and legend appear below, with Figure 2A deleted, and where Figure 2B is now Figure 2A, and Figure 2C is now Figure 2B. 

## Text Correction

A correction has been made to the text relevant to Figure 2: Results section, Section 2.2. CRISPR-Cas9 Knockout of CD133 in Conditionally Reprogrammed BAKR Cells Increases Apoptotic Caspase-3 Activation in Response to Trametinib via Bcl2 Downregulation, Paragraph 2:

RT-PCR with primers flanking the sgRNA target [58] and immunoblot analysis (Figure 2A) verified depletion of CD133 protein in BAKR-KO cells, compared to BAKR or scrambled sgRNA controls (BAKR-SC), after CRISPR-Cas9-associated sgRNAs targeting of exon 1 of the *CD133* gene. CD133 knockout in BAKR cells effectively reduced CD133 protein levels by 80% (Figure 2A). To compare the drug sensitivities of BAKR cells expressing the *CD133* gene (BAKR-SC) and CD133-depleted BAKR-KO, cells were exposed to increasing doses of trametinib and subjected to immunoblot analysis for apoptosis markers (caspase-3 proteolytic activation and PARP cleavage; Figure 2B).

The authors state that the interpretation of the results and scientific conclusions are unaffected by the revision. These corrections were approved by the Academic Editor. The original publication has also been updated.

## Figures and Tables

**Figure 2:**
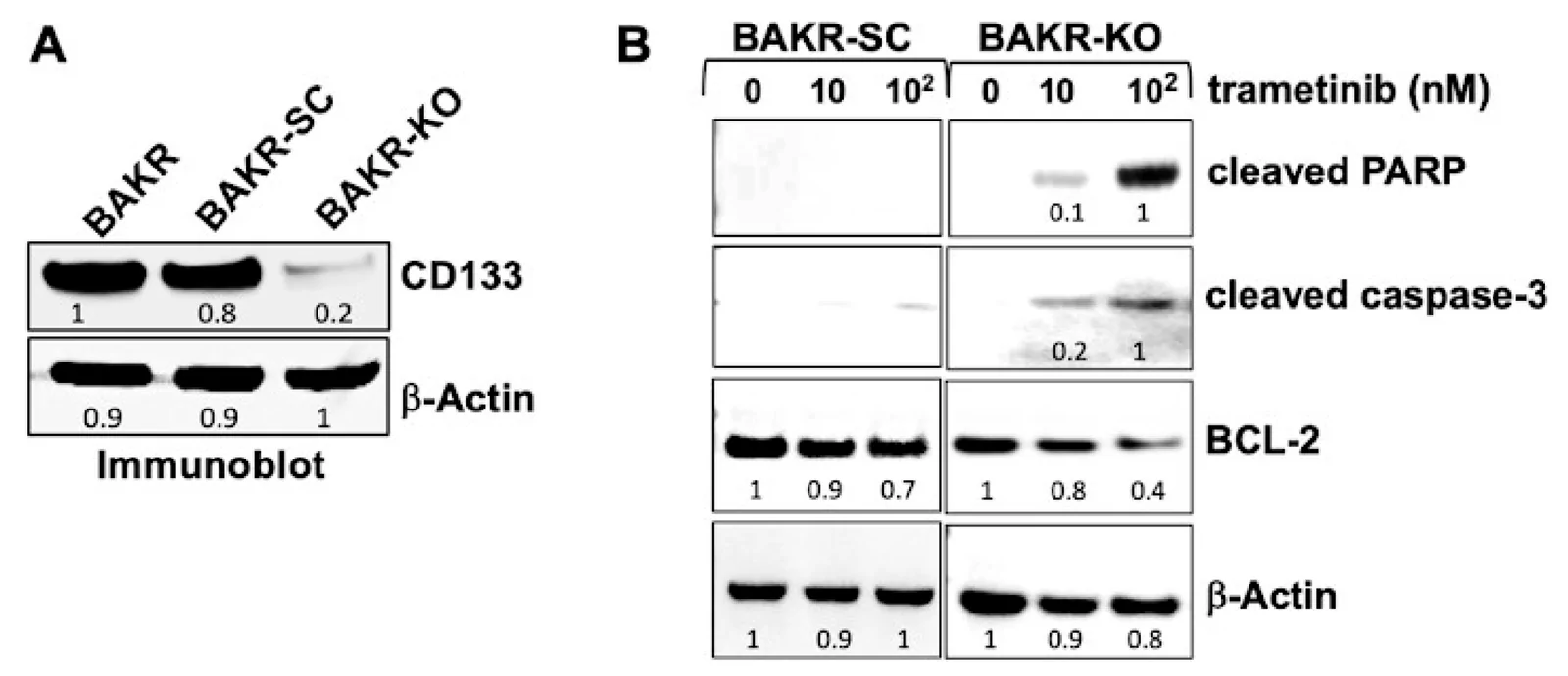
CRISPR-Cas9 CD133-knockout sensitizes BAKR-KO cells to trametinib-induced apoptosis via reduced BCL-2 levels. CD133 expression was compared between BAKR, CD133-knockout BAKR-KO, and sgRNA control BAKR-SC cells. (**A**) Immunoblot analysis confirms depletion of CD133 protein in BAKR-KO, compared to BAKR-SC and BAKR cells. (**B**) BAKR-SC and BAKR-KO cells were exposed to increasing trametinib concentrations for 72 h and subjected to immunoblot analysis with antibodies to the active cleaved form of caspase-3 (p17), cleaved PARP, BCL-2, and β-Actin for loading control. Densitometric analysis comparing intensities of protein bands relative to bands with the highest intensities is shown in immunoblots.
