# Supplementary material for: Employing CRISPR-Cas9 to Generate CD133 Synthetic Lethal Melanoma Stem Cells
Source: Int J Mol Sci. 2022 Feb 20;23(4):2333. doi: 10.3390/ijms23042333 (PMC8877091; doi:10.3390/ijms23042333)
Supplement: Supplementary file 1 [file ijms-23-02333-s001.zip › ijms-1603715-supplementary.pdf]

Supplementary Material S1:
Scans of whole gel western blots showing molecular weight sizes of relevant proteins (indicated in arrows)

β Actin 45 kDa

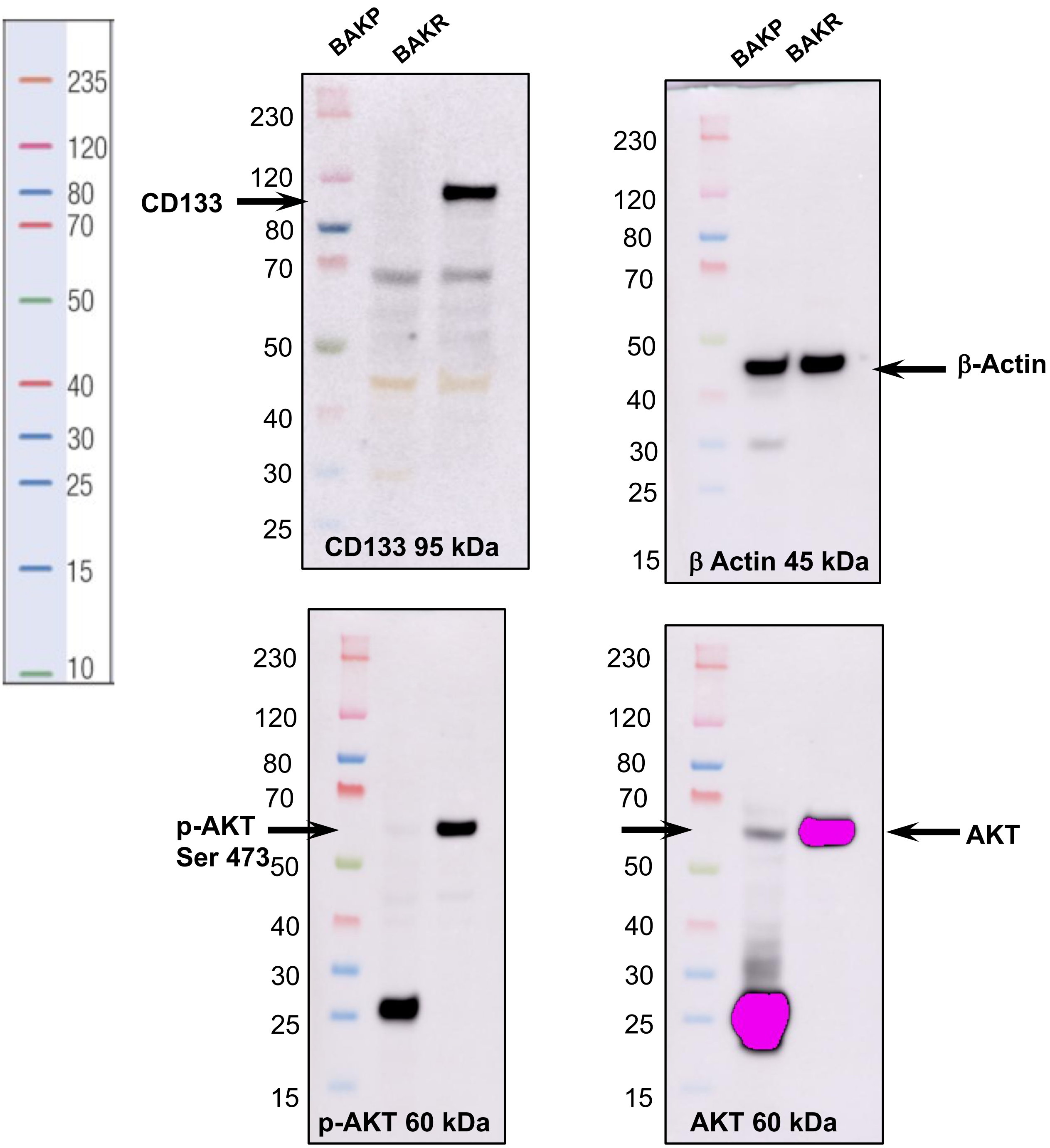

Figure S1A

β Actin 45 kDa

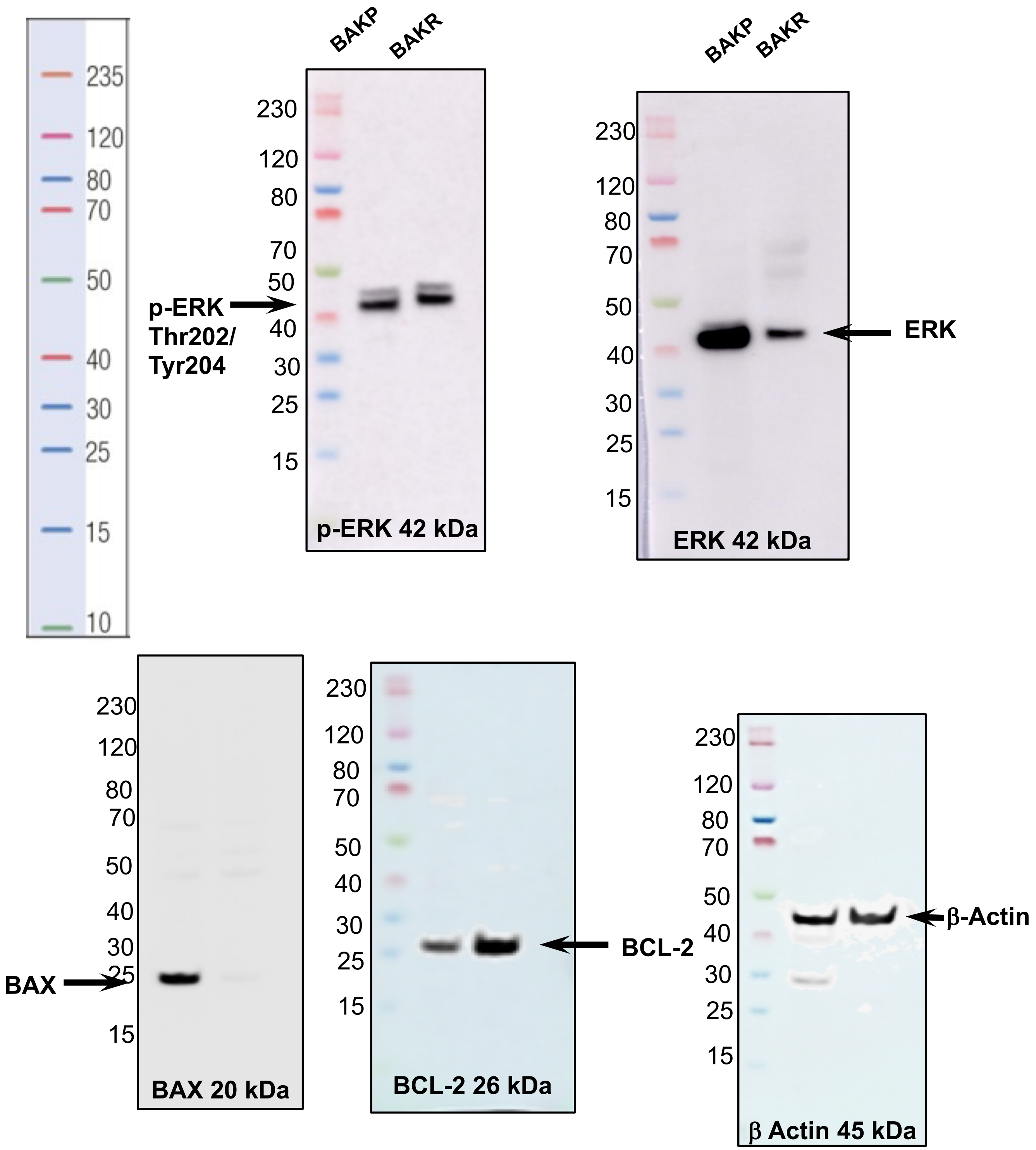

Figure S1B

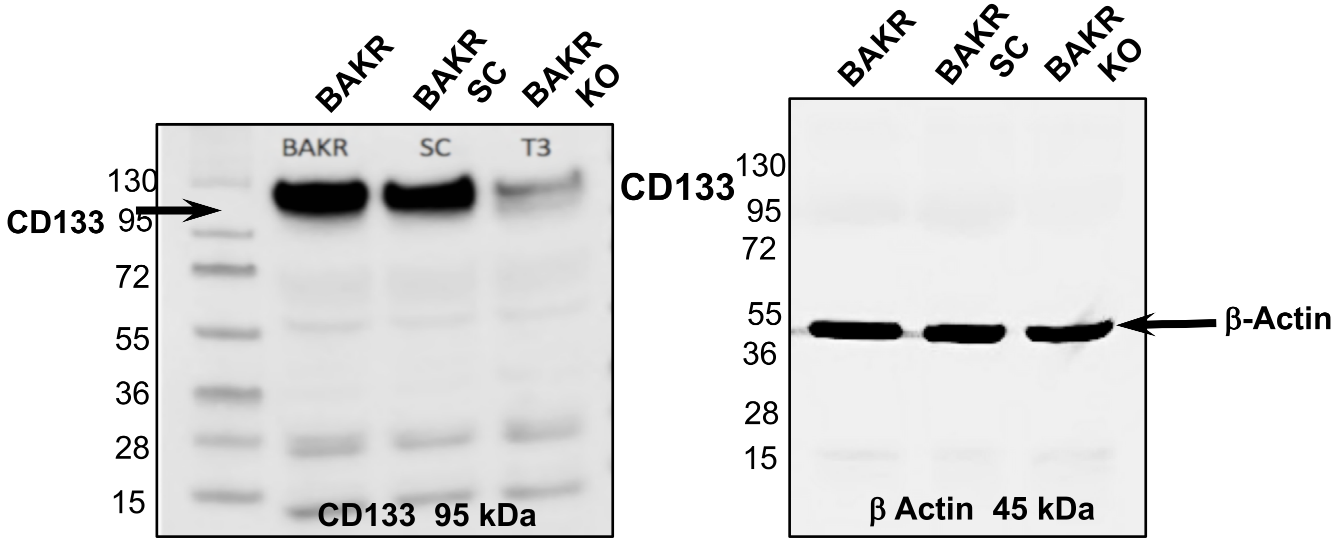
BAKR
BAKR SC
BAKR KO
BAKR
SC
T3
130
CD133
95
72
55
36
28
15
CD133 95 kDa
BAKR
BAKR SC
BAKR KO
CD133
130
95
72
55
36
28
15
β-Actin
β Actin 45 kDa

Figure S2A

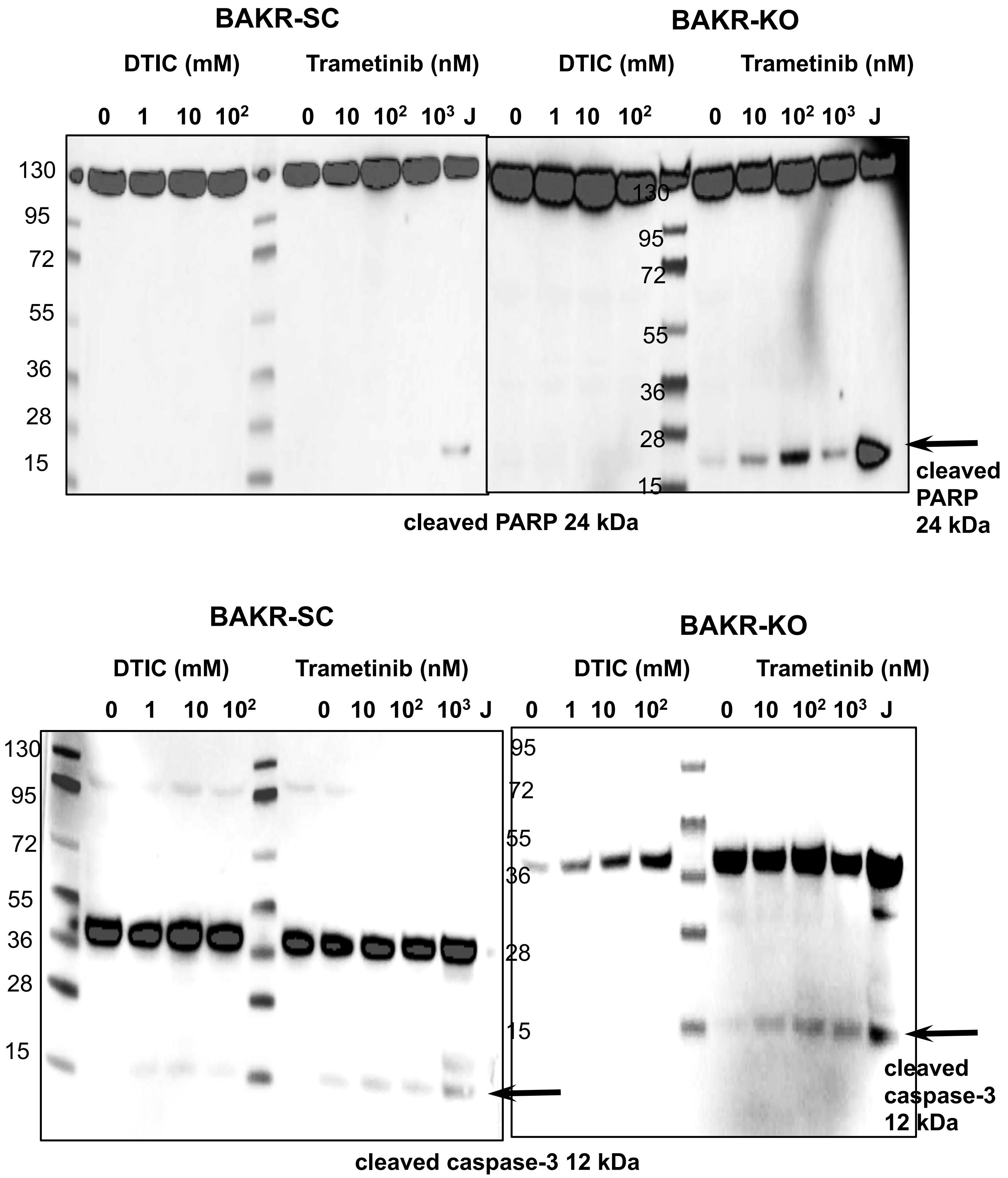
BAKR-SC
DTIC (mM)
Trametinib (nM)
0 1 10 10²
0 10 10² 10³ J
BAKR-KO
DTIC (mM)
Trametinib (nM)
0 1 10 10²
0 10 10² 10³ J
130
95
72
55
36
28
15
cleaved PARP 24 kDa
cleaved PARP 24 kDa
BAKR-SC
DTIC (mM)
Trametinib (nM)
0 1 10 10²
0 10 10² 10³ J
BAKR-KO
DTIC (mM)
Trametinib (nM)
0 1 10 10²
0 10 10² 10³ J
130
95
72
55
36
28
15
cleaved caspase-3 12 kDa
cleaved caspase-3 12 kDa

Figure S2B

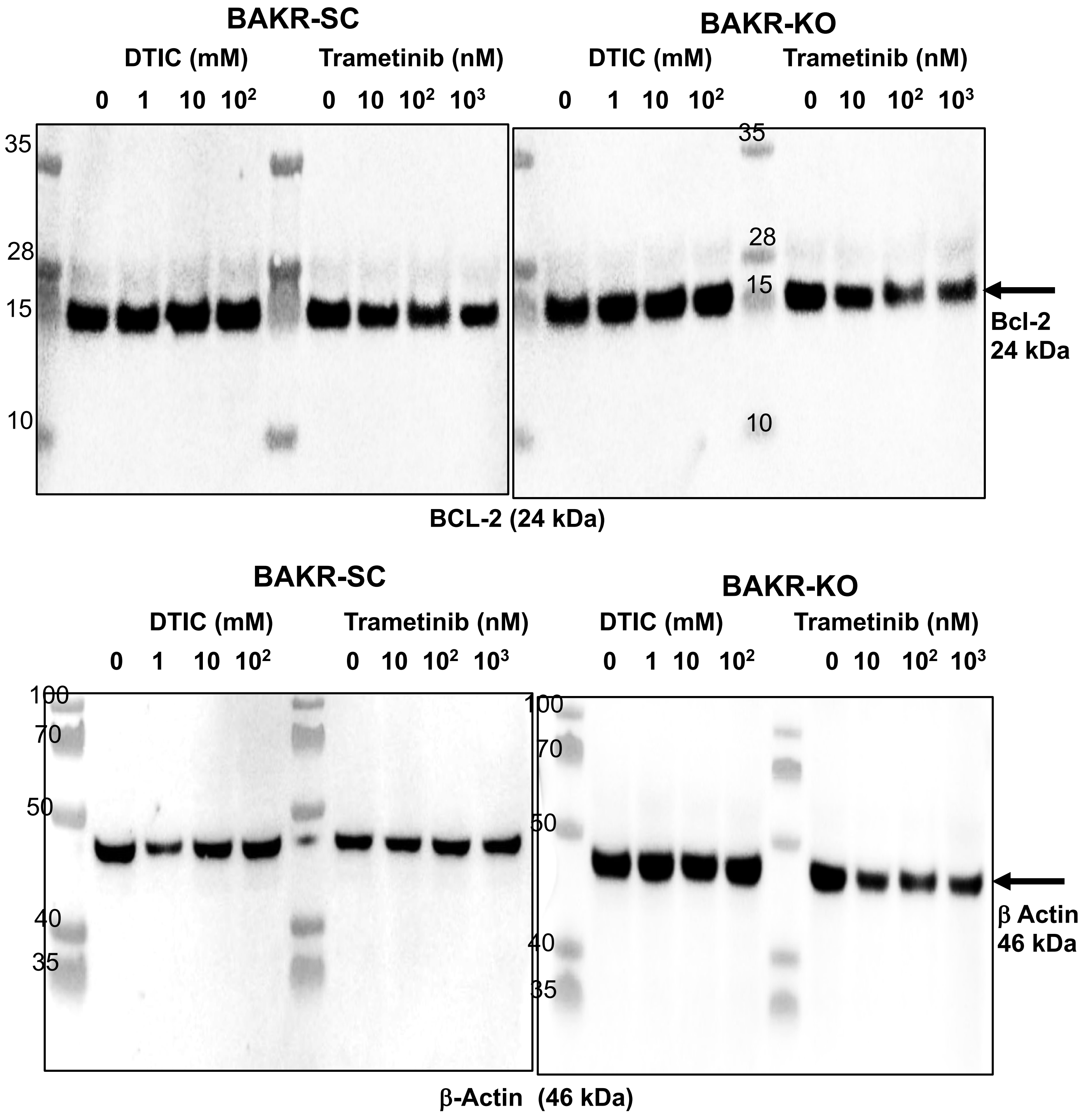
BAKR-SC
DTIC (mM)
Trametinib (nM)
0 1 10 10^2
0 10 10^2 10^3
BAKR-KO
DTIC (mM)
Trametinib (nM)
0 1 10 10^2
0 10 10^2 10^3
35
28
15
10
Bcl-2
24 kDa
BCL-2 (24 kDa)
BAKR-SC
DTIC (mM)
Trametinib (nM)
0 1 10 10^2
0 10 10^2 10^3
BAKR-KO
DTIC (mM)
Trametinib (nM)
0 1 10 10^2
0 10 10^2 10^3
100
70
50
40
35
β Actin
46 kDa
β-Actin (46 kDa)

Figure S2C

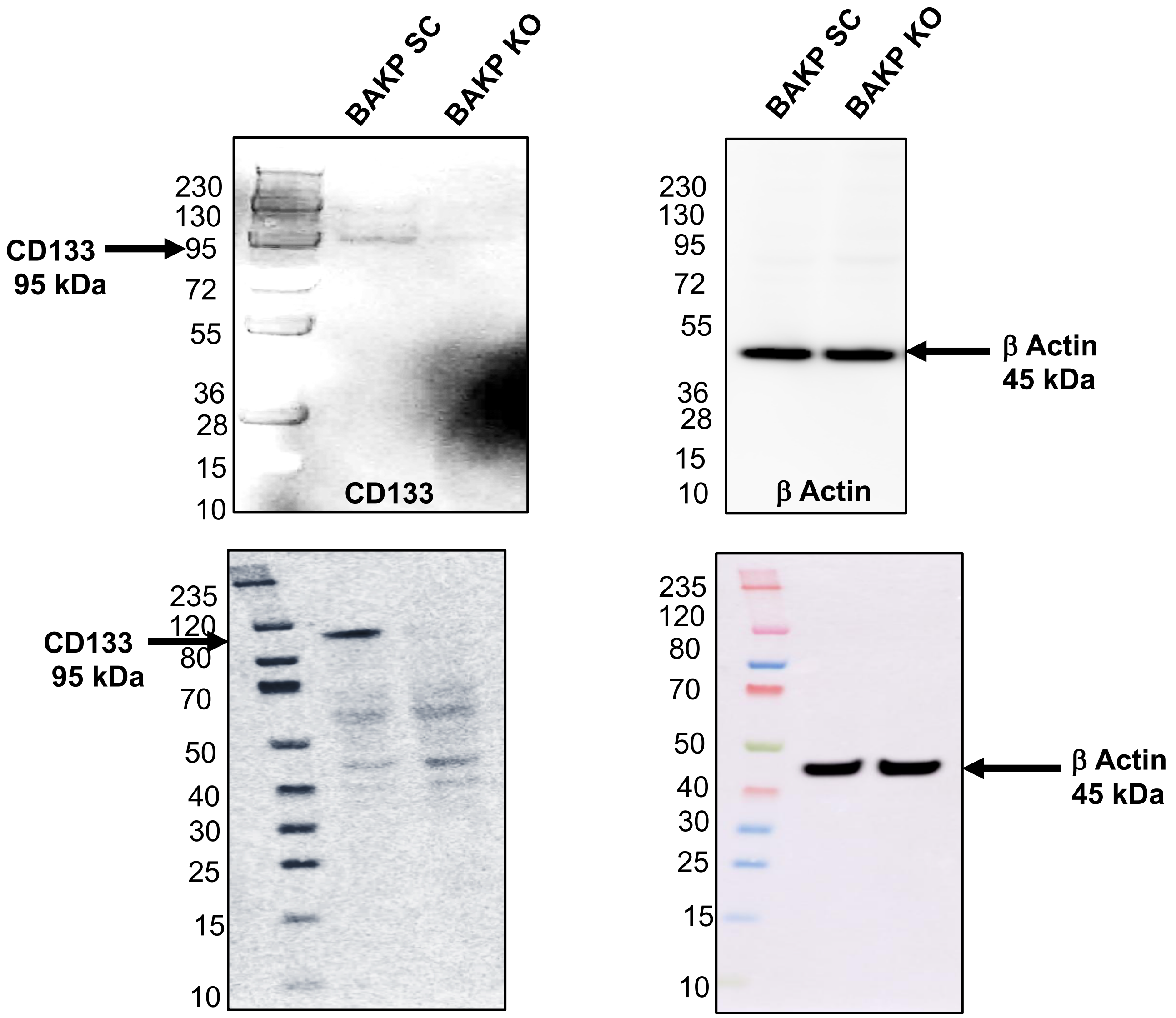
BAKP SC
BAKP KO
230
130
95
72
55
36
28
15
10
CD133
95 kDa
CD133
BAKP SC
BAKP KO
230
130
95
72
55
36
28
15
10
β Actin
45 kDa
β Actin
235
120
80
70
50
40
30
25
15
10
CD133
95 kDa
235
120
80
70
50
40
30
25
15
10
β Actin
45 kDa

Figure S3

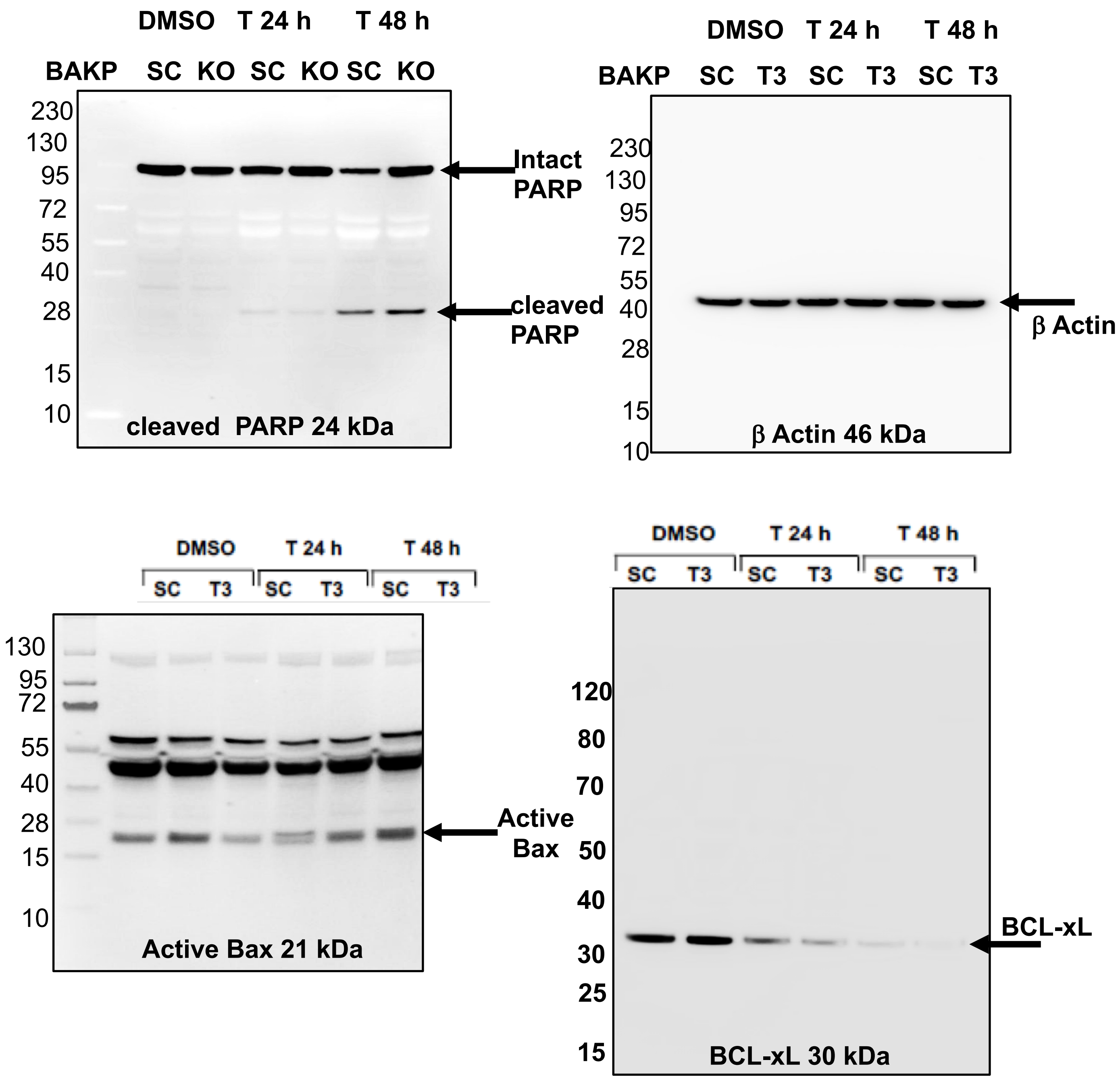
DMSO T 24 h T 48 h
BAKP SC KO SC KO SC KO
230
130
95
72
55
40
28
15
10
Intact PARP
cleaved PARP
cleaved PARP 24 kDa
DMSO T 24 h T 48 h
BAKP SC T3 SC T3 SC T3
230
130
95
72
55
40
28
15
10
β Actin
β Actin 46 kDa
DMSO T 24 h T 48 h
SC T3 SC T3 SC T3
130
95
72
55
40
28
15
10
Active Bax
Active Bax 21 kDa
DMSO T 24 h T 48 h
SC T3 SC T3 SC T3
120
80
70
50
40
30
25
15
BCL-xL
BCL-xL 30 kDa

Figure S4A

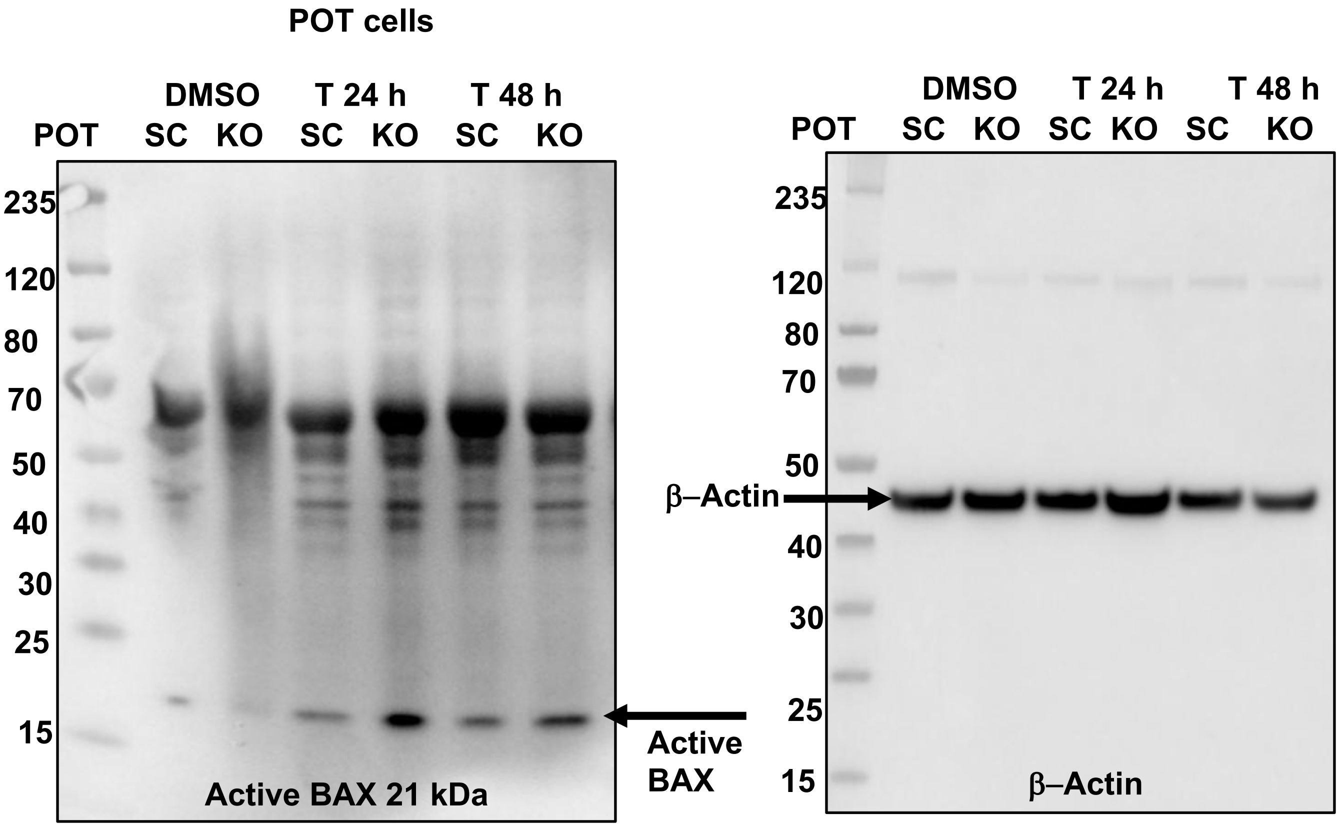

Figure S4B

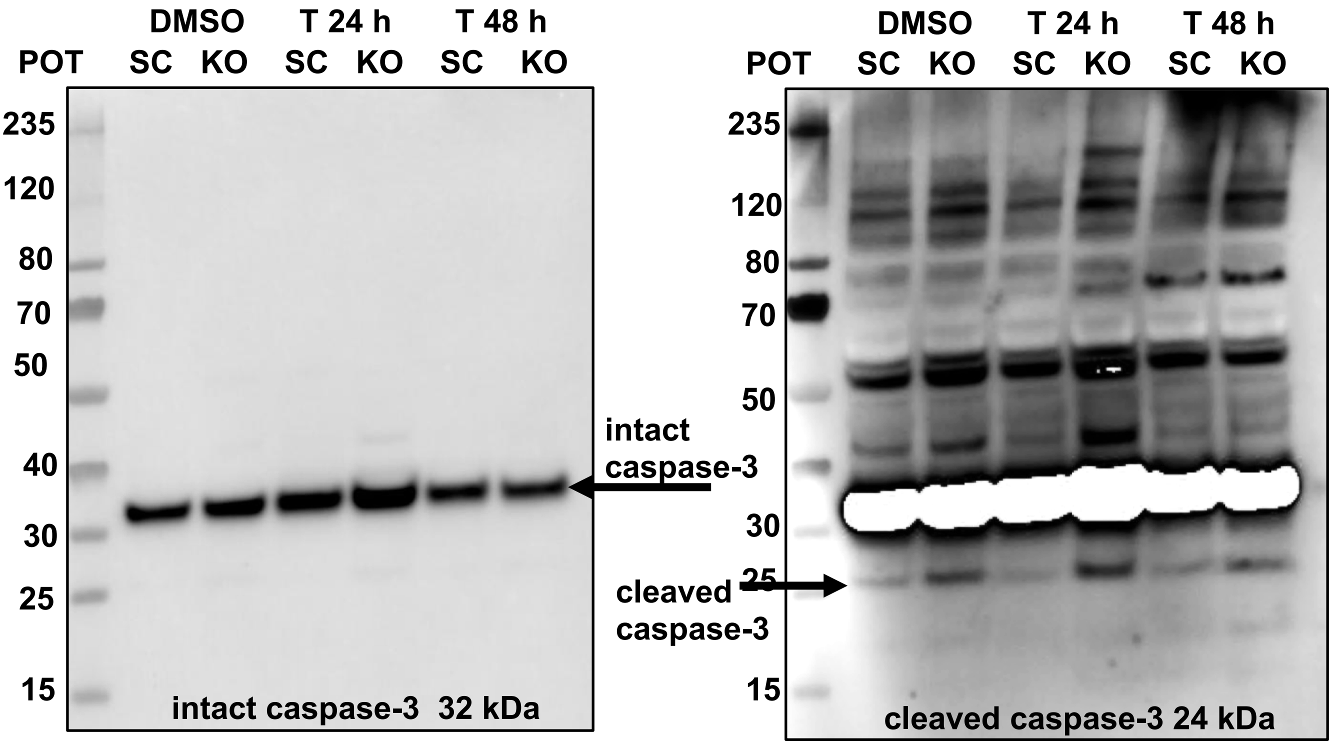

Figure S4C

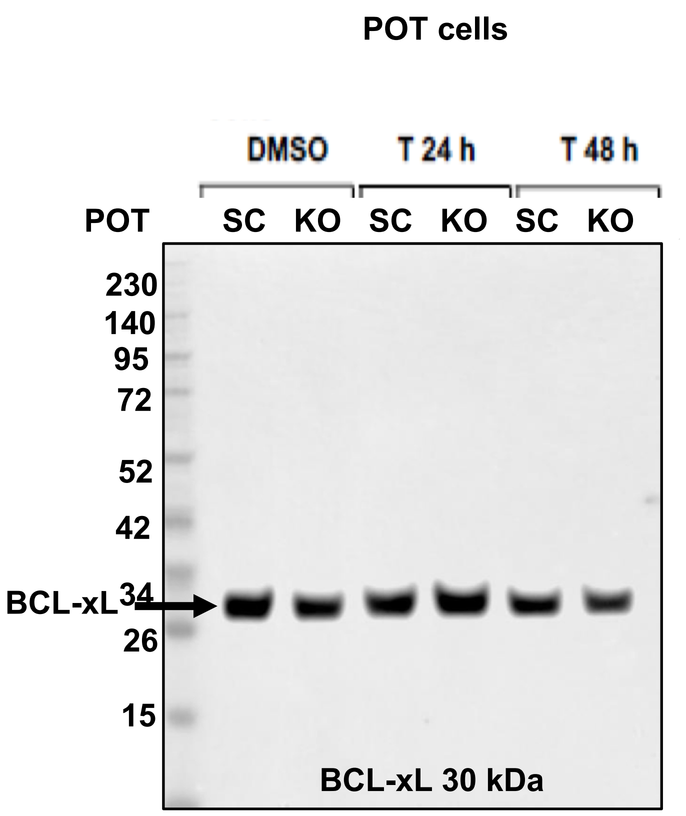
POT cells
DMSO
T 24 h
T 48 h
POT
SC
KO
SC
KO
SC
KO
230
140
95
72
52
42
34
BCL-xL
26
15
BCL-xL 30 kDa

Figure S4D

BAKP

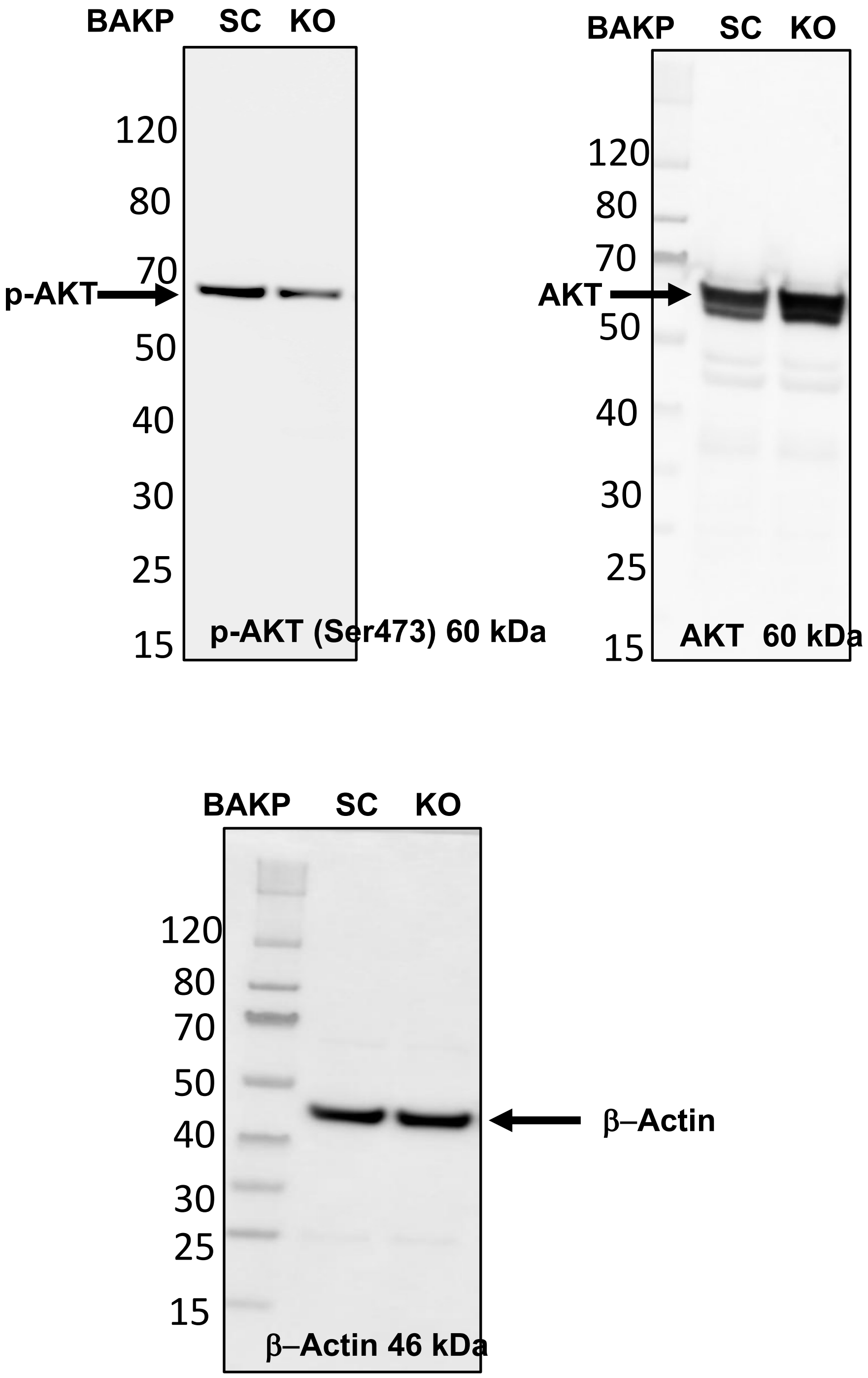

Figure S4E

BAKP

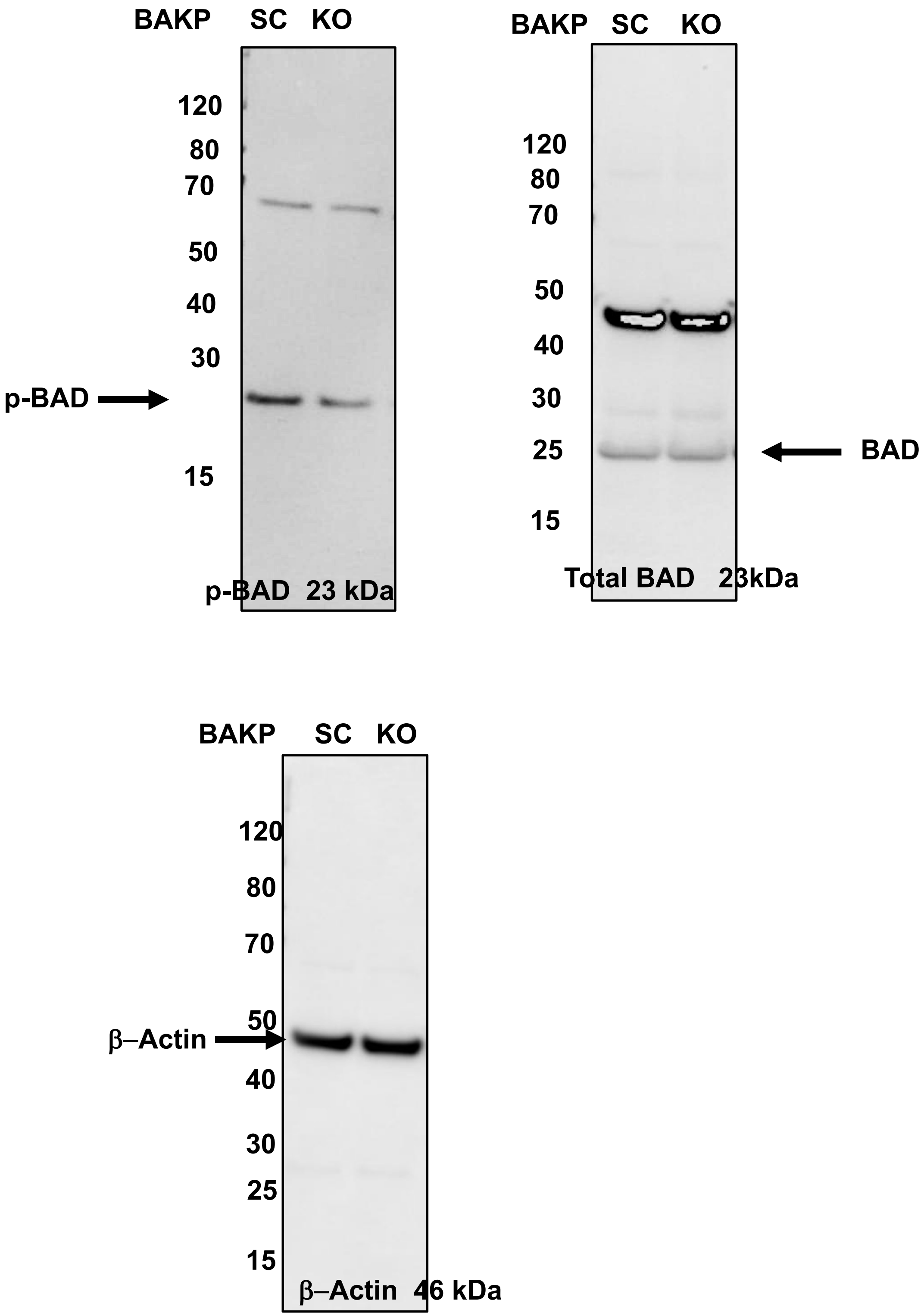

Figure S4F

POT

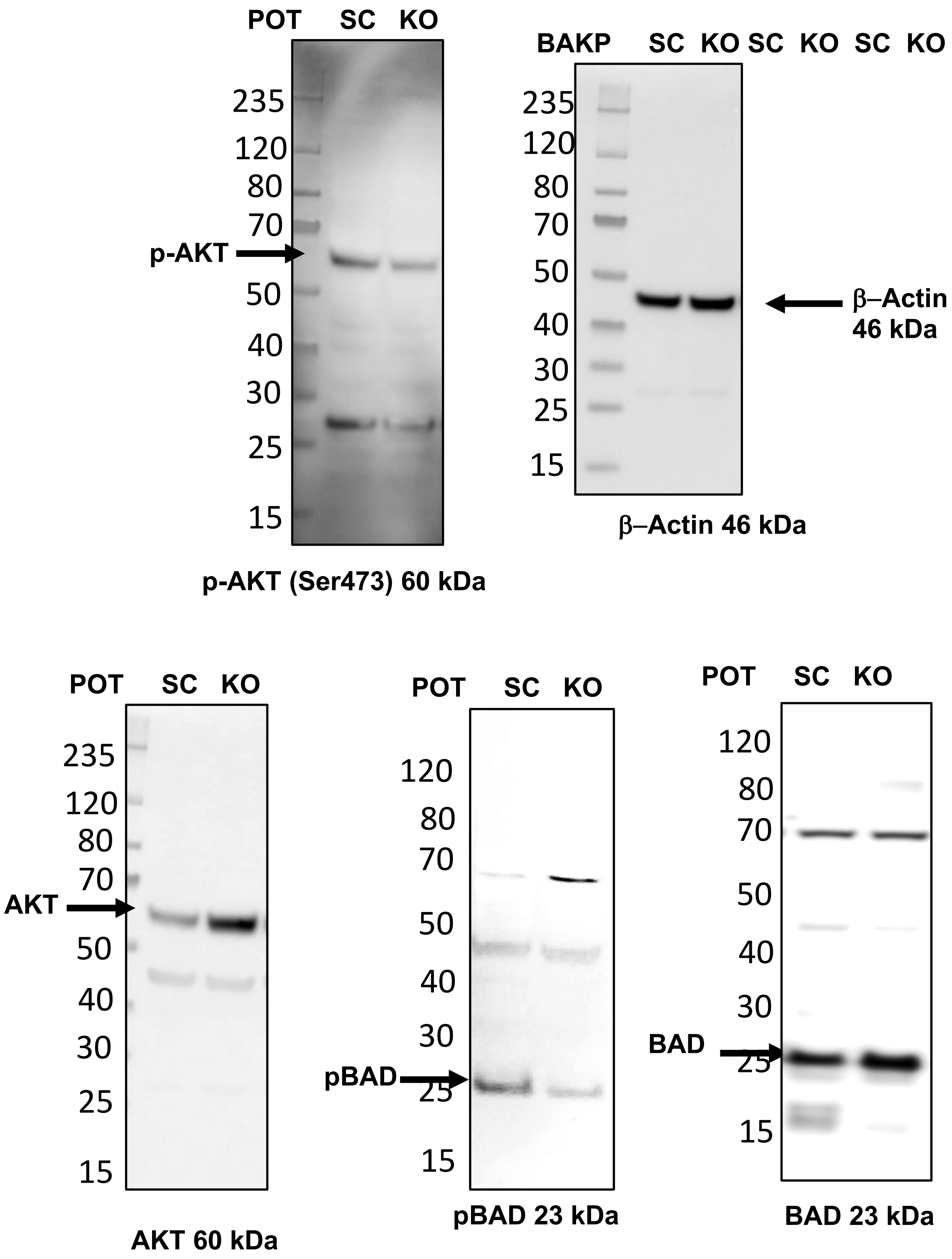

Figure S4G

BAKP-CD133

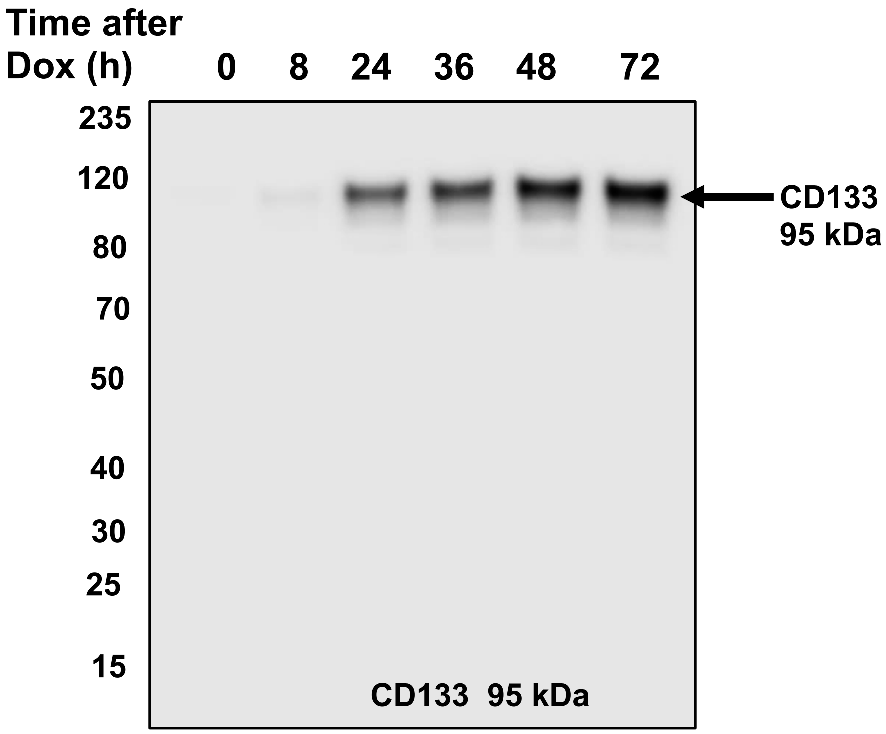

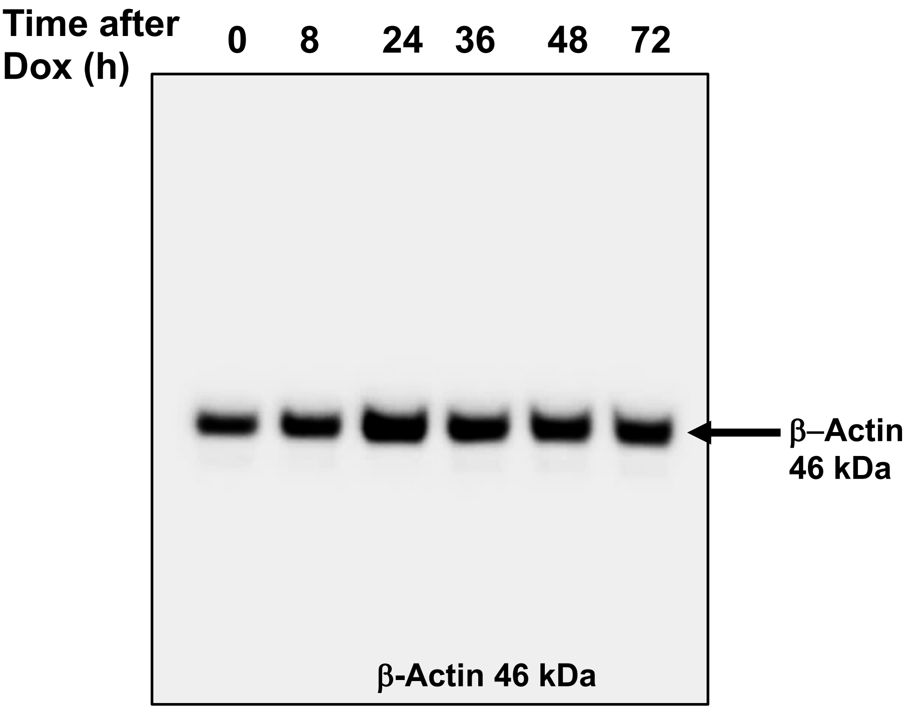

Figure S5A

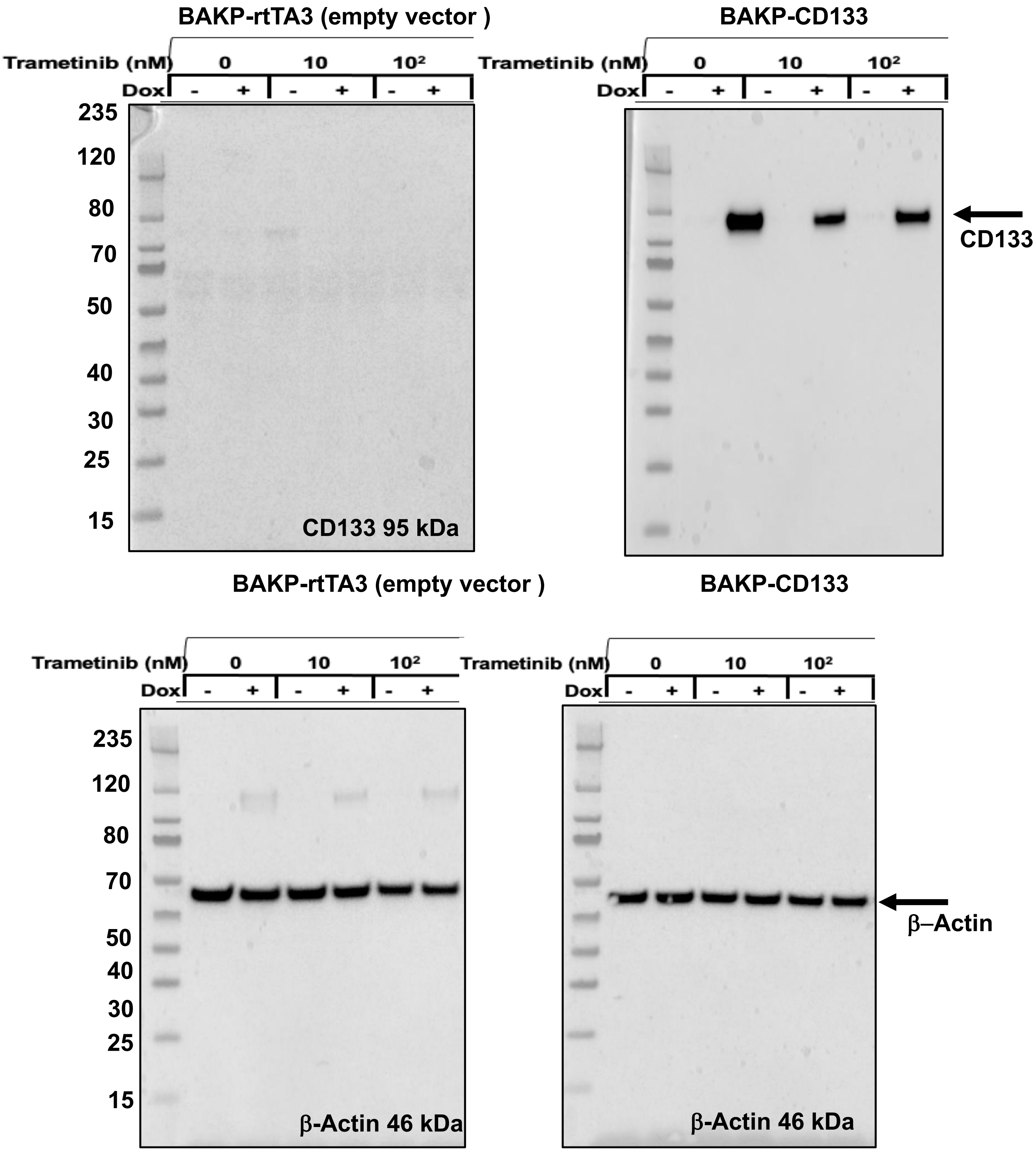
BAKP-rtTA3 (empty vector)
BAKP-CD133
Trametinib (nM) 0 10 10²
Dox - + - + - +
235
120
80
70
50
40
30
25
15
CD133 95 kDa
CD133
β-Actin 46 kDa
β–Actin

Figure S5B

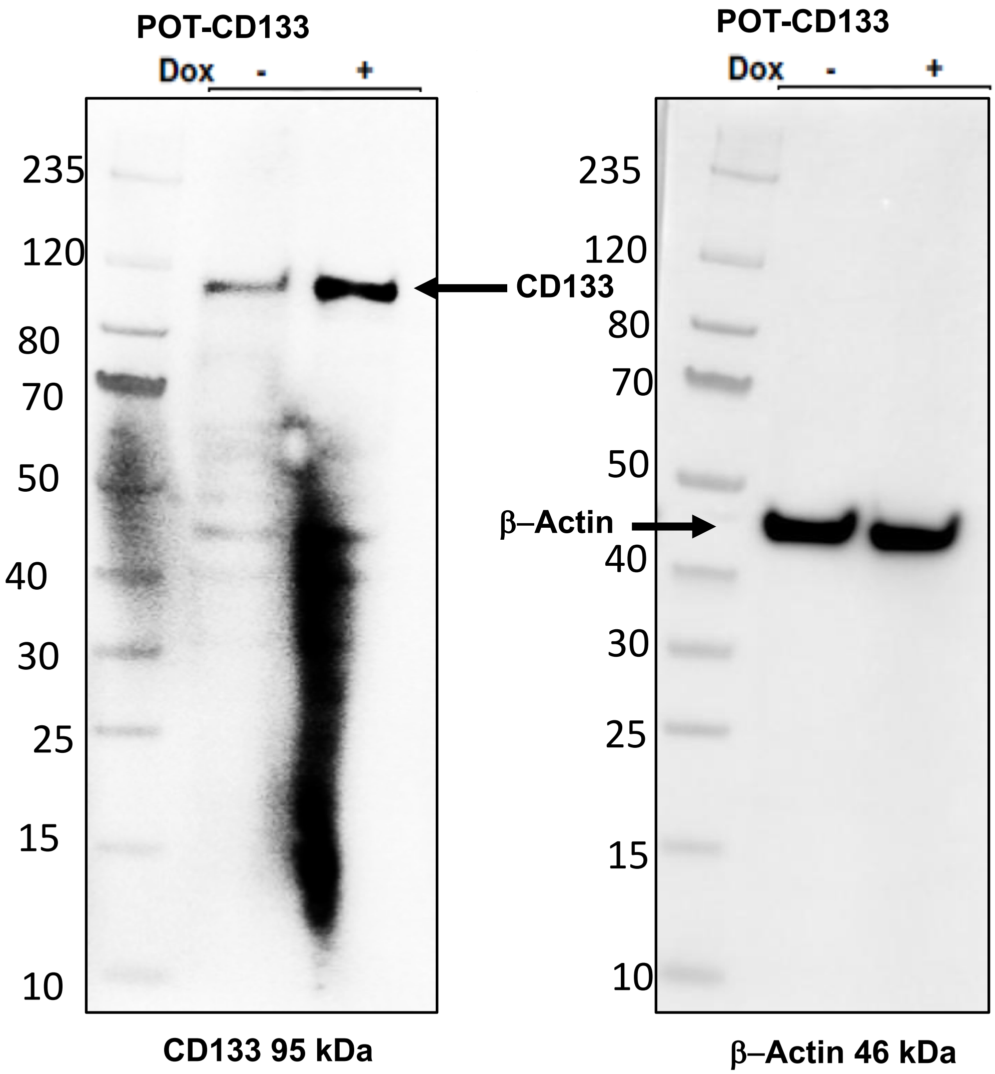
POT-CD133
Dox
-
+
235
120
80
70
50
40
30
25
15
10
CD133
CD133 95 kDa
POT-CD133
Dox
-
+
235
120
80
70
50
β–Actin
40
30
25
15
10
β–Actin 46 kDa

Figure S5C

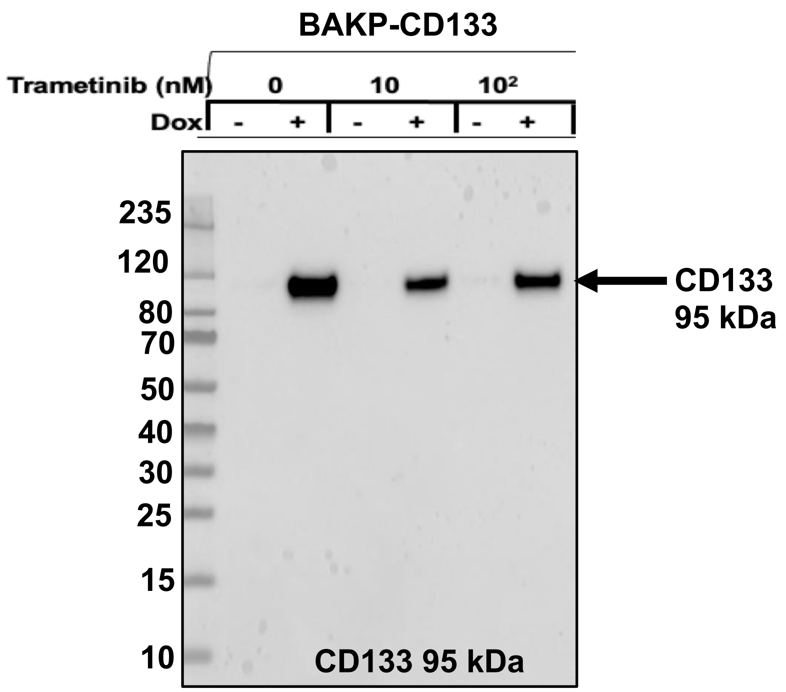
BAKP-CD133
Trametinib (nM)
0
10
10²
Dox
-
+
-
+
-
+
235
120
80
70
50
40
30
25
15
10
CD133 95 kDa
CD133
95 kDa

Figure S5D

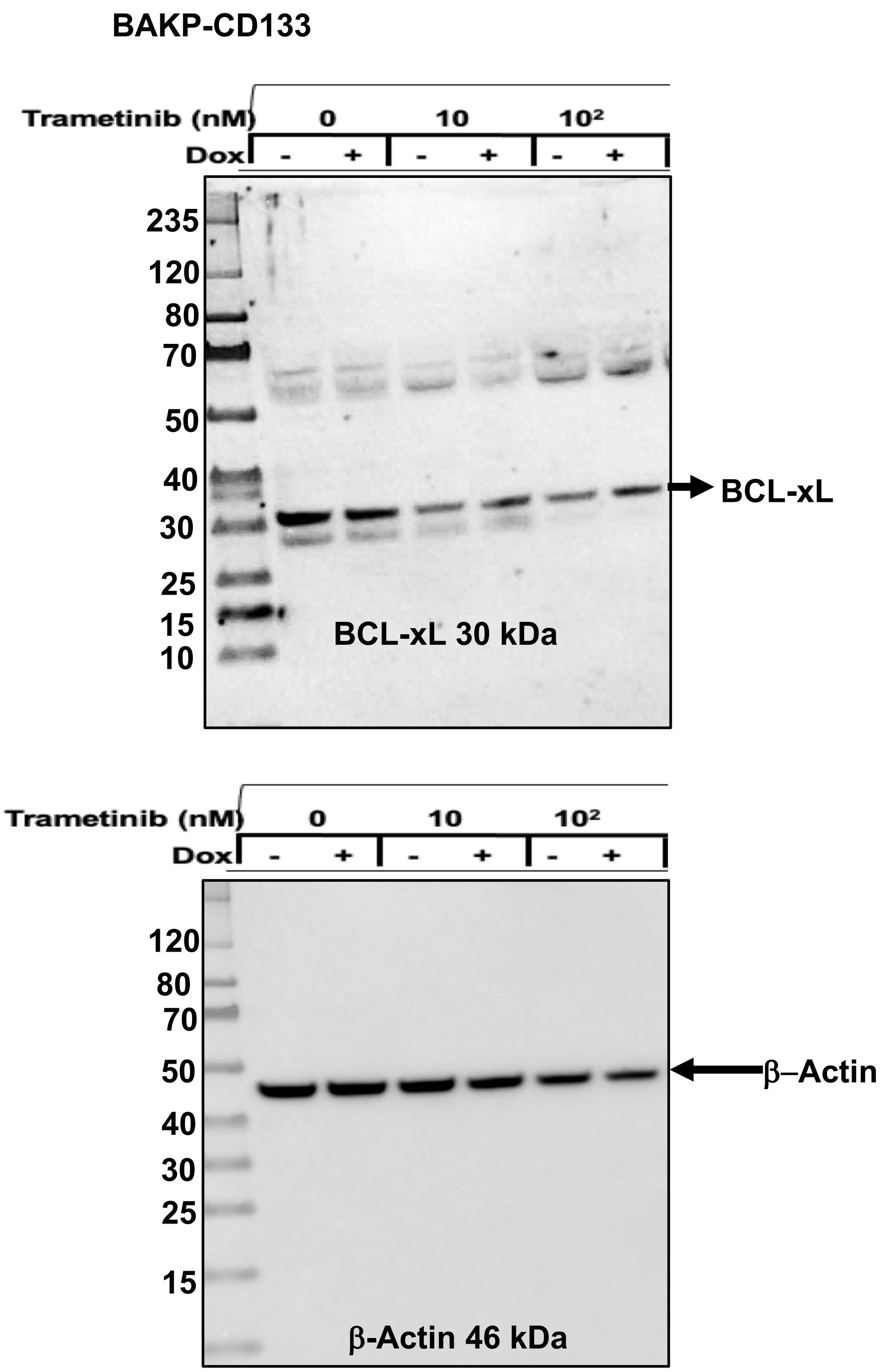
BAKP-CD133
Trametinib (nM)
0
10
10²
Dox
-
+
-
+
-
+
235
120
80
70
50
40
30
25
15
10
BCL-xL
BCL-xL 30 kDa
Trametinib (nM)
0
10
10²
Dox
-
+
-
+
-
+
120
80
70
50
40
30
25
15
β–Actin
β-Actin 46 kDa

Figure S5E

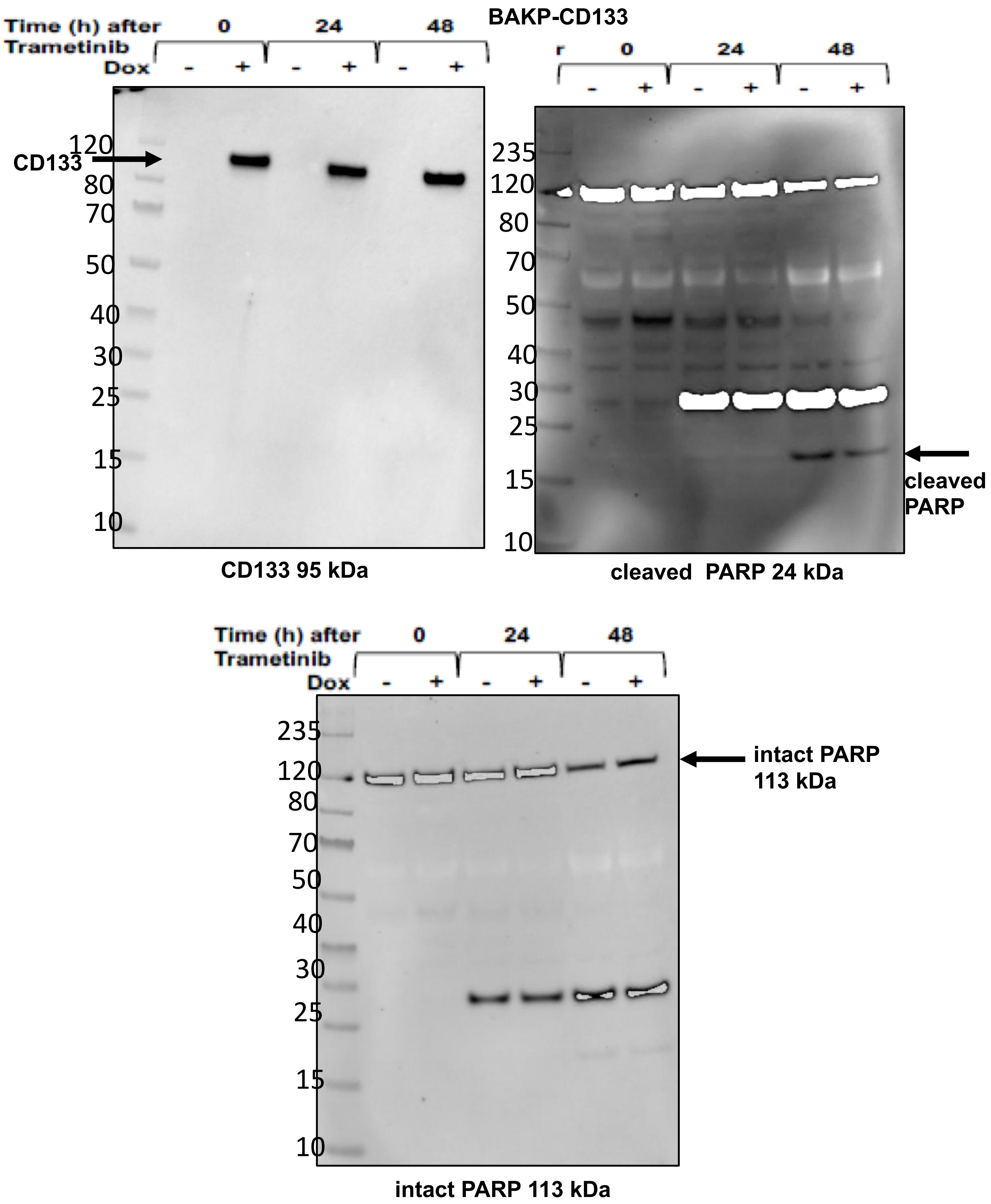
Time (h) after Trametinib
0
24
48
Dox
- + - + - +
CD133
120
80
70
50
40
30
25
15
10
CD133 95 kDa
BAKP-CD133
0
24
48
- + - + - +
235
120
80
70
50
40
30
25
15
10
cleaved PARP
cleaved PARP 24 kDa
Time (h) after Trametinib
0
24
48
Dox
- + - + - +
235
120
80
70
50
40
30
25
15
10
intact PARP 113 kDa
intact PARP 113 kDa

Figure S6A

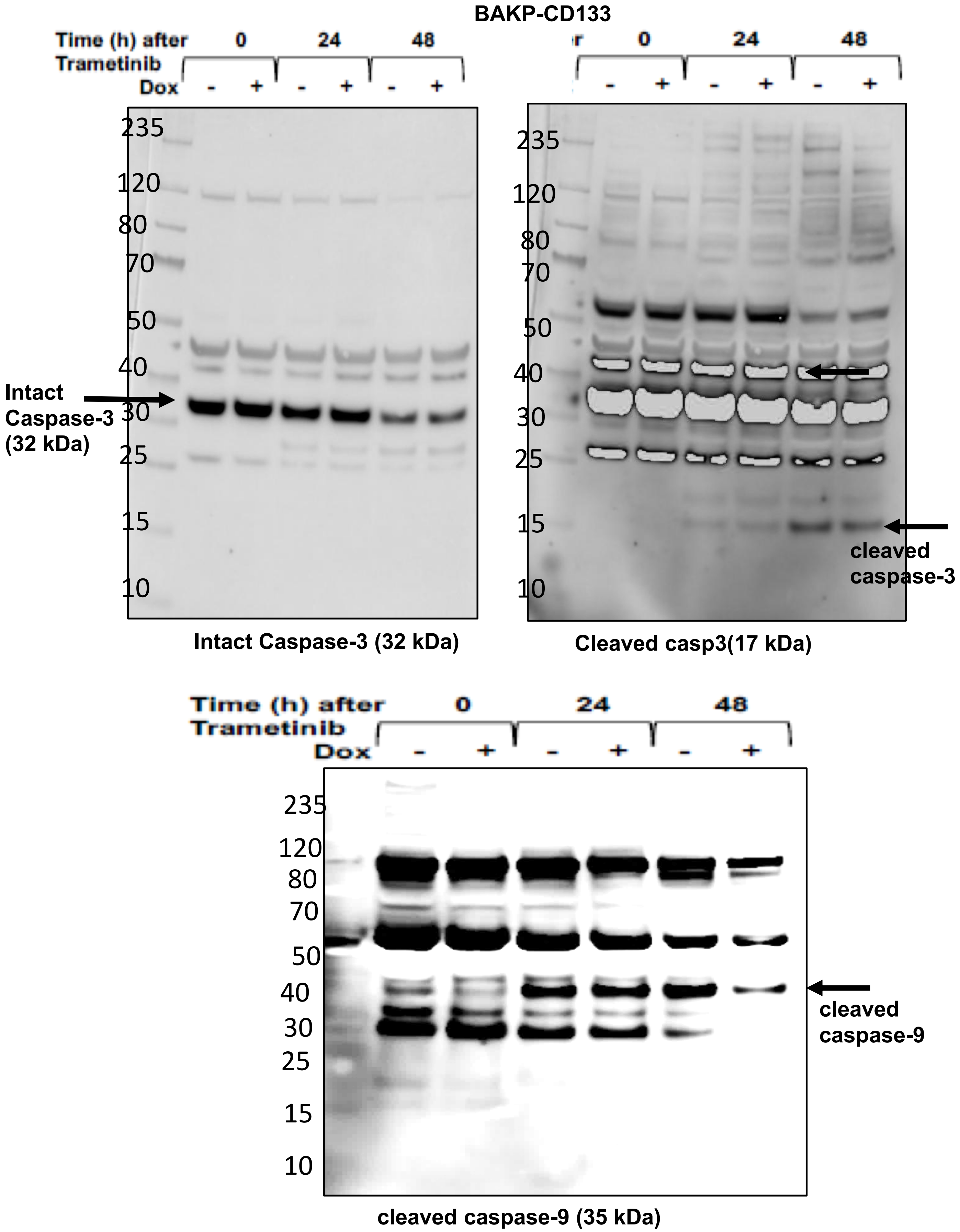
BAKP-CD133
Time (h) after Trametinib
0
24
48
Dox
- + - + - +
235
120
80
70
50
40
30
25
15
10
Intact Caspase-3 (32 kDa)
Intact Caspase-3 (32 kDa)
cleaved caspase-3
Cleaved casp3(17 kDa)
Time (h) after Trametinib
0
24
48
Dox
- + - + - +
cleaved caspase-9
cleaved caspase-9 (35 kDa)

Figure S6B

BAKP-CD133

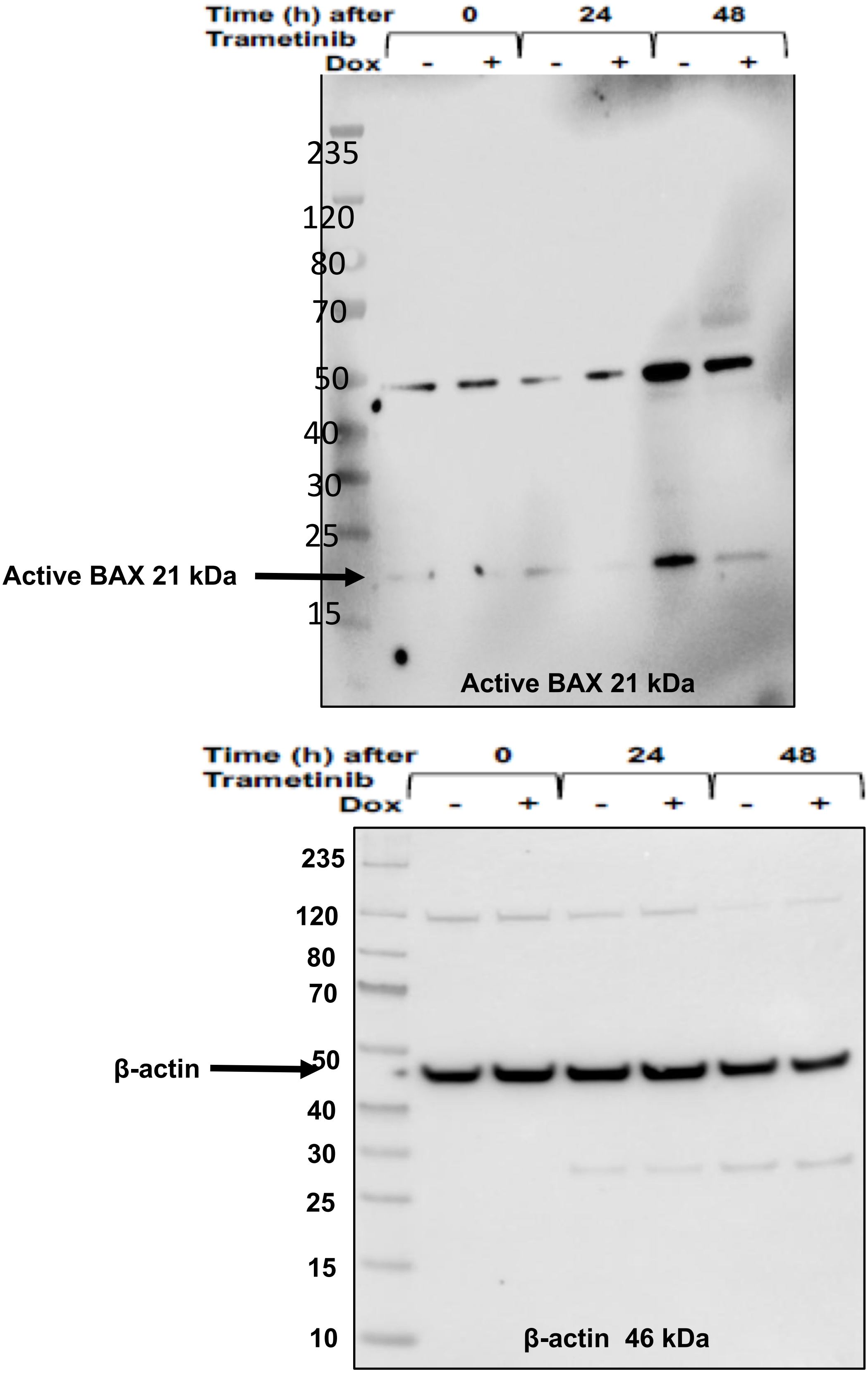

Figure S6C

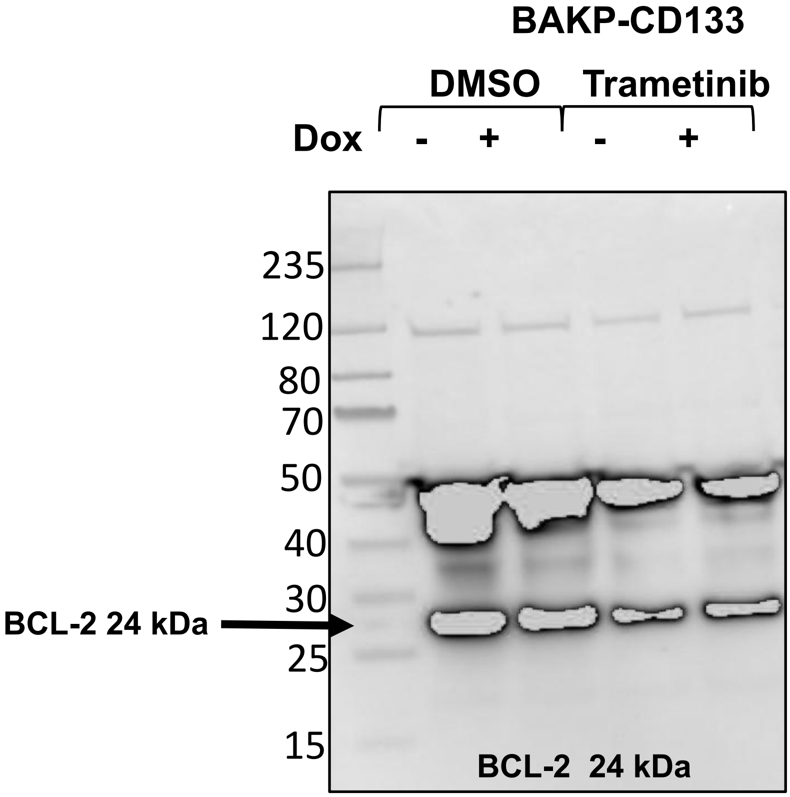
BAKP-CD133
DMSO
Trametinib
Dox
-
+
-
+
235
120
80
70
50
40
30
25
15
BCL-2 24 kDa
BCL-2 24 kDa

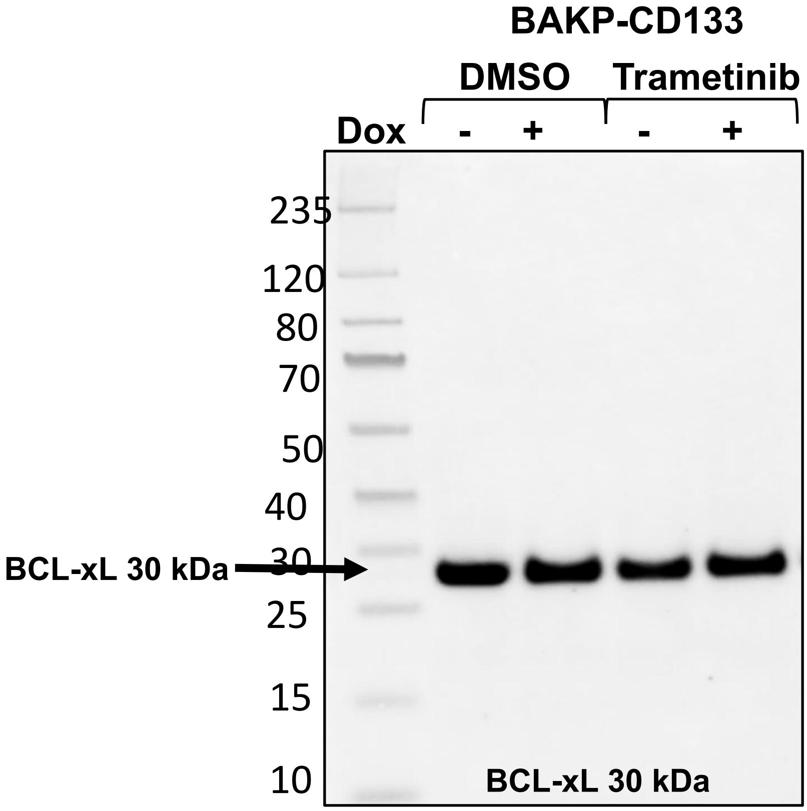
BAKP-CD133
DMSO
Trametinib
Dox
-
+
-
+
235
120
80
70
50
40
30
25
15
10
BCL-xL 30 kDa
BCL-xL 30 kDa

Figure S6D

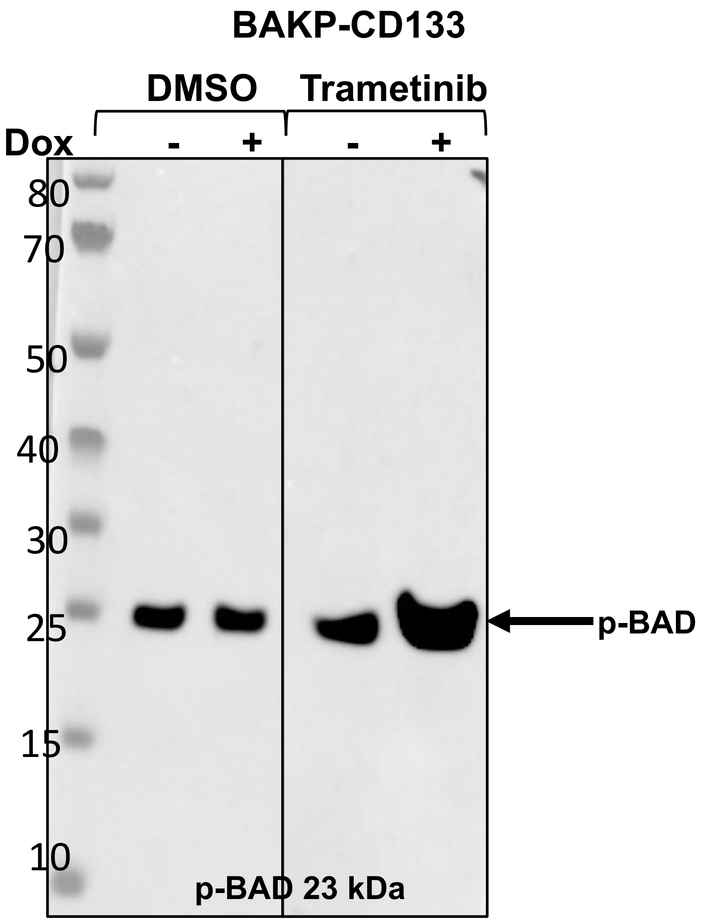
BAKP-CD133
DMSO
Trametinib
Dox
-
+
-
+
80
70
50
40
30
25
15
10
p-BAD
p-BAD 23 kDa

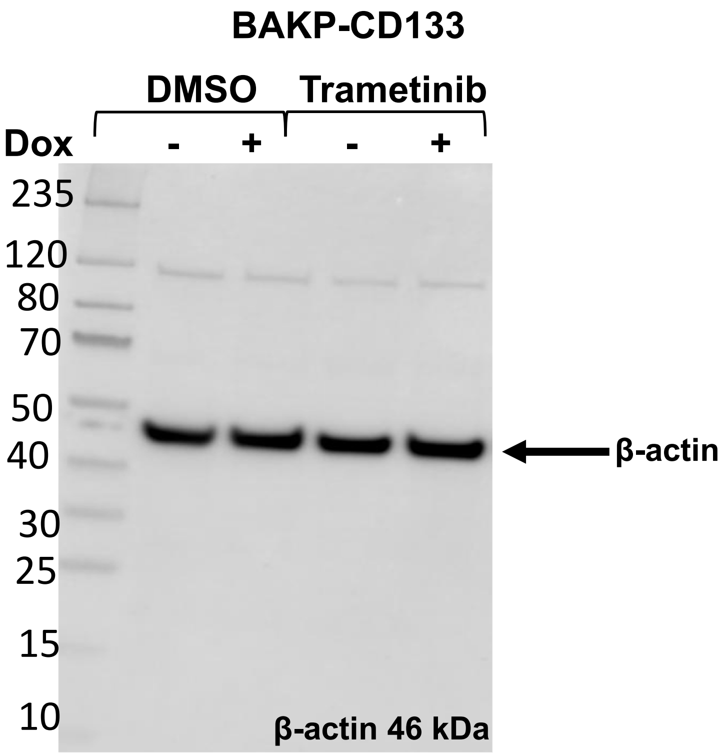
BAKP-CD133
DMSO
Trametinib
Dox
-
+
-
+
235
120
80
70
50
40
30
25
15
10
β-actin
β-actin 46 kDa

Figure S6E

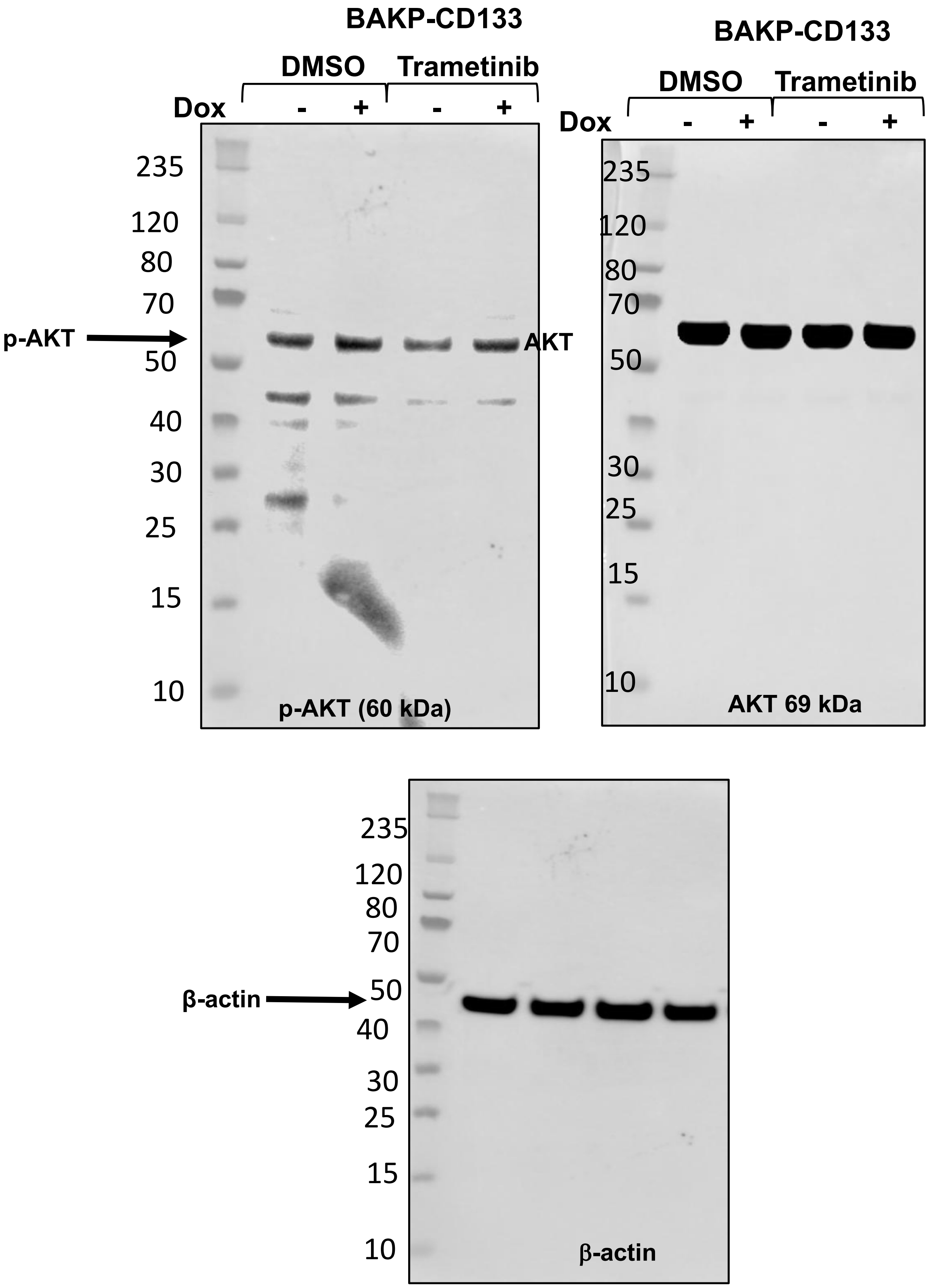
BAKP-CD133
DMSO
Trametinib
Dox
- + - +
235
120
80
70
p-AKT
50
40
30
25
15
10
AKT
p-AKT (60 kDa)
BAKP-CD133
DMSO
Trametinib
Dox
- + - +
235
120
80
70
50
30
25
15
10
AKT 69 kDa
235
120
80
70
β-actin
50
40
30
25
15
10
β-actin

Figure S6F

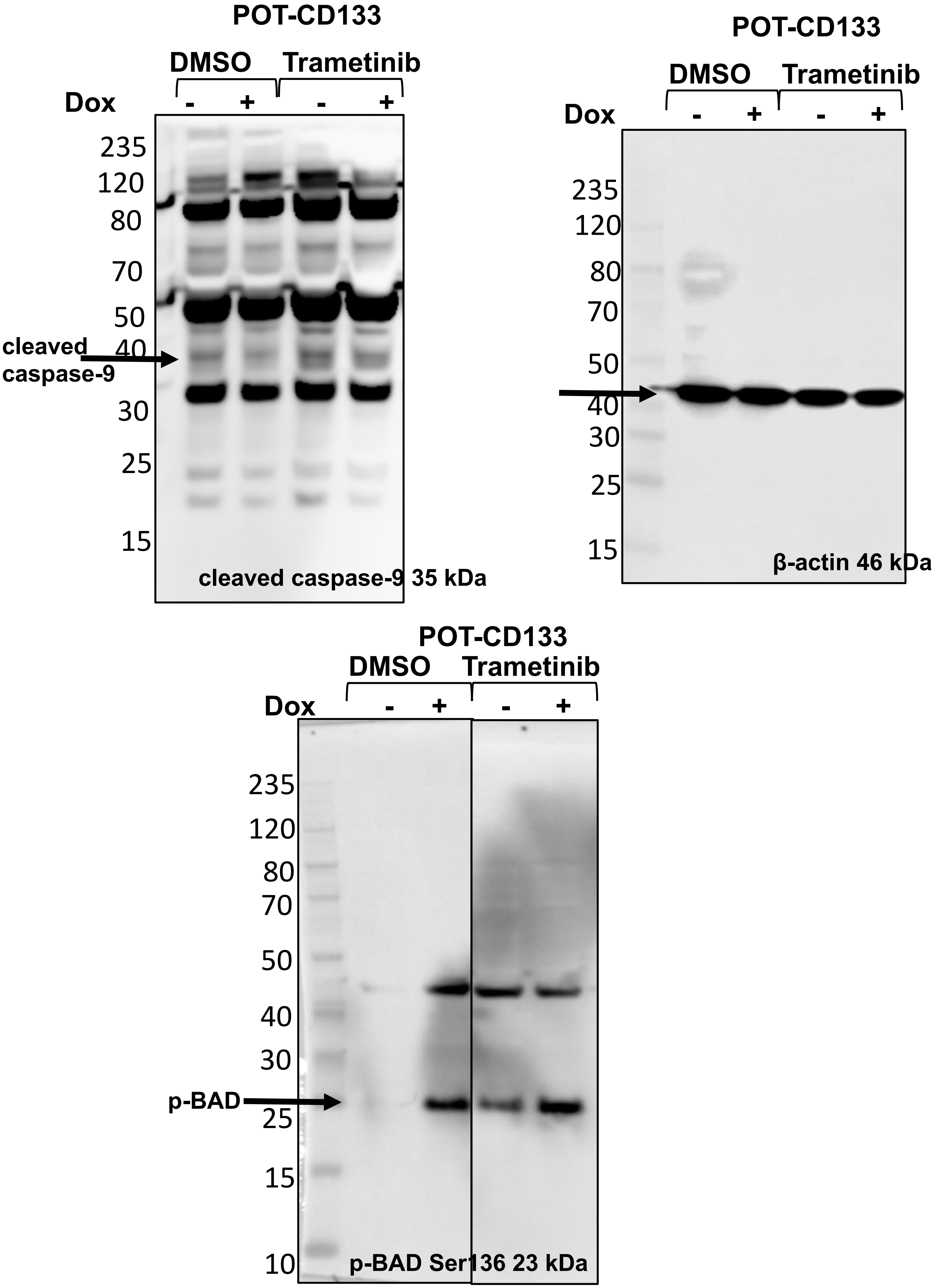
POT-CD133
DMSO
Trametinib
Dox
-
+
-
+
235
120
80
70
50
40
30
25
15
cleaved
caspase-9
cleaved caspase-9 35 kDa
POT-CD133
DMSO
Trametinib
Dox
-
+
-
+
235
120
80
70
50
40
30
25
15
β-actin 46 kDa
POT-CD133
DMSO
Trametinib
Dox
-
+
-
+
235
120
80
70
50
40
30
25
15
10
p-BAD
p-BAD Ser136 23 kDa

Figure S6G

POT-CD133

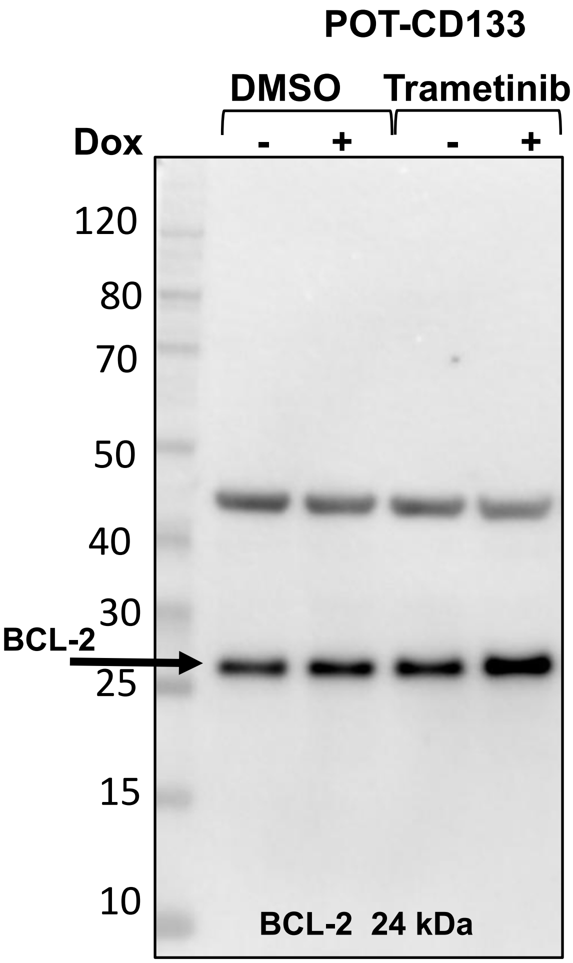

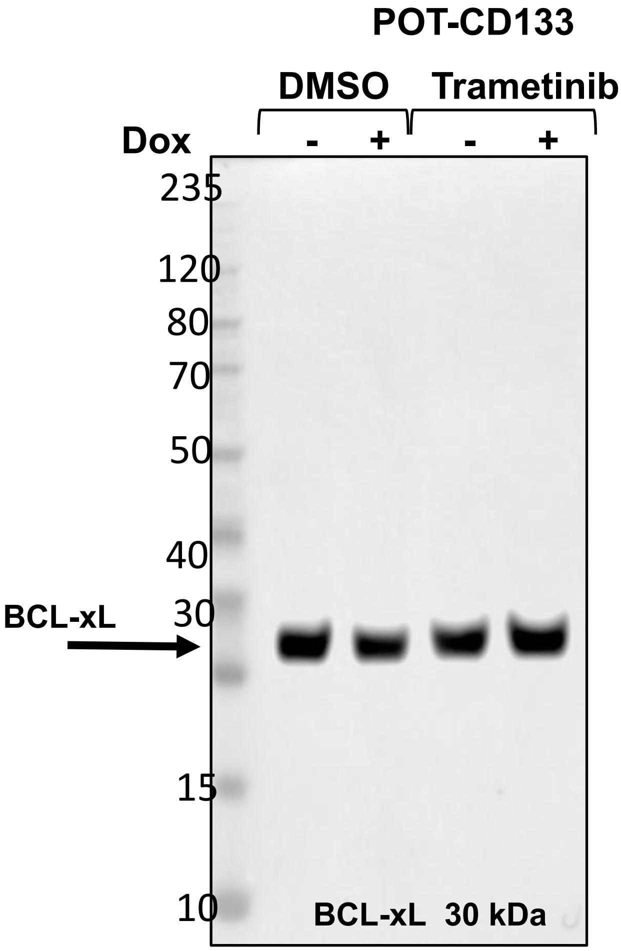

Figure S6H

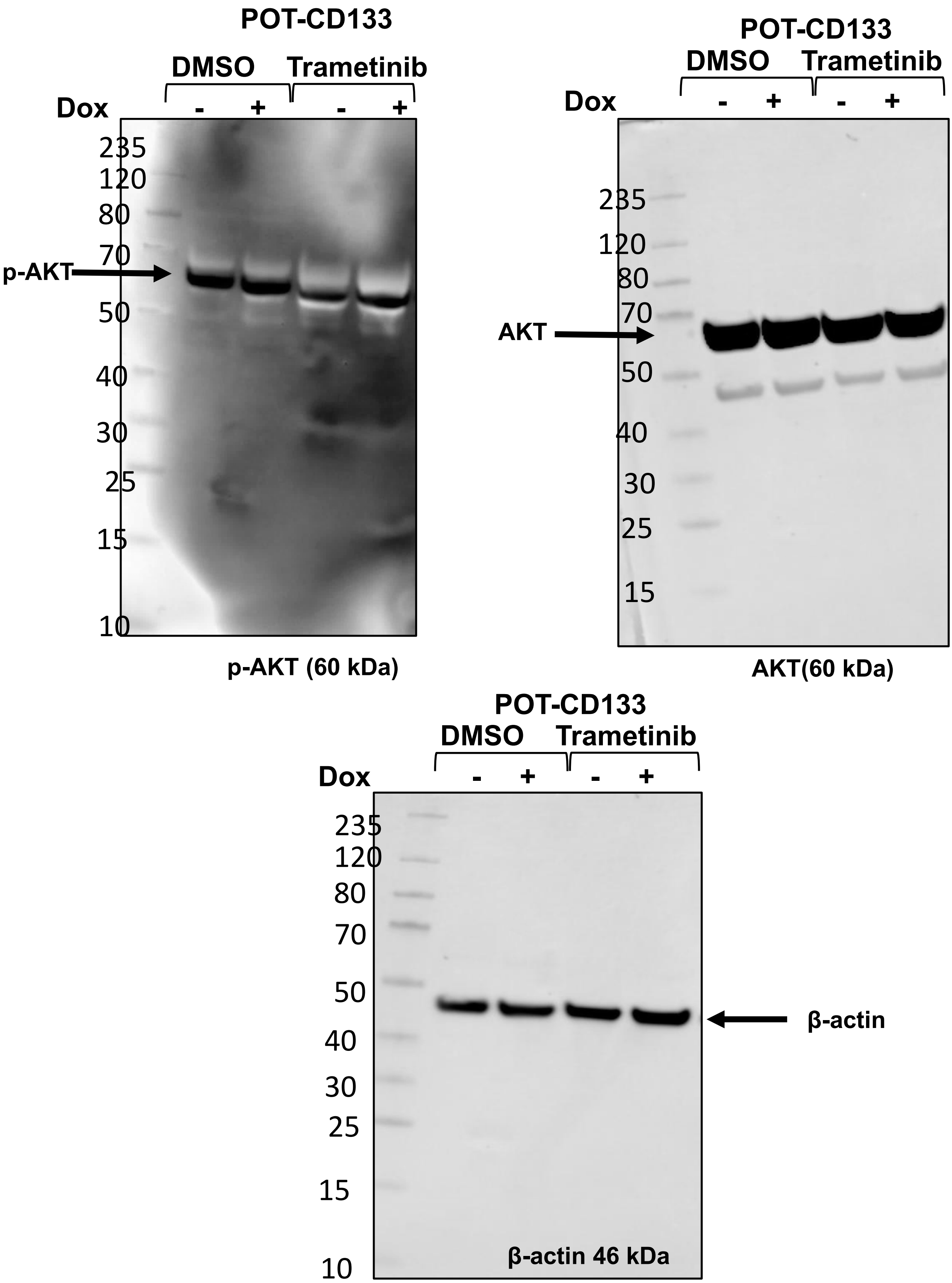

POT cells

Figure S6I

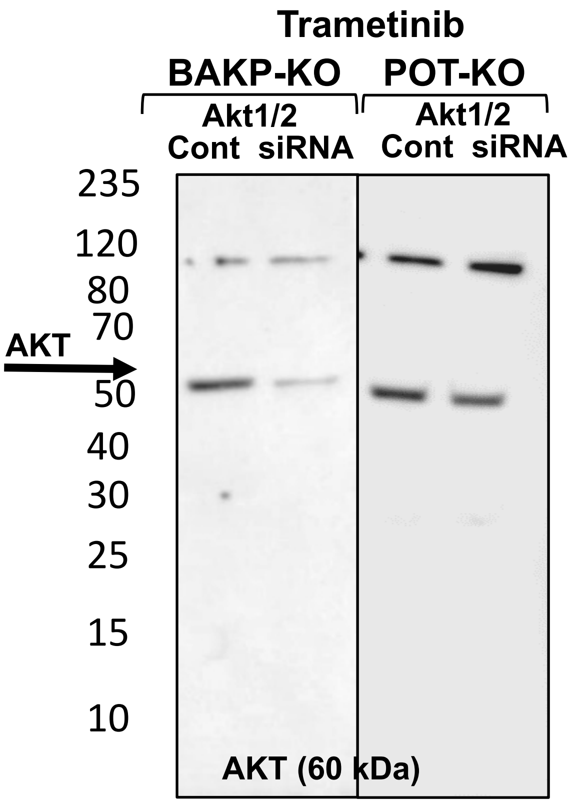
Trametinib
BAKP-KO
POT-KO
Akt1/2
Cont
siRNA
Akt1/2
Cont
siRNA
235
120
80
70
AKT
50
40
30
25
15
10
AKT (60 kDa)

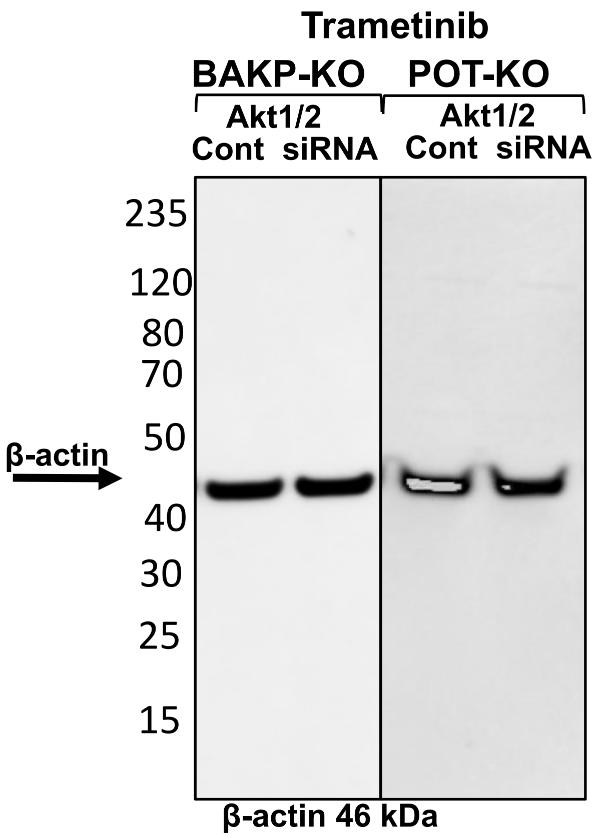
Trametinib
BAKP-KO
POT-KO
Akt1/2
Cont
siRNA
Akt1/2
Cont
siRNA
235
120
80
70
50
β-actin
40
30
25
15
β-actin 46 kDa

Figure S7A

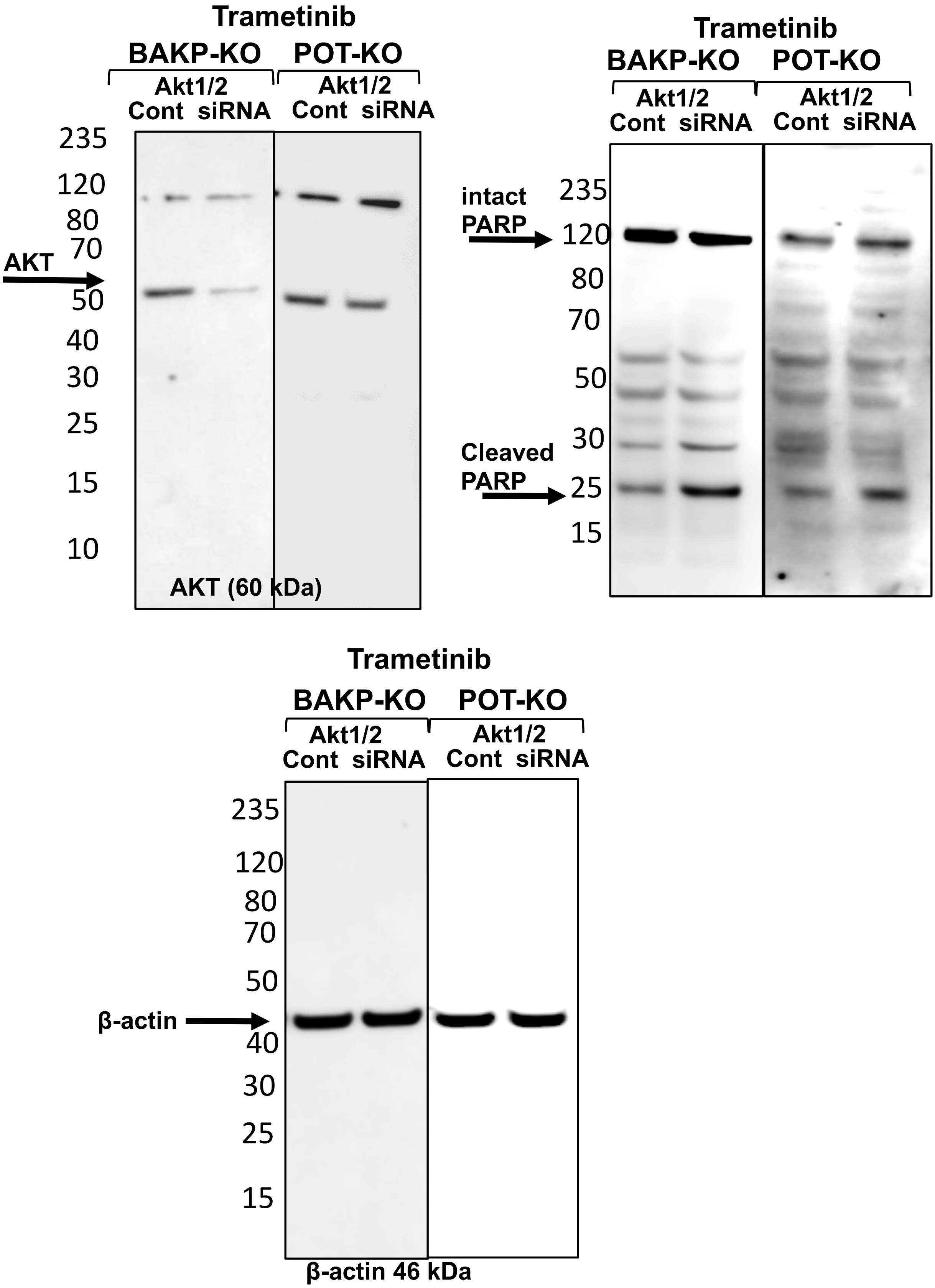
Trametinib
BAKP-KO
POT-KO
Akt1/2
Cont siRNA
Akt1/2
Cont siRNA
235
120
80
70
AKT
50
40
30
25
15
10
AKT (60 kDa)
Trametinib
BAKP-KO
POT-KO
Akt1/2
Cont siRNA
Akt1/2
Cont siRNA
intact
PARP
235
120
80
70
50
Cleaved
PARP
30
25
15
Trametinib
BAKP-KO
POT-KO
Akt1/2
Cont siRNA
Akt1/2
Cont siRNA
235
120
80
70
50
β-actin
40
30
25
15
β-actin 46 kDa

Figure S7B

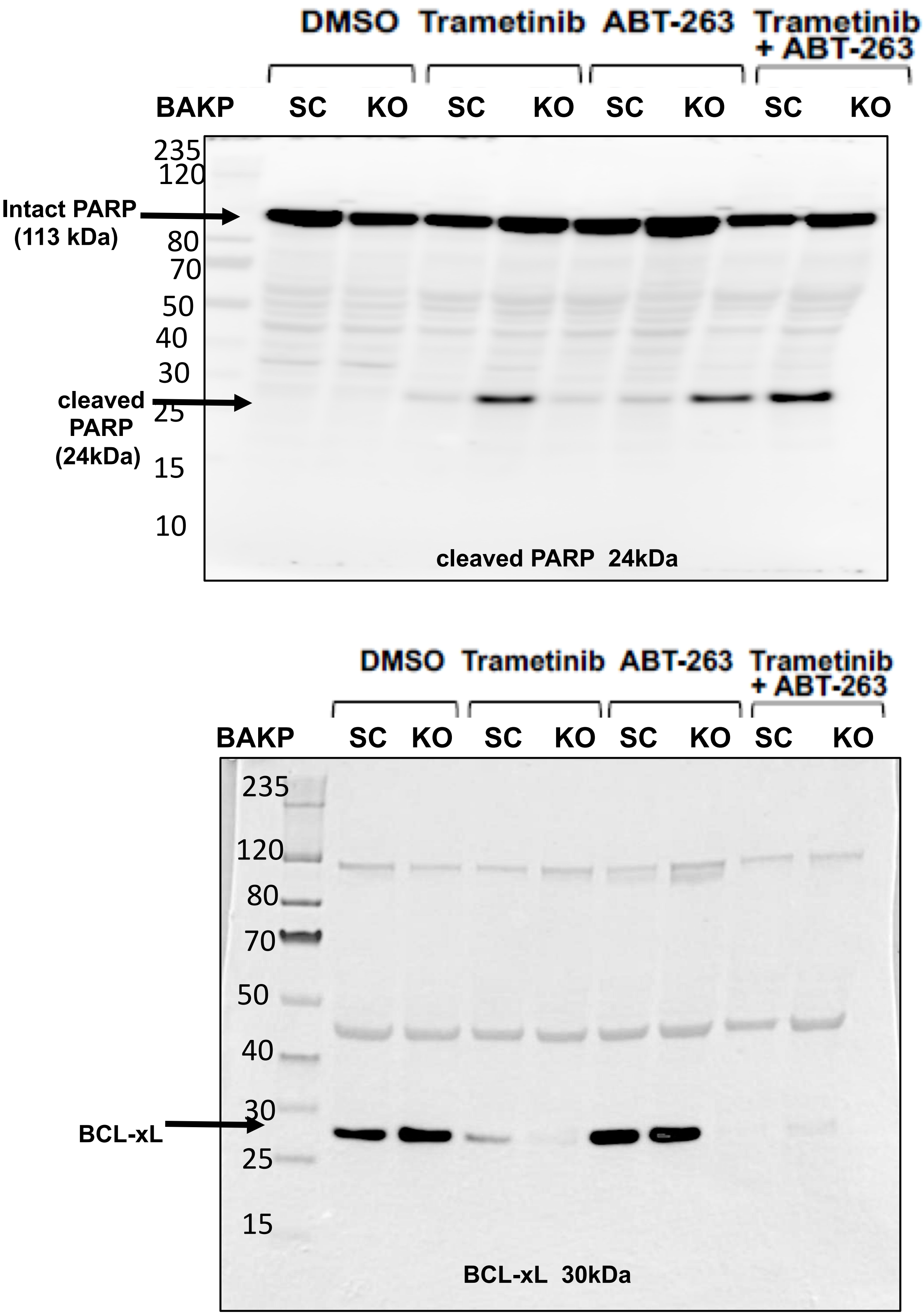
DMSO
Trametinib
ABT-263
Trametinib + ABT-263
BAKP
SC
KO
SC
KO
SC
KO
SC
KO
235
120
Intact PARP (113 kDa)
80
70
50
40
30
cleaved PARP (24kDa)
25
15
10
cleaved PARP 24kDa
DMSO
Trametinib
ABT-263
Trametinib + ABT-263
BAKP
SC
KO
SC
KO
SC
KO
SC
KO
235
120
80
70
50
40
30
BCL-xL
25
15
BCL-xL 30kDa

Figure S7C

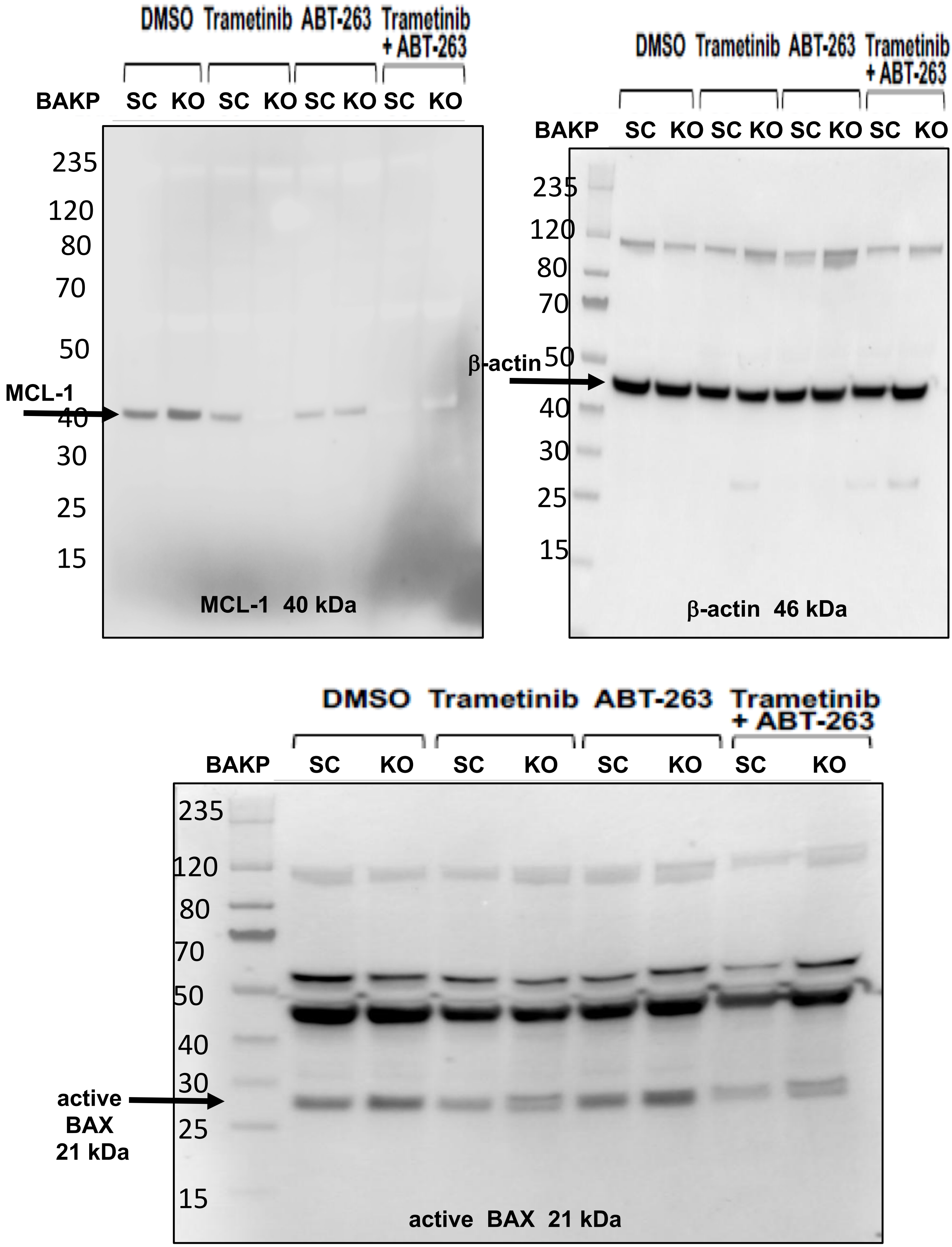
DMSO Trametinib ABT-263 Trametinib + ABT-263
BAKP SC KO SC KO SC KO SC KO
235
120
80
70
50
MCL-1
40
30
25
15
MCL-1 40 kDa
β-actin
β-actin 46 kDa
active BAX 21 kDa
active BAX 21 kDa

Figure S7D

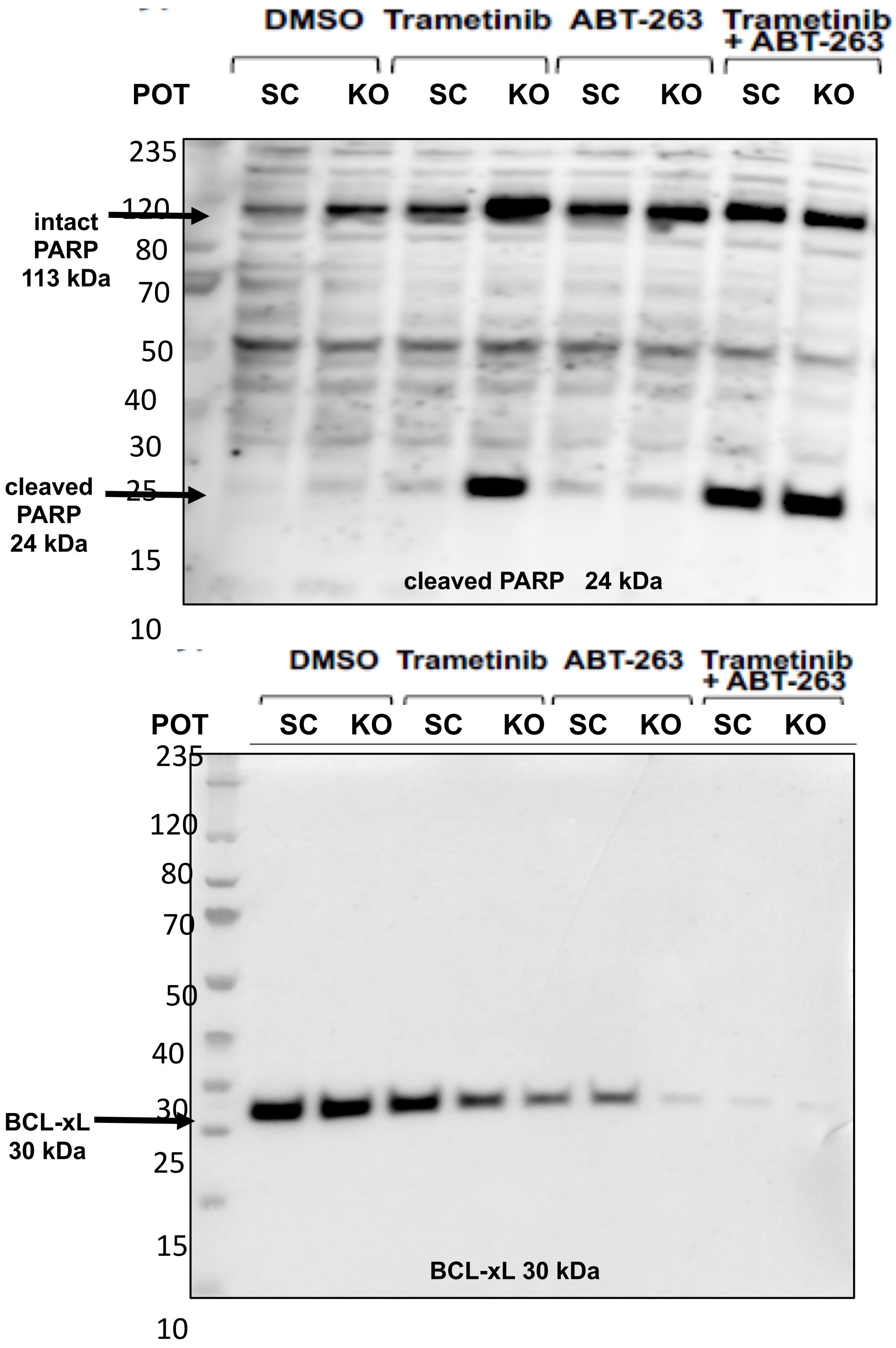
DMSO Trametinib ABT-263 Trametinib + ABT-263
POT SC KO SC KO SC KO SC KO
235
120
80
70
50
40
30
25
15
10
intact PARP 113 kDa
cleaved PARP 24 kDa
cleaved PARP 24 kDa
DMSO Trametinib ABT-263 Trametinib + ABT-263
POT SC KO SC KO SC KO SC KO
235
120
80
70
50
40
30
25
15
10
BCL-xL 30 kDa
BCL-xL 30 kDa

Figure S7E

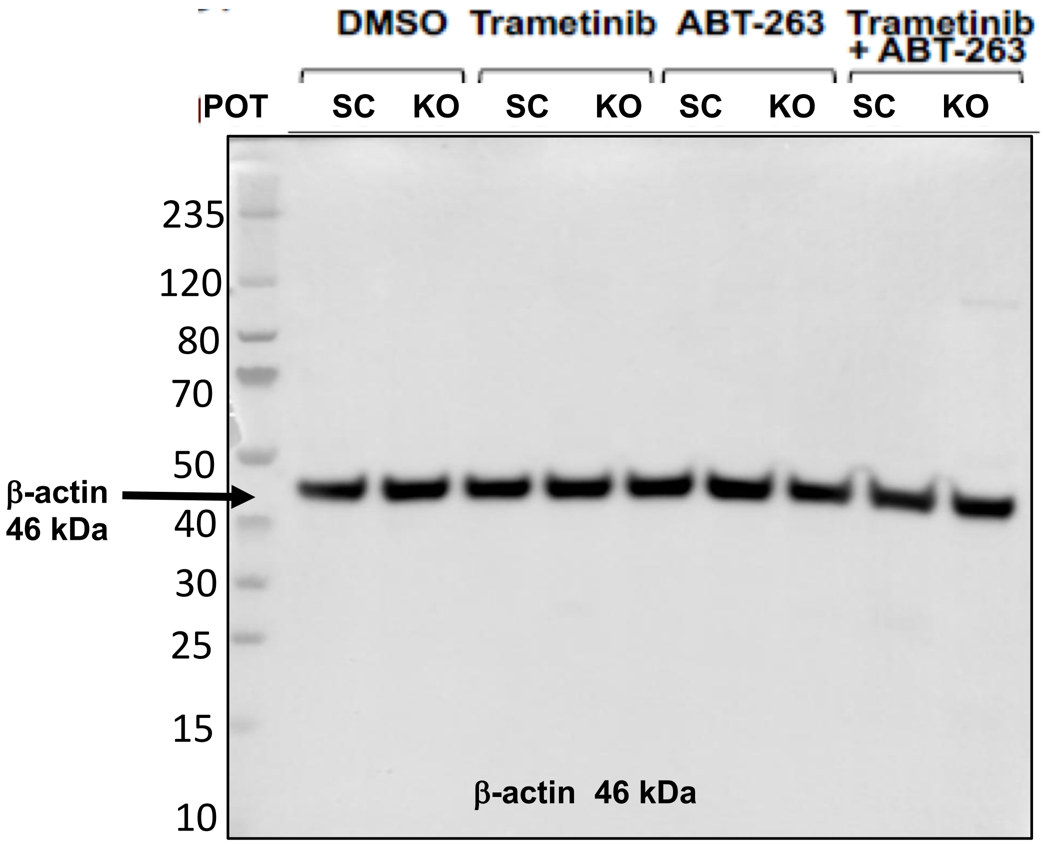
DMSO
Trametinib
ABT-263
Trametinib + ABT-263
POT
SC
KO
SC
KO
SC
KO
SC
KO
235
120
80
70
50
40
30
25
15
10
β-actin 46 kDa
β-actin 46 kDa

Figure S7F
